# *In situ* reduction of CO_2_ forms surface redox mediators on nanoparticle catalysts

**DOI:** 10.1039/d6sc05682b

**Published:** 2026-10-14

**Authors:** Sucharita Vijayaraghavan, Hung-Ling Yu, Adam J. Senko, Nishchay Rathi, Ayman M. Karim, David W. Flaherty

**Affiliations:** a School of Chemical and Biomolecular Engineering, Georgia Institute of Technology GA 30332 USA dflaherty3@gatech.edu (+404)-894-5922; b Department of Chemical Engineering, University of Virginia Charlottesville VA 22903 USA

## Abstract

Pathways and selectivities for oxygen reduction (ORR) on nanoparticle catalysts respond sensitively to the addition of exogenous redox mediators, and recent findings indicate organic surface redox mediators influence formation rates for hydrogen peroxide (H_2_O_2_) and water. Here, the introduction of carbon dioxide (CO_2_) increases both steady-state rates and selectivities to H_2_O_2_ during thermocatalytic oxygen reduction by H_2_ on nanoparticle catalysts in pure water. Contact with CO_2_ influences catalysis over Pd long after removal of gaseous CO_2_, whereas differences for Au nanoparticles revert immediately. These observations reflect the formation of carbonic acid, which increases proton activity and surface coverage of H*-atoms formed by the Volmer reaction. Furthermore, interpretation of kinetic isotope effects, *operando* spectroscopy, and open circuit potentiometry measurements indicate that persistent organic residues (*e.g.*, formate and methyl groups) form by CO_2_ hydrogenation on Pd nanoparticles and give rise to an increase in H_2_O_2_ formation rates (two-fold) and selectivities (55% to 90%). These surface redox mediators introduce pathways with distinct kinetic barriers for H_2_ activation and suppress transitions between metallic and hydridic phases. These findings provide new insights, revealing previously unrecognized roles of CO_2_ in modulating ORR pathways toward H_2_O_2_ formation and establish CO_2_-derived surface species as benign redox mediators.

## Abstract 2

CO_2_ enhances H_2_O_2_ formation by increasing proton concentration and generating persistent surface redox mediators on Pd nanoparticles, doubling H_2_O_2_ rates and increasing selectivity from 55% to 90%.

## Graphical abstract

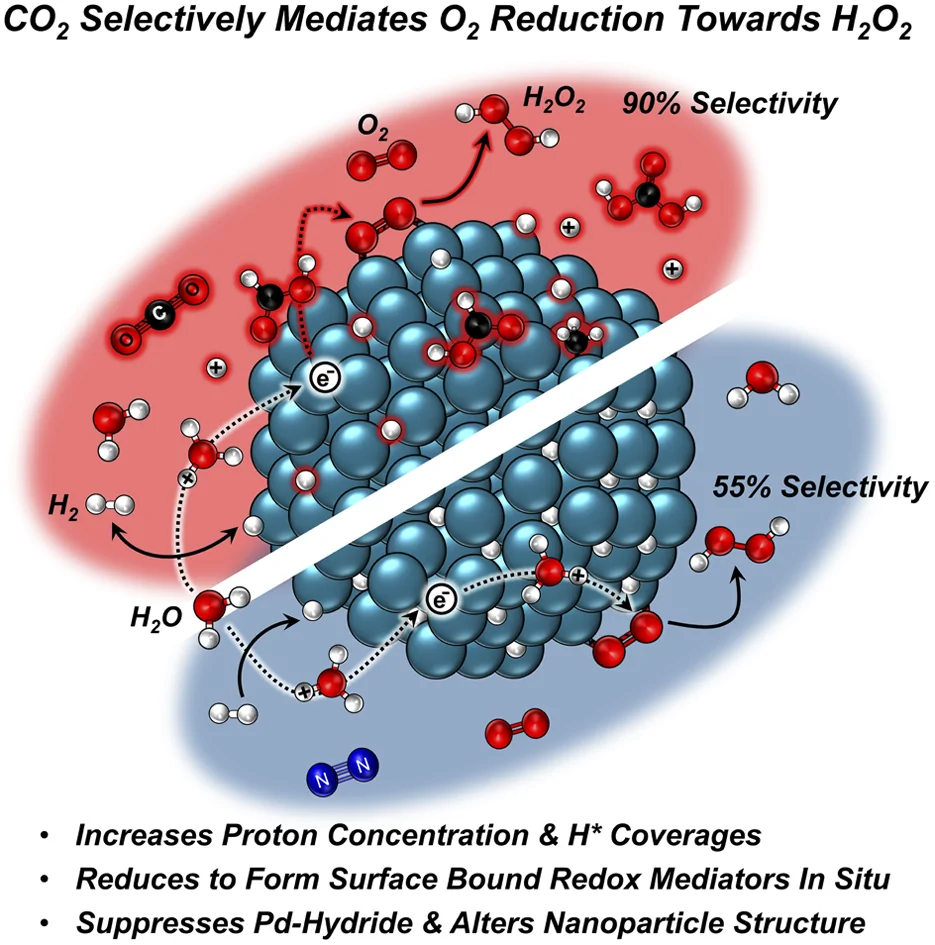

## 1. Introduction

Redox mediators facilitate electron (and proton) transfer processes in both electrochemical and thermochemical systems by providing alternative reaction pathways with lower overpotentials and activation barriers.^1^ In biological systems, quinones,^2^ nicotinamide cofactors (NAD^+^/NADH), and flavins (FAD/FADH_2_, FMN/FMNH_2_)^3^ function as ubiquitous redox mediators that shuttle electrons between spatially separated enzymes to promote a broad range of metabolic and biosensing processes.^4^ Redox mediators such as quinones,^5^ polyoxometalates (POMs),^6^ and transition-metal couples (*e.g.*, Fe^2+^/Fe^3+^, VO^2+^/VO^2+^)^7^ play analogous roles in electrochemical technologies by enabling indirect electron-transfer pathways between electrodes and reactants.^1^ Furthermore, solution-phase mediators decouple interfacial charge transfer from surface-bound reaction steps and offer the means to modulate reaction rates and selectivity, and access otherwise inaccessible catalytic mechanisms.

Redox mediators for oxygen reduction reactions (ORR) can overcome intrinsic kinetic limitations associated with direct O_2_ activation at electrode surfaces and circumvent the complexity of proton-coupled electron-transfer (PCET) pathways.^8^ Redox mediators (*e.g.*, organic nitroxyl radicals,^9^ quinones,^10^ and soluble transition-metal complexes^11–13^) reversibly shuttle protons and electrons to molecular oxygen through indirect solution-phase pathways, thereby decreasing the coverage of strongly adsorbed oxygenated intermediates that contribute to high overpotentials, catalyst poisoning, and degradation. Mediated ORR offers a particular advantage for tuning selectivity toward the two-electron reduction of O_2_ to hydrogen peroxide (H_2_O_2_), because solution-phase mediators stabilize hydroperoxyl (OOH˙) intermediates and suppress O–O bond dissociation that leads to the competing four-electron reduction of O_2_ to water (H_2_O) (*e.g.*, hydroquinone, tris{4-[2-(2-methoxyethoxy)ethoxy]phenyl}amine or TMPPA, 2,5-di-*tert*-butyl-*p*-benzoquinone or DTBBQ).^8,14^ Evidence from computation, spectroscopy, and rate measurements (*e.g.*, kinetic isotope effects, activation barriers) established that methanol, a commonly used solvent in thermochemical ORR, adsorbs on Pd surfaces and forms redox mediators (*i.e.*, hydroxymethyl) that facilitate PCET steps to O_2_ and lowers activation barriers for O_2_ reduction, thereby increasing H_2_O_2_ formation rates and selectivities.^15^ Similarly, polyconjugated carbonyl and aromatic compounds (*e.g.*, benzoquinone, hexaketocyclohexane) introduced *ex situ* serve as persistent surface-bound redox mediators that transfer proton–electron pairs to oxygen-derived intermediates in heterolytic pathways reminiscent of the methanol-derived intermediates.^16^

Recently, carbon dioxide and related derivatives (*e.g.*, carbonate radicals and percarbonates) emerged as effective mediators capable of steering electrochemical water oxidation pathways toward selective H_2_O_2_ production by altering reaction mechanisms and intermediate energetics.^17–19^ Hâncu and Beckman first reported that the introduction of CO_2_ during thermochemical ORR increased H_2_O_2_ formation rates in efforts that explored the use of supercritical CO_2_ as a diluent to avoid forming explosive mixtures of H_2_ and O_2_ and increase gas solubility.^20,21^ Hutchings *et al.* demonstrated that the decrease in solution pH upon CO_2_ addition to aqueous–organic solvent mixtures increases H_2_O_2_ yields by suppressing secondary reactions that hydrogenate H_2_O_2_.^22,23^ These explanations focus upon the contributions of non-catalytic pathways and the influence of CO_2_ and CO_2_-derived intermediates in surface chemistry remains largely unexplored.

Here, we demonstrate that the addition of gaseous CO_2_ into thermochemical ORR environments alters the reaction microenvironment at solid–liquid interfaces, modifies the coverage of reactive species, and influences the structure of Pd and Au nanoparticle catalysts. On contact with water, CO_2_ forms carbonic acid, lowers the pH, and increases the coverage of hydrogen atoms on both Pd and Au through Volmer steps. These processes increase H_2_O_2_ formation rates by two-fold on both catalysts. However, Pd surfaces also hydrogenate CO_2_ to generate surface-bound organic species that function as redox mediators, facilitate distinct PCET pathways, and contribute to significant gains in H_2_O_2_ selectivities (55% to 90%) that persist for extended periods after removal of CO_2_ from the reactor (>10 h). *Operando* spectroscopy, open circuit potentiometry, and kinetic analyses give evidence that these persistent CO_2_-derived intermediates block unselective sites, suppress Pd hydride (β-PdH_*x*_) formation, and alter the nature of kinetically relevant steps. Together, these findings reveal the surface reactions responsible for the promotional and redox mediation effects of CO_2_ in thermochemical ORR that result in 90% H_2_O_2_ selectivity on unmodified Pd nanoparticle catalysts. More broadly, this work highlights the potential of CO_2_ to serve not only as a process-safety diluent that improves gas solubility but also as a process and environmentally friendly redox mediator that enhances formation rates and selectivity for long-term, stable H_2_O_2_ production.

## 2. Experimental methods

### 2.1. Synthesis of Pd and Au nanoparticles supported on silica

Pd catalysts supported on silica (SiO_2_) were synthesized by strong electrostatic adsorption (SEA) of Pd(NH_3_)_4_^2+^ complexes onto fumed SiO_2_ (CAB-O-SIL M-5).^24^ A 0.1 mM aqueous Pd precursor solution was prepared using tetraamminepalladium(ii) chloride monohydrate (Pd(NH_3_)_4_Cl_2_·H_2_O; Sigma-Aldrich, >99.99% trace metal basis). Prior to Pd complex adsorption, 10 g of SiO_2_ was soaked in an aqueous solution of NH_4_OH (0.5 M, 300 cm^3^; Sigma-Aldrich, 30–33% in H_2_O) for 10 min and stirred at 500 rpm. The 0.1 mM solution containing Pd(NH_3_)_4_^2+^ complexes was added to the SiO_2_ mixture and stirred for 24 h to allow SEA to approach equilibrium. The resulting solution was centrifuged in 50 cm^3^ centrifuge tubes at 10 000 rpm for 20 min to recover wet solids which were dried at 333 K for 72 h in a static drying oven. The dried solids were reduced under a flowing mixture of hydrogen (20 kPa H_2_, 80 kPa Ar, 250 cm^3^ min^−1^) at 573 K for 3 h in a quartz tube horizontal furnace. Once cooled, the catalysts were passivated in ambient air. Prior work established that a 0.05 wt% Pd loading or less is sufficient to avoid intrapellet mass transfer limitations for thermocatalytic O_2_ reduction to form H_2_O_2_.^25^

Au catalysts supported on SiO_2_ were synthesized by SEA of gold bis-ethylenediamine (Au(en)_2_Cl_3_) onto fumed SiO_2_.^26^ The Au(en)_2_Cl_3_ precursor was prepared by first dissolving 1 g of HAuCl_4_ (Sigma-Aldrich, ≥99.9% trace metal basis) in diethyl ether (10 cm^3^; Sigma-Aldrich, ≥99.9%, HPLC-grade, inhibitor free). Separately, a solution of ethylene diamine (1 cm^3^; Sigma-Aldrich, ≥99.9%, ReagentPlus®) and diethyl ether (5 cm^3^) was prepared and added to the solution containing HAuCl_4_. The liquid after letting the mixture separate was decanted, and the remaining gummy yellow precipitate was dissolved in 2.8 cm^3^ of milliQ water (∼18.2 MΩ cm). The Au(en)_2_Cl_3_ was then precipitated as a white solid with ethyl alcohol (20 cm^3^, Fisher Scientific, 200 Proof, Molecular Biology Grade). The liquid was decanted and redispersed in water (2.3 cm^3^) and precipitated once more with ethyl alcohol (15 cm^3^). The final precipitate of Au(en)_2_Cl_3_ was vacuum filtered and dried at ambient temperature (298 K) for 48 h.^27^ An aqueous solution of the dried Au(en)_2_Cl_3_ precursor was prepared (0.6 mM) and its pH adjusted to 11 using NH_4_OH (0.5 M) and stirred at 850 rpm with an overhead stirrer at 298 K for 20 min. To this, 10 g of SiO_2_ was added and stirred for 1 h at 1000 rpm, after which the mixture was centrifuged at 10 000 rpm for 20 min to recover wet solids which were dried at 333 K for 72 h in a static drying oven. The dried solids were reduced under a flowing mixture of hydrogen (20 kPa H_2_, 80 kPa Ar, 250 cm^3^ min^−1^) at 673 K for 1 h in a quartz tube horizontal furnace.

Metal loadings for all catalysts as synthesized and after exposure to reaction conditions were determined by ICP-OES and are listed in Table S1.

### 2.2. Nanoparticle characterization

The average nanoparticle diameter and particle size distribution of Pd and Au catalysts supported on SiO_2_ was determined by bright-field transmission electron microscopy (TEM; FEI, Tecnai F30) operating at 300 kV and averaging more than 100 nanoparticles. The number averaged mean diameter (〈*d*〉_*N*_) was calculated by numerical averaging of the measured diameters, and the surface area normalized mean diameter (〈*d*〉_*S*_) was calculated according to the following equation,1
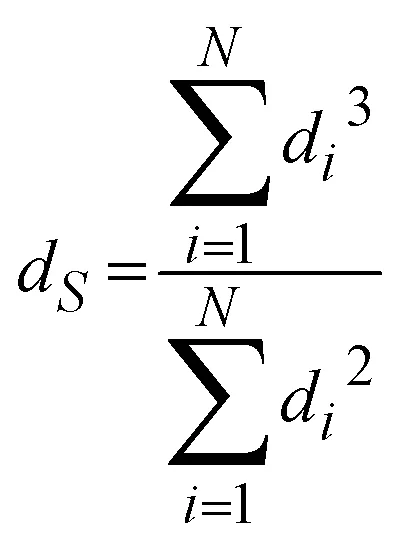
where *d*_*i*_ is the measured diameter of each nanoparticle *i* from TEM images and *N* is the total number of nanoparticles measured for each sample.

The Pd dispersion and nanoparticle size of Pd catalysts was also estimated using pulse CO chemisorption measurements (Micromeritics, AutoChem III). Approximately 100 mg of sample was loaded into a quartz U-tube. Both the fresh and spent samples were heated in 10 kPa H_2_ to 573 K at 10 K s^−1^, held at 573 K for 1 h under 10 kPa H_2_, and purged with 101 kPa He at 573 K for 1 hour before cooling to 298 K under He. Pulses of dilute CO (10% CO in He, Airgas) were introduced and the amount of CO not adsorbed onto the Pd was detected by a thermal conductivity detector. A stoichiometry coefficient of 0.5 CO/Pd was used to account for an average of the different binding modes of CO to Pd atoms.

X-ray diffraction (XRD) patterns of the as-synthesized and spent catalysts were obtained using a diffractometer (Rigaku Miniflex Powder XRD) with a Cu Kα radiation source (*λ* = 0.15418 nm) in ambient conditions to obtain information on changes to the catalyst structure after exposure to thermochemical O_2_ reduction reaction conditions involving CO_2_.

Infrared spectroscopy of adsorbed CO (CO*) was utilized to confirm the surface structure of Pd and Au catalysts. The measurements were performed according to methods described in previous works.^24,28,29^ All samples were first pretreated under oxidative conditions (20 kPa O_2_, 81 kPa Ar, 30 cm^3^ min^−1^, 573 K, 1 h) to remove any adventitious organic species and water, and subsequently reduced (20 kPa H_2_, 81 kPa Ar, 30 cm^3^ min^−1^, 423 K, 1 h) and cooled to 303 K in Ar before obtaining background spectra and flowing CO.

Section S1 of the SI presents nanoparticle characterization results.

### 2.3. Steady-state reaction rate measurements in a trickle bed reactor

Steady-state product formation rates (H_2_O_2_ and H_2_O) for the thermochemical oxygen reduction reaction were measured in a continuous-flow trickle bed reactor system (Fig. S5) described in detail in previous works.^25^ A total catalyst mixture of 2 g was loaded in a stainless-steel jacketed reactor (48 cm length, 1 cm inner diameter) supported by glass wool and glass rods. Pd catalyst was co-pelletized (35–60 mesh, 250 µm) with inert SiO_2_ gel (Davisil 646, 35–60 mesh) in a 1 : 3 dilution (active catalyst: SiO_2_ gel) and physically mixed with SiO_2_ gel to eliminate intrapellet mass transfer limitations according to the Koros–Nowak criterion.^30^ The Au catalyst was also pelletized (35–60 mesh, 250 µm) and loaded into the reactor but without any dilution. Reactant gases (25% H_2_/N_2_, 99.9% D_2,_ 5% O_2_/N_2_, 5% O_2_/CO_2_, Airgas, 99.999%) were introduced into the reactor using digital mass flow controllers (Bronkhorst, F-211CV) and mixed with a stream of degassed milliQ water (∼18.2 MΩ cm under Ar flow) supplied by a HPLC pump (SSI, LS class). A back-pressure regulator (Equilibar, LF) controlled by an electronic pressure regulator (Equilibar, GPI) was used to maintain the pressure of the gas–liquid mixture flowing through the reactor and the upstream reactor pressure was monitored using a digital pressure transducer (Omega, PXM409-USBH). A K-type thermocouple in contact with the reactor wall was used to monitor the reactor temperature which was controlled by flowing a 50/50 (v/v) mixture of water and ethylene glycol (Fisher Scientific E178, 99.8%) from a recirculating chiller (Cole-Parmer Polystat). The reactor effluent stream passed through a polycarbonate and polyvinyl chloride gas–liquid separator, where the gas stream was analyzed by a gas chromatograph (Agilent, 7890B, thermal conductivity detector, Ar reference gas) equipped with a capillary column (Vici, Molecular Sieve 5 Å, 30 m × 0.53 mm × 20 µm) to quantify reactant conversion and consumption rates. The liquid stream from the gas–liquid separator was drained every 10 minutes by an electronic valve (Banjo Corp., LEV025PL) and pulled into an electronic two-position 10-port valve (Vici Valco), where 1 cm^3^ of sample and 1 cm^3^ of a colorimetric titrant (12 mM neocuproine [Sigma-Aldrich, >99%], 8.3 mM CuSO_4_ [Fisher Scientific, >98.6%], 25/75 (v/v) ethanol/deionized water mixture [Decon Laboratories, >99.9%]) were injected into test tubes held in an automated fraction collector (Biorad, 2110). The concentration of H_2_O_2_ and H_2_O_2_ formation rates were determined by analyzing the test tubes using a UV-vis spectrophotometer (Spectronic, Genesys 20, 454 nm) using corresponding calibration curves.

Reported steady-state kinetics were measured at a liquid flow of 35 cm^3^ min^−1^, where reaction rates are independent of the fluid space velocity and occur in the absence of external mass transfer limitations (Fig. S7).^31^ Furthermore, H_2_ conversions were maintained between 2 and 6% to prevent secondary reactions of H_2_O_2_ and allow for the measurement of intrinsic kinetics. H_2_O_2_ selectivities were calculated by taking the ratio of H_2_O_2_ formation rates and total H_2_ consumption rates 
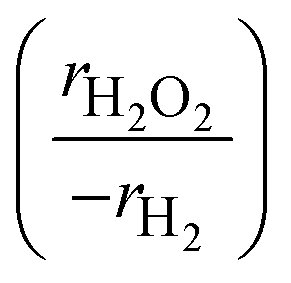
, which assumes all of the H_2_ consumed goes towards forming either the desired product, H_2_O_2_ or H_2_O. All steady-state measurements were obtained after an initial catalyst break-in (200 kPa H_2_, 60 kPa O_2_, 0 kPa CO_2_) of at least 20 h time on stream, which involved cycling the reaction temperature (278–308 K) to allow nanoparticles to activate and achieve stable rates and selectivities as well as to wash away any residual ions or ligands from synthesis with at least 40 L of milliQ H_2_O (Fig. S6). Formation rates from activation enthalpy and reactant pressure dependence measurements were examined after achieving steady-state conversion under each condition (1.5 and 3 h respectively), and corrected for catalyst deactivation by examining rates when the system was brought to the original reaction condition in the respective measurements and applying corrections through linear interpolation of time-on-stream rates assuming an exponential decrease in the number of active sites over time.^32^ HD formation rates in isotope scrambling experiments were measured with a mass spectrometer (Pfeifer, Thermostar Gas Analysis System).

Spent catalyst analysis post exposure to CO_2_ shows no evidence of metal leaching for both Pd and Au catalysts (Table S1). Nanoparticle sizes estimated from TEM images and CO chemisorption measurements show nanoparticles increase in size under O_2_ reduction reaction conditions, with no significant change in the presence of CO_2_ (Table S1, Fig. S2). XRD reflections of Pd and Au nanoparticles in the presence of CO_2_ resemble the XRD reflections of the as-synthesized nanoparticles and do not show evidence of carbide phase or modifications to the crystalline structure (Fig. S3).

### 2.4. Transient rate measurements in a semi-batch reactor

Transient H_2_O_2_ concentration profiles were measured in 100 cm^3^ three-neck round-bottom flasks. One port of the round-bottom flask was used to supply reactant gas mixtures (99.999% H_2_ and 5% O_2_/N_2_, 5% O_2_/CO_2_, Airgas) through a gas dispersion tube (40–60 µm) using digital MFCs (Alicat, MC series). The middle port of the flask was connected to a condenser, and the third port was connected to a stopcock that allowed solution sampling using a 3 cm^3^ syringe. MilliQ water and deuterated water (D_2_O; Thermo Fisher, 99.8 atom% D) were used. All experiments were conducted at ambient temperature and pressure, with the solution stirred at 1500 rpm using magnetic stir bars to avoid external mass transfer limitations.^15,31^ The solvent solution was saturated with reaction gases for 20 min before adding the catalyst to the flask and initiating the reaction. 50 mg of crushed Pd catalysts and 200 mg of crushed Au catalyst were used per measurement. 1 cm^3^ aliquots of the reaction mixture were extracted every 5 min for the first 30 min, every 10 min from 30–60 min of reaction time, and every hour after that for 3 h. These samples were titrated with 1 cm^3^ copper sulfate-neocuproine colorimetric indicator solution to determine H_2_O_2_ concentrations (similar to steady-state H_2_O_2_ rate measurements outlined in Section 2.3). The resulting H_2_O_2_ concentration *versus* time profiles were fit to the semi-batch reactor design equation to determine apparent H_2_O_2_ formation and decomposition rate constants using methods described in prior works.^15,24^

### 2.5. Catalyst structure analysis from *operando* X-ray absorption spectroscopy

The coordination of atoms in SiO_2_-supported Pd and Au catalysts and changes in the electronic properties and local structure under thermochemical oxygen reduction reaction conditions were examined by X-ray absorption near edge structure (XANES) and extended X-ray absorption fine structure (EXAFS) measurements at the Pd-K edge (24 350 eV) and Au-L_2_ (13 734 eV) and Au-L_3_ edges (11 919 eV). The measurements were performed on beamline 9–3 at the Stanford Synchrotron Radiation Light Source (SSRL) and using a 100-element Ge monolith fluorescence detector (Canberra) for Pd samples. The 9–3 beamline is a 16-pole, 2 Tesla wiggler side station with a vertically collimating mirror for harmonic rejection and a cylindrically bent mirror for focusing. The photon energy was chosen using a liquid-nitrogen cooled, double-crystal Si (220) *φ* = 90^°^. The catalyst was pelletized and sieved without dilution into pellets in the size range of 106–150 µm. The pellets were packed into a glassy carbon tube (6.35 mm OD, 4.1 mm ID) or quartz capillary (3.05 mm OD, 2.5 mm ID) for liquid and gas phase measurements, respectively. The packed bed is approximately 1 cm long and fixed in place with quartz wool on the top and bottom. The glassy carbon tube is mounted in a custom stainless steel cell with an X-ray window (10 mm horizontal × 5 mm vertical) for transmission and fluorescence measurements described in detail in previously published works.^33^ The quartz capillary tube is mounted on a cell designed by the CO-ACCESS group at SSRL.^34^ For the gas phase measurements, the Pd catalyst is first oxidized under 20 kPa O_2_ at 573 K for 1 hour, cooled to 298 K under He, then reduced under 20 kPa H_2_ at 423 K for 1 hour and cooled to 298 K under He; the spectra were collected under He after reduction. The catalysts for the liquid phase measurement were treated under liquid-phase O_2_ reduction reaction conditions (200 kPa H_2_, 60 kPa O_2_, 278 K) for 24 hours, dried under vacuum at 298 K, and exposed to air before the *in situ* measurements. The catalysts were loaded in the glassy carbon tube and exposed to gas saturated DI water without further pre-treatment.

The glassy carbon tube with catalyst was sealed in the *in situ* XAS cell, attached to a manifold used to deliver H_2_O saturated with gases (UHP H_2_, O_2_, CO_2_, Airgas) at elevated pressures.^15^ The gas compositions were controlled by flowing reactants (H_2_ and O_2_, or H_2_ and O_2_–CO_2_) into two pressurized saturation units (2L, Swagelok 304L-HDF8-2250) equipped with MFCs for each gas (Bronkhorst, F-211CV), which allowed gas to flow through gas dispersion frits (40–60 µm) into the saturation units containing milliQ water. The pressure in each saturation unit was maintained using back-pressure regulators (Andon Specialties, BP3-1A11QCK11L) and each unit was connected to two HPLC pumps (SSI, LS class) which pumped the gas saturated solvent from each unit to a single outlet stream, creating a H_2_/O_2_ (and CO_2_) saturated liquid. This saturated liquid stream passed through the *in situ* XAS cell and the cell was pressurized above the saturation unit pressures using a back-pressure regulator. The effluent stream from the reactor was analyzed with the same colorimetric titrant used in transient and steady-state rate measurements to determine *operando* H_2_O_2_ concentrations and formation rates. All XAS measurements were conducted at room temperature (∼298 K). Multiple scans were collected under steady state (when the spectra stopped changing as function of time), and the scans were aligned and merged using the reference foil spectra collected simultaneously. All of the data was processed with ATHENA and ARTEMIS software, with the scattering paths calculated by FEFF 6 codes.^35^ The spectra pre-edge background was removed by fitting a linear function, followed by normalizing to the edge jump. The ΔXANES spectrum were analyzed by subtracting a standard spectrum (metallic Pd-SiO_2_ under He) from the spectra of interest. To obtain EXAFS spectra, the atomic background was subtracted with AUTOBK codes in ATHENA. The EXAFS spectra were further fitted in *R*-space with a *k* weighting of 2 with the inner shell potential correction term Δ*E*_0_ constant for the same catalyst. The electron reduction factor (S_0_^2^) is obtained by fitting the Pd or Au foil spectra collected during experiments. The FEFF 6 input files of the Au and Pd bulk metal crystallographic information files were used to calculate the metal–metal scattering paths. The spectra of Pd-SiO_2_ collected under reaction conditions are fitted with paths including the first three shells with multiple scattering and first shell only. For the fits that include more than one shell, the FT *k* range is chosen to be 3.0–10.9 Å^−1^ and *R* space fitting range is selected as 1.4–4.8 Å. When fitting only one shell, the *k* range is the same, whereas the *R* space range was modified to be 1.4–3.0 Å. The Δ*E*_0_ and S_0_^2^ are 2.9 eV and 0.8 for the *in situ* measurements. The Δ*E*_0_ and S_0_^2^ are 2.9 and 0.74 for the reduced Pd-SiO_2_ measurements, since this was measured during a separate beamtime.

Due to the limited *k* space available, the constraints listed in Tabel S2 is imposed while fitting the Pd K edge EXAFS with paths of the first three shell. Similarly, Table S3 presents fitting constraints imposed while fitting the Au L edge EXAFS.

### 2.6. Elucidating adsorbed species identity through temperature programmed oxidation and attenuated total reflection infrared spectroscopy

Temperature programmed oxidation (TPO) of the spent catalyst before and after exposure to CO_2_ was performed in a commercial device (Micromeritics, Autochem III), fitted with a mass spectrometer (Pfeifer, Thermostar Gas Analysis System) at the outlet to determine the presence of CO_2_-derived adsorbates on the catalyst surface. Catalysts were obtained after exposure to steady-state reaction conditions for 24 h (200 kPa H_2_, 60 kPa O_2_, 0 or 1000 kPa CO_2_, 278 K). The wet catalysts obtained post reaction were vacuum dried at ambient temperature (298 K) for 24 h, before loading 100–500 mg into a U-tube with quartz wool into the temperature programmed device. Prior to oxidation, the thermal conductivity detector (TCD) and mass spectrometer (MS) signal was ensured to be stable under He flow at 298 K. A gas mixture containing 5 cm^3^ min^−1^ of 5% O_2_ balance N_2_ (Airgas, UHP) and 15 cm^3^ min^−1^ of He was flown over the catalyst and passed through a moisture trap before flowing into the TCD and MS. Samples were heated to 873 K at 10 K min^−1^ under gas flow while monitoring the TCD and MS signals and active gas flowrates.

*Operando* attenuated total reflectance infrared (ATR-IR) spectroscopy was employed to determine the identity and evolution of CO_2_-derived organic species under O_2_ reduction reaction conditions. First, ∼40 mg of high weight loading Pd (10 wt%) and Au (2 wt%) catalysts supported on SiO_2_ were dispersed in ∼1 cm^3^ acetone (≥99.5% ACS grade, VWR) and sonicated in a water bath for 30 min. The catalyst slurry was then drip coated onto a polished ZnSe cylindrical internal reflection element (IRE; International Crystal Laboratories) and air dried. The catalyst-coated IRE was loaded into an ATR flow cell (Axiom, TNL-120) and mounted onto a Fourier-transform infrared spectrometer (Bruker, Vertex 70). The catalyst was thermally treated first under 20 kPa O_2_ at 573 K for 1 h, purged with Ar at 573 K for 30 min, and reduced under 20 kPa H_2_ at 573 K for 1 h, before cooling to 303 K under Ar flow to burn off any acetone and other impurities on the catalyst and reduce the surface prior to exposure to reaction conditions.

An HPLC pump (SSI, LS class) was used to introduce gas-saturated DI water solutions at 1 cm^3^ min^−1^ to the ATR-IR flow cell. A back pressure regulator was set up downstream of the cell to pressurize the cell with gas-saturated liquid to mimic kinetic measurements made in the trickle bed reactor. Infrared spectra (128 scans, 4 cm^−1^ resolution) were obtained continuously during liquid flow and background spectra were obtained in Ar-sparged H_2_O. Samples were collected periodically from the outlet of the cell and titrated with the colorimetric indicator outlined in Section 2.3 to determine H_2_O_2_ concentrations under different reaction conditions.

### 2.7. Determining catalyst potential from open circuit potential measurements

*In situ* open-circuit potentiometry (OCP) or catalyst potential (*E*_cat_) measurements were carried out on polycrystalline Pd (Sigma-Aldrich, 0.05 mm thickness, 99.9%) and Au (Goodfellow, 99.999%) foils in a modified Parr Reactor (250 cm^3^, Parr Instruments) to accommodate a pH probe, electrode wires, pressure gauge, gas inlet, and vent as shown in Fig. S8. All experiments were carried out in 0.1 M sodium perchlorate aqueous solutions (250 cm^3^, NaClO_4_, Sigma-Aldrich, ACS reagent, ≥98%) that were initially sparged with O_2_ and Ar and stirred at 1500 rpm using a magnetic stir bar unless otherwise specified. The three-electrode assembly consisted of either Pd or Au foils as the working as well as counter electrodes, and a leak-free silver–silver chloride reference electrode (Ag/AgCl; Innovative Instruments, 3.4 M KCl leak-free 1.6 mm diameter). The reference electrode was calibrated to a second Ag/AgCl electrode at ambient conditions before and after measurements and any drift was corrected in the *E*_cat_ calculations (*E*_ref *vs.* Ag/AgCl_, eqn (2)). Prior to *E*_cat_ measurements, the catalyst foils were cleaned *in situ* with cyclic voltammetry (10–20 cycles, −1 to 1 V for Pd and −1 to 1.5 V *vs.* Ag/AgCl for Au, 100 mV s^−1^) to remove any contaminants from the surface (Fig. S9).

All *E*_cat_ measurements were conducted in a batch operation where the Parr reactor was sparged with desired flowrates and partial pressures of reactant gases (25% H_2_/N_2_, 5% O_2_/N_2_, and 5% O_2_/CO_2_) using Bronkhorst MFCs at ambient temperature (298 K) and pressure until OCP values were stable. The reactor was then pressurized to the desired pressure (to match trickle bed reactor measurements) with reactant gases. Reported OCP or *E*_cat_ values were measured once the reactor maintained stable pressures after gases were shut off. Representative *E*_cat_ profile with time is shown in Fig. S10. Measured *E*_cat_ values were converted to the Ag/AgCl scale and reversible hydrogen electrode (RHE) scale with a unit activity of hydronium ([H_3_O^+^] = 1), 1 bar H_2_, and accounting for the measured pH following eqn (2).^36–38^

Measured *E*_cat_ values were converted to the RHE scale following,2
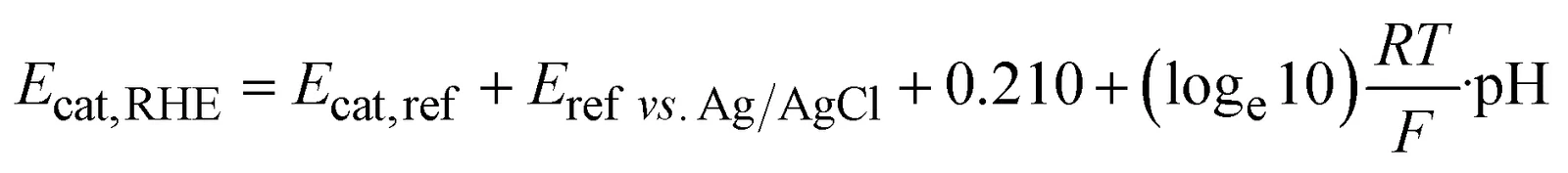


## 3. Results and discussion

### 3.1. Effect of CO_2_ on H_2_O_2_ and H_2_O product formation rates and selectivity

Formation rates of H_2_O_2_ and H_2_O from reactions of O_2_ and H_2_ were measured over SiO_2_-supported Pd and Au catalysts with mixtures of N_2_ and CO_2_ as diluent gases. The nanoparticle catalysts were synthesized by strong electrostatic adsorption following established procedures.^15,24–26^ Steady-state reaction rates obtained in a fixed bed reactor reflect intrinsic reaction kinetics without artifacts introduced by external or internal transport limitations.^27–30^ Following catalyst activation and stabilization of rates *in situ* (Fig. S6), fluid residence times were controlled (Fig. S7) to isolate primary formation pathways for H_2_O_2_ and H_2_O (pathways 1 and 2, Scheme 1) and suppress contributions from secondary H_2_O formation routes associated with H_2_O_2_ decomposition and hydrogenation (pathways 3 and 4, Scheme 1) at all conditions.

**Scheme 1:**
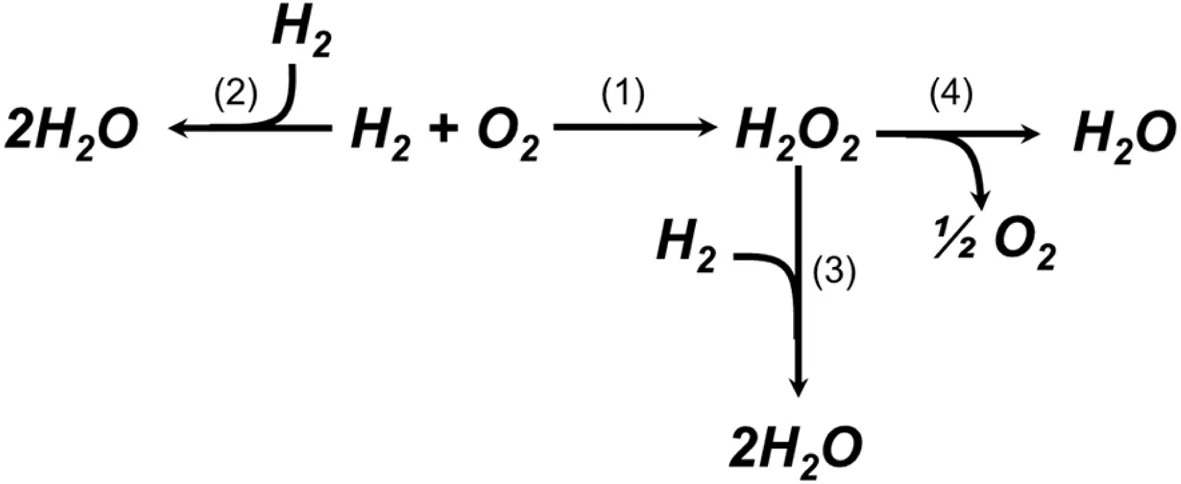
Reactions among H_2_- and O_2_-derived intermediates over metal nanoparticles lead to primary pathways that form (1) H_2_O_2_ and (2) H_2_O and secondary pathways that (3) hydrogenate H_2_O_2_ and (4) disproportionate H_2_O_2_.

Fig. 1 presents product formation rates for both H_2_O_2_ and H_2_O as a function of time on stream following catalyst activation and shows that rates depend sensitively upon the identity of the diluent gas when all other factors remain constant (200 kPa H_2_, 60 kPa O_2_, 278 K). H_2_O_2_ formation rates increase by 65% on Pd catalysts and 30% on Au catalysts when 1150 kPa CO_2_ replaces 1150 kPa N_2_ at these conditions and reaches steady-state values in ∼60 min. H_2_O formation rates on both Pd and Au catalysts exhibit a prolonged induction period of ∼600 min when CO_2_ replaces N_2_ at the inlet, ultimately reaching lower steady-state rates under CO_2_. The contrasting responses of H_2_O_2_ and H_2_O formation rates to CO_2_ indicate that distinct mechanistic factors influence the pathways to form each product. The combined changes in H_2_O_2_ and H_2_O formation rates result in a substantial increase in steady-state H_2_O_2_ selectivity on Pd from 55% to 90%, whereas H_2_O_2_ selectivities on Au increase only slightly from 85% to 93% following the introduction of CO_2_ (Table 1). The increase in H_2_O_2_ formation rates occurs reversibly on both Pd and Au catalysts; N_2_ (1150 kPa) replaces CO_2_ (800 minutes, Fig. 1), and H_2_O_2_ formation rates return to values comparable to rates measured before contact with CO_2_. In contrast, adding CO_2_ irreversibly suppresses H_2_O formation, with the effect being more pronounced on Pd. This irreversible suppression of H_2_O formation leads to a persistent increase in H_2_O_2_ selectivity on Pd. To elucidate the origin of these differences in formation rates and selectivities upon addition of CO_2_, we examine possible changes to the reaction mechanism, surface coverages, and site requirements, as well as catalyst-dependent structural responses in Pd and Au systems. The discussion hereafter primarily focuses on H_2_O_2_ formation rates with limited discussion of H_2_O formation rate, which fall near or below the limit of quantification at these conditions.

**Fig. 1:**
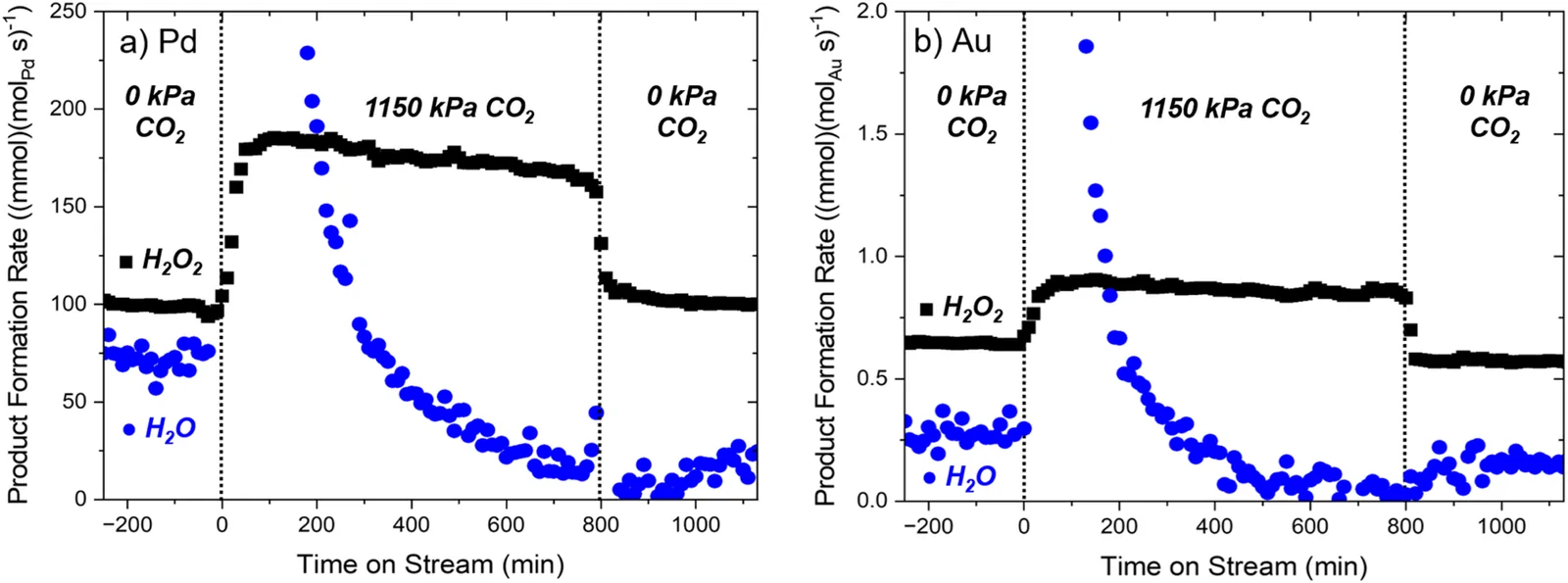
Time on stream profiles for H_2_O_2_ formation rates (black ■) and H_2_O formation rates (blue 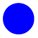) on (a) Pd-SiO_2_ and (b) Au-SiO_2_ with 0 and 1150 kPa CO_2_ after catalyst break-in (200 kPa H_2_, 60 kPa O_2_, 278 K).

**Table 1:** Steady-state H_2_O_2_ selectivities on Pd-SiO_2_ and Au-SiO_2_ before, during, and after CO_2_ exposure (200 kPa H_2_, 60 kPa O_2_, 1740 kPa N_2_, or 1150 kPa CO_2_ + 590 kPa N_2_, 278 K)

Catalyst	H_2_O_2_ selectivity (%)		
	Before CO_2_ (0 kPa CO_2_)	During CO_2_ (1150 kPa CO_2_)	After CO_2_ (0 kPa CO_2_)
Pd-SiO_2_	55 ± 4	90 ± 8	85 ± 10
Au-SiO_2_	85 ± 7	93 ± 10	87 ± 5

### 3.2. CO_2_ alters hydrogen activation and coverages on catalyst surfaces

The local proton concentration (*i.e.*, [H^+^] or pH) strongly influences H_2_O_2_ formation rates and selectivities, and acidic environments (high [H^+^]) favor the two-electron O_2_ reduction pathway to form H_2_O_2_.^39,40^Fig. 2a presents steady-state rate measurements that show the promotional effects of CO_2_ on H_2_O_2_ formation under H_2_-rich conditions for both Pd and Au catalysts emerge most clearly above a critical CO_2_ pressure of ∼600 kPa, where H_2_O_2_ rates exhibit a sublinear dependence on CO_2_ pressure. The addition of CO_2_ (1150 kPa CO_2_) forms carbonic acid (p*K*_a_ = 6.35),^41^ lowers the solution pH, and increases H_2_O_2_ formation rates to an extent comparable to direct acidification with sulfuric acid (H_2_SO_4_; comparison made in semi-batch experiments, Fig. S11 and S12). Consistent with this behavior, steady-state H_2_O_2_ formation rates on both Pd and Au catalysts from trickle bed reactor measurements show a strong dependence on [H^+^]^42^ and pH as a result of CO_2_ acidification (Fig. S13). However, apparent rate constants for H_2_O_2_ formation and decomposition extracted from H_2_O_2_ concentration-time profiles from semi-batch experiments differ markedly between CO_2_ and H_2_SO_4_ at comparable pH values (Tables S4 and S5). These differences indicate that CO_2_ introduces additional physical or chemical effects beyond changes in bulk solution pH and suggest that dissolved CO_2_ or CO_2_-derived species modify O_2_ reduction pathways.

**Fig. 2:**
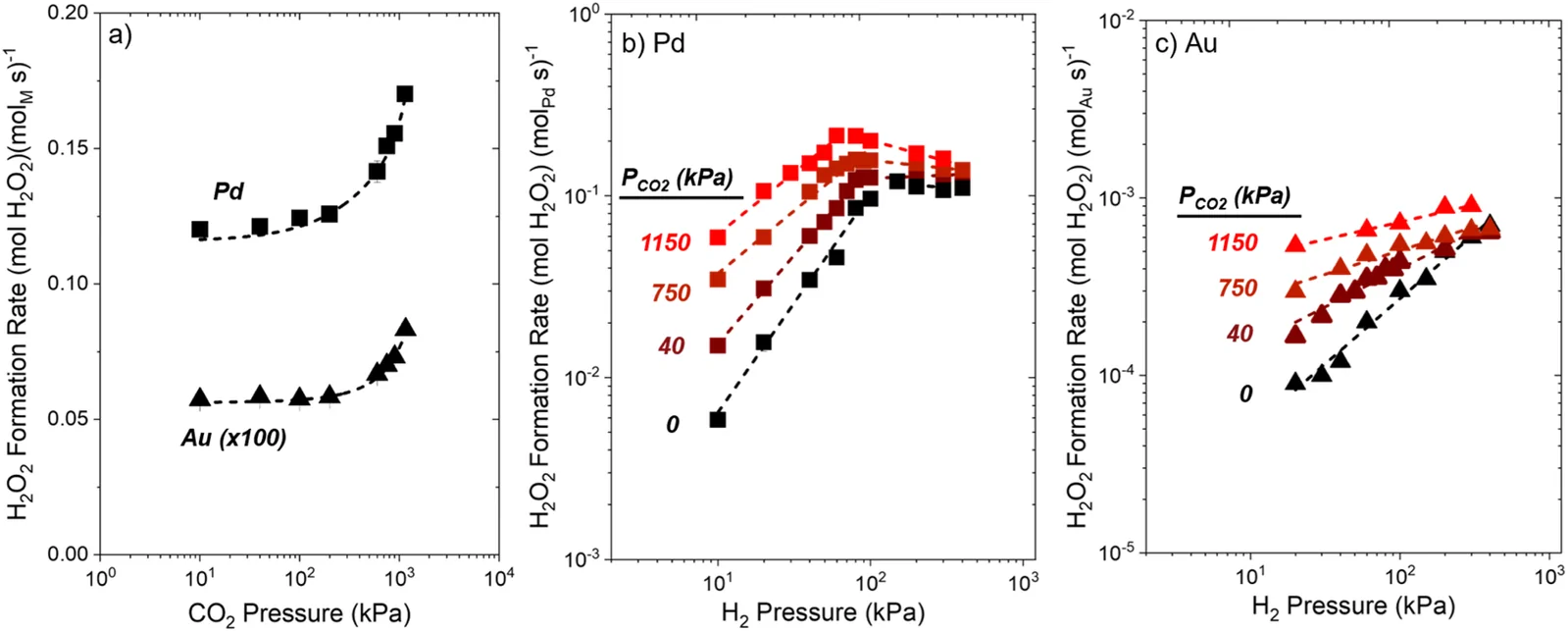
H_2_O_2_ formation rates as a function of (a) CO_2_ pressure on Au-SiO_2_ (▲; rates multiplied by a factor of 100) and Pd-SiO_2_ (■; 200 kPa H_2_, 60 kPa O_2_, 278 K), (b) H_2_ pressure on Pd-SiO_2_, and (c) Au-SiO_2_ at different CO_2_ pressures (60 kPa O_2_, 0–1150 kPa CO_2_, 278 K).

In the absence of CO_2_, Pd and Au nanoparticles catalyze O_2_ reduction with H_2_ to form H_2_O_2_ and H_2_O through pathways described previously (Schemes 2 and S1).^15,24^ This mechanism involves non-competitive adsorption O_2_ to yield oxygen-derived species (*e.g.*, 
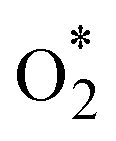
, O*) and H_2_ to form chemisorbed hydrogen (H^#^) at distinct sites. Kinetically relevant H_2_ dissociation occurs with the assistance of solution-phase water to produce chemisorbed H^#^, hydronium ions (H_3_O^+^), and electrons (step 1). Concurrent quasi-equilibrated adsorption of O_2_ yields 
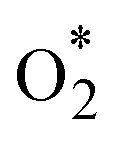
 (step 2), which undergoes irreversible proton–electron transfer *via* solution-phase H_3_O^+^ to form OOH* (step 3). A second proton–electron transfer reduces OOH* to create either H_2_O_2_ when the O–O bond remains intact (step 4) or H_2_O and O* when the O–O bond cleaves (step 5). Both H_2_O_2_ and H_2_O desorb reversibly. While these steps occur on both Pd and Au catalysts, each catalyst differs in the kinetic relevance of hydrogen activation and steady-state coverages of reactive intermediates (*vide infra*, detailed mechanism and rate expression derivations in Section S3).

**Scheme 2:**
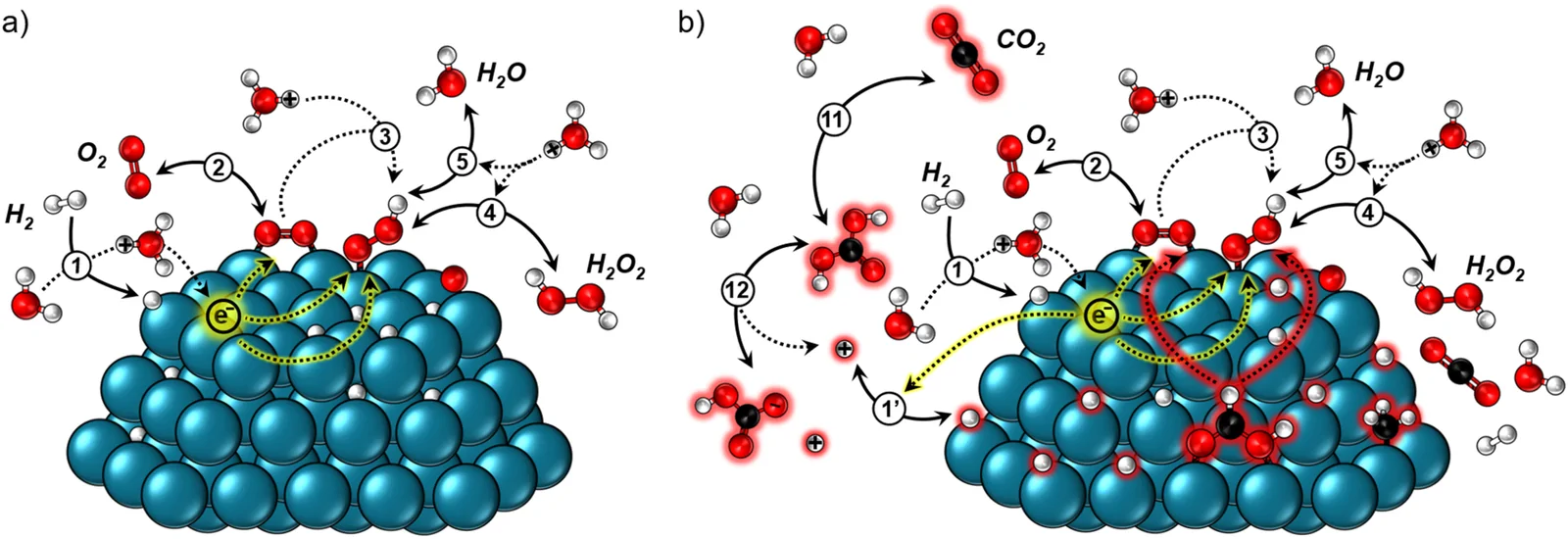
Simplified elementary steps for primary H_2_O_2_ and H_2_O formation by catalytic O_2_ reduction on Pd catalysts in the (a) absence of CO_2_, and (b) proposed changes to the mechanism in the presence of CO_2_. Dashed lines represent proton–electron transfer steps. The symbol → denotes irreversible reactions and ↔ signifies reversible or quasi-equilibrated reactions. Schemes S1 and S2 list the elementary steps involved in O_2_ reduction outlined in this scheme.

Steady-state rates of H_2_O_2_ (and H_2_O) formation (*r*_H_2_O_2__) depend also on H_2_ and O_2_ pressures, which report on changes in the coverages of reactive species involved in kinetically relevant H_2_ activation and O_2_ reduction. In the absence of CO_2_, H_2_O_2_ formation rates on Pd increase linearly at low H_2_ pressures (Fig. 2b; 10–100 kPa H_2_, 60 kPa O_2_) and subsequently approach nearly constant values at higher H_2_ pressures (100–400 kPa H_2_, 60 kPa O_2_). These trends reflect changes in the coverages of chemisorbed hydrogen atoms that initially increase before saturating at elevated H_2_ pressures, and H_2_O_2_ formation rates that cohere to rate expressions that describe irreversible H_2_ activation at low hydrogen coverages (eqn (3)) and quasi-equilibrated H_2_ activation at high hydrogen coverages (eqn (4)). The change in coverages and governing rate expressions in the absence of CO_2_ also corresponds to the discontinuity in H_2_O rates and selectivities (Fig. S14) and the associated conversion of metallic Pd nanoparticles into β-PdH_*x*_ structures, as demonstrated by *operando* X-ray absorption spectroscopy (*vide infra*).^15^3
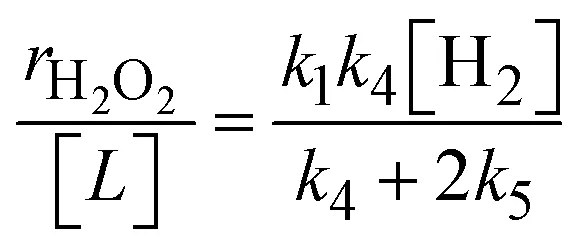
4
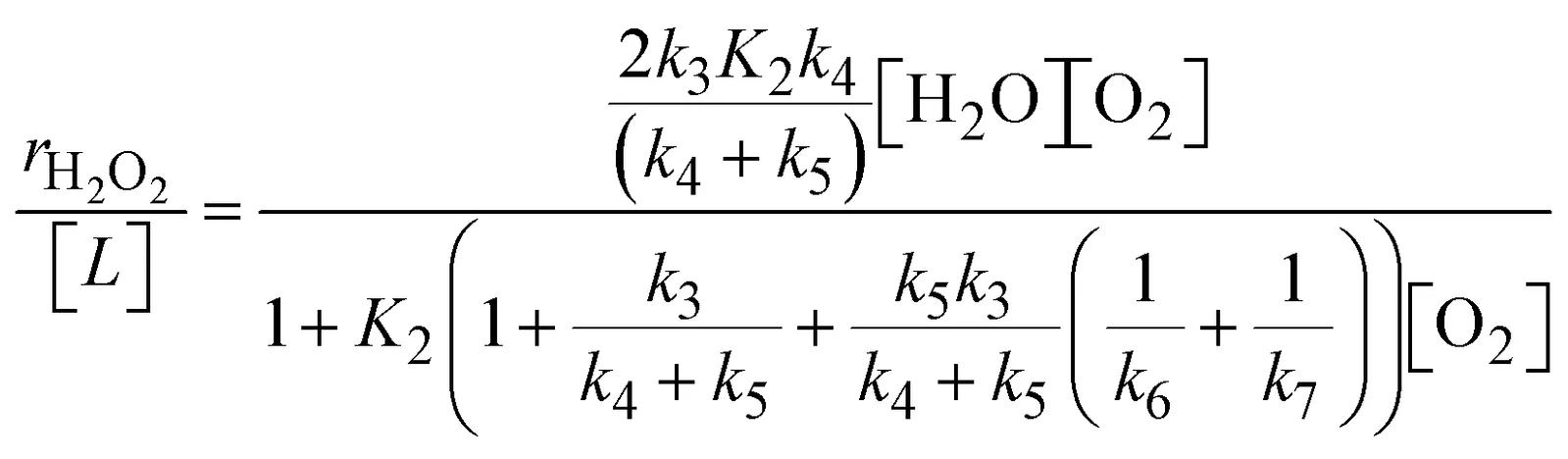


The addition of CO_2_ (40–1150 kPa CO_2_) alters the Pd nanoparticles response to H_2_ pressure and increases H_2_O_2_ formation rates at all H_2_ pressures. For example, H_2_O_2_ rates increase by 19% under H_2_-rich conditions (400 kPa H_2_, 60 kPa O_2_) and 76% under O_2_-rich conditions (40 kPa H_2_, 60 kPa O_2_) and show similar functional dependence on H_2_ pressure with the addition of 40 kPa CO_2_. Increasing CO_2_ pressure above 40 kPa continues to increase H_2_O_2_ rates but the pressure dependences of *r*_H_2_O_2__ on H_2_ pressure differ considerably. In the kinetic regime where H^#^ does not saturate the Pd surface (10–80 kPa H_2_, 60 kPa O_2_), H_2_O_2_ formation rates exhibit a sublinear dependence on H_2_ pressure, which decreases marginally with increasing CO_2_ pressure (*e.g.*, *r*_H_2_O_2__∼[H_2_]^0.8^ at 750 kPa CO_2_; *r*_H_2_O_2__∼[H_2_]^0.7^ at 1150 kPa CO_2_). Weaker H_2_ dependences suggest CO_2_ increases H^#^ coverages by increasing [H^+^] through CO_2_ dissolution in water at high pressures (forming carbonic acid and its derivatives; Scheme 2b, Section S2), where the solution-phase H^+^ subsequently forms neutral chemisorbed hydrogen *via* a Volmer-type step (Step 1’, Scheme 2b).^43,44^ Similarly, H_2_O_2_ rates increase and rate orders in H_2_ decrease upon CO_2_ introduction under conditions where H^#^ covers Pd surfaces (>100 kPa H_2_, 60 kPa O_2_) and give a weakly negative dependence of H_2_O_2_ formation rates on H_2_ pressure (*r*_H_2_O_2__∼[H_2_]^−0.1^ at 750 kPa CO_2_; *r*_H_2_O_2__∼[H_2_]^−0.2^ at 1150 kPa CO_2_). Additionally, H_2_O_2_ rates converge to similar values at sufficiently high H_2_ pressures (>300 kPa H_2_) across all CO_2_ pressures. This behavior shows the promotional effects of CO_2_ diminish at the greatest H_2_ pressures and implicates differences in the Pd surface environment, such as variations in the coverages of O_2_- and CO_2_-derived surface intermediates, shifts in the kinetic relevance and nature of H_2_ oxidation and OOH* formation, or a combination of these factors.

The addition of CO_2_ increases hydrogen coverages on Au nanoparticles by increasing [H^+^] in solution in a similar manner to Pd. In the absence of CO_2_, H_2_O_2_ formation rates on Au nanoparticles increase linearly with H_2_ pressures across the entire range of pressures examined (10–400 kPa H_2_, 60 kPa O_2_). This behavior coheres with a single kinetic regime where H^#^ coverage on Au nanoparticles increases with increasing H_2_ pressures but Au sites do not saturate with H^#^ under these conditions, unlike Pd. Hence, H_2_O_2_ formation rates on Au agree with the rate expression that describes irreversible H_2_ activation and low H^#^ coverages at all conditions (*r*_H_2_O_2__∼[H_2_]^1^, eqn (3)).^29^ The addition of CO_2_ increases H_2_O_2_ rates on Au at all H_2_ pressures and decreases the functional dependence on H_2_ pressures (*r*_H_2_O_2__∼[H_2_]^0.5^, *r*_H_2_O_2__∼[H_2_]^0.3^, and *r*_H_2_O_2__∼[H_2_]^0.2^ at 40, 750, and 1150 kPa CO_2_ respectively). These kinetic changes with CO_2_ addition suggest CO_2_ increases hydrogen coverages through analogous pathways to Pd, resulting in Au surfaces approaching H^#^ saturation at high CO_2_ and H_2_ pressures (Scheme S3).

Comparisons of HD formation rates with total peroxide (*i.e.*, H_2_O_2_, D_2_O_2_, HDO_2_) formation rates reveal differences in hydrogen (or deuterium) surface coverages and additionally probe changes to the reversibility of hydrogen activation during O_2_ reduction in the presence of CO_2_.^45,46^Fig. 3a and b show total peroxide and HD formation rates as functions of combined hydrogen and deuterium pressures ([H_2_] + [D_2_]) on Pd catalysts in the absence and presence of CO_2,_ respectively (10–200 kPa H_2_, 10–200 kPa D_2_, H_2_ : D_2_ = 1, 60 kPa O_2_, 0 and 1150 kPa CO_2_). Formation rates for peroxides appear as functions of the combined pressures of H_2_ and D_2_ consistent with those observed for H_2_ alone (Fig. 2b). In the absence of CO_2_, HD formation rates remain lower than total peroxide formation rates (*r*_HD_/*r*_H_2_O_2__ = 0.1; 40 kPa (H_2_ + D_2_)) in the kinetic regime where Pd surfaces have low steady-state H/D coverages, which suggests H_2_ activates largely irreversibly and cohere with previously established mechanism and rate expression (eqn (3)).^15^ On the other hand, HD rates on Pd increase with ([H_2_] + [D_2_]) and exceed total peroxide rates at higher pressures (*r*_HD_/*r*_H_2_O_2__ = 10; 600 kPa (H_2_ + D_2_))_._ These observations agree with H/D saturation on Pd surface sites and an increase in the reversibility of H_2_ activation that approaches quasi-equilibrium at higher pressures (eqn (4)). In the presence of CO_2_, HD formation rates on Pd become closer in magnitude to peroxide formation rates in both kinetic regimes (*r*_HD_/*r*_H_2_O_2__ = 0.2 at 40 kPa (H_2_ + D_2_); *r*_HD_/*r*_H_2_O_2__ = 3.9 at 600 kPa (H_2_ + D_2_)). This result reveals that hydrogen (or deuterium) activation in the presence of CO_2_ on Pd nanoparticles proceeds with greater reversibility at lower pressures and becomes increasingly reversible (but never quasi-equilibrated) at higher pressures as evidenced by the diminishing difference between the two rates (Fig. 3b).

**Fig. 3:**
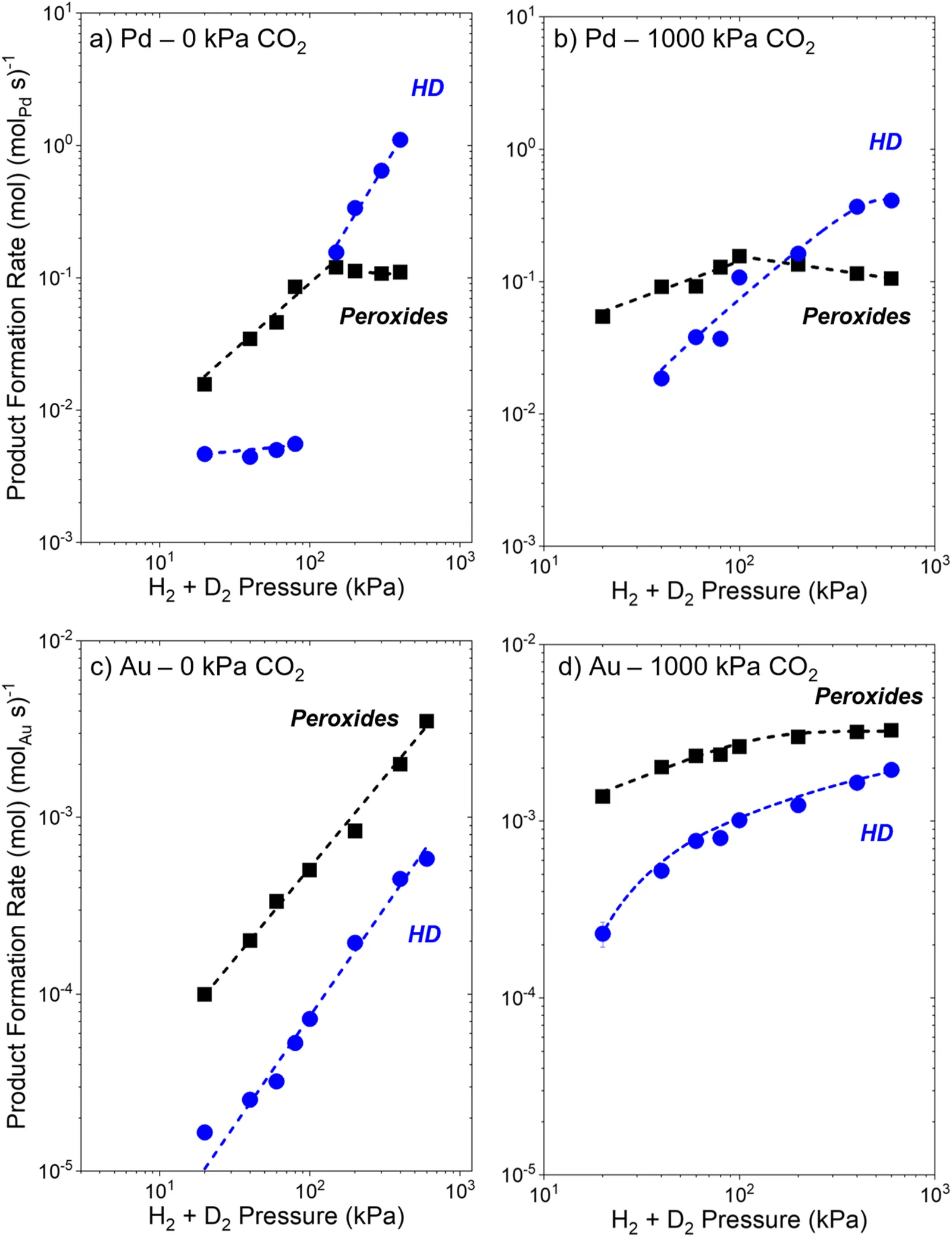
Formation rates of peroxide (*i.e.*, sum of H_2_O_2_, D_2_O_2_, and HDO_2_, black ■) and HD (blue 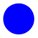) as functions of the combined hydrogen and deuterium pressure (10–200 kPa H_2_, 10–200 kPa D_2_, H_2_ : D_2_ = 1, 60 kPa O_2_, 286 K) on Pd-SiO_2_ at (a) 0 kPa CO_2_ and (b) 1000 kPa CO_2_ and Au at (c) 0 kPa CO_2_ and (d) 1000 kPa CO_2_.

Similar comparisons between HD and total peroxide formation rates on Au nanoparticles reveal CO_2_ and CO_2_-derived species increase hydrogen (or deuterium) surface coverages as well as the reversibility of H_2_ (or D_2_) activation. Fig. 3c shows both total peroxide and HD formation rates increase proportionally with the combined pressure of H_2_ and D_2_ (10–200 kPa H_2_, 10–200 kPa D_2_, H_2_ : D_2_ = 1, 60 kPa O_2_) in the absence of CO_2_, consistent with increasing H/D coverages. HD formation rates remain lower than total peroxide rates by more than 10-fold across the entire range of pressures examined, indicating irreversible H_2_ (or D_2_) activation on Au nanoparticles under these conditions. In the presence of CO_2_, both the total peroxide and HD formation rates increase on Au while their functional dependence on ([H_2_] + [D_2_]) pressures decrease (Fig. 3d). Furthermore, HD formation rates become closer in magnitude to the total peroxide rates at all pressures when CO_2_ is present (*r*_HD_/*r*_H_2_O_2__ = 0.2–0.5), confirming that CO_2_ addition to thermochemical O_2_ reduction reactions not only increases the surface hydrogen (or deuterium) coverage but also enhances the reversibility of H_2_ (or D_2_) activation on Au nanoparticles similar to Pd.

### 3.3. CO_2_ introduces new oxygen reduction pathways *via* redox mediation

Peroxide and HD formation rate dependencies on reactant pressures indicate that CO_2_ and CO_2_-derived species increase hydrogen coverage on Pd and Au nanoparticle surfaces and alter the reversibility of H_2_ activation. These changes could, in turn, modify the kinetic relevance of steps involved in O_2_ reduction by H_2_ to form H_2_O_2_ by introducing new reaction pathways. In the absence of CO_2_, H_2_O_2_ formation rates on both Pd and Au nanoparticles remain greater in H_2_ than D_2_ at all H_2_ (or D_2_) pressures (Fig. 4 and S15; 20–200 kPa H_2_ or D_2_, 60 kPa O_2_, 286 K), consistent with a normal primary kinetic isotope effect (*r*_H_/*r*_D_ > 1) for H_2_O_2_ formation. Additionally, rates of H_2_O_2_ formation ((*k*_H_2_O_/*k*_D_2_O_)_Pd_ = 1.1 ± 0.1, (*k*_H_2_O_/*k*_D_2_O_)_Au_ = 1.1 ± 0.1) and decomposition ((*k*_H_2_O_/*k*_D_2_O_)_Pd_ = 1.2 ± 0.2, (*k*_H_2_O_/*k*_D_2_O_)_Au_ = 1.0 ± 0.1) on Pd and Au do not respond to substituting the solvent water with deuterated water in the absence of CO_2_ (Table 2 and Fig. S16; 5 kPa H_2_, 5 kPa O_2_, 91 kPa N_2_, 298 K). Taken together, these isotopic substitution results confirm that reactions involving H_2_ activation and subsequent oxidation proceed with kinetic relevance for O_2_ reduction to H_2_O_2_ on Pd and Au catalysts; however, proton transfer steps mediated by water appear to remain comparatively facile in the absence of CO_2_.^15,29^

**Fig. 4:**
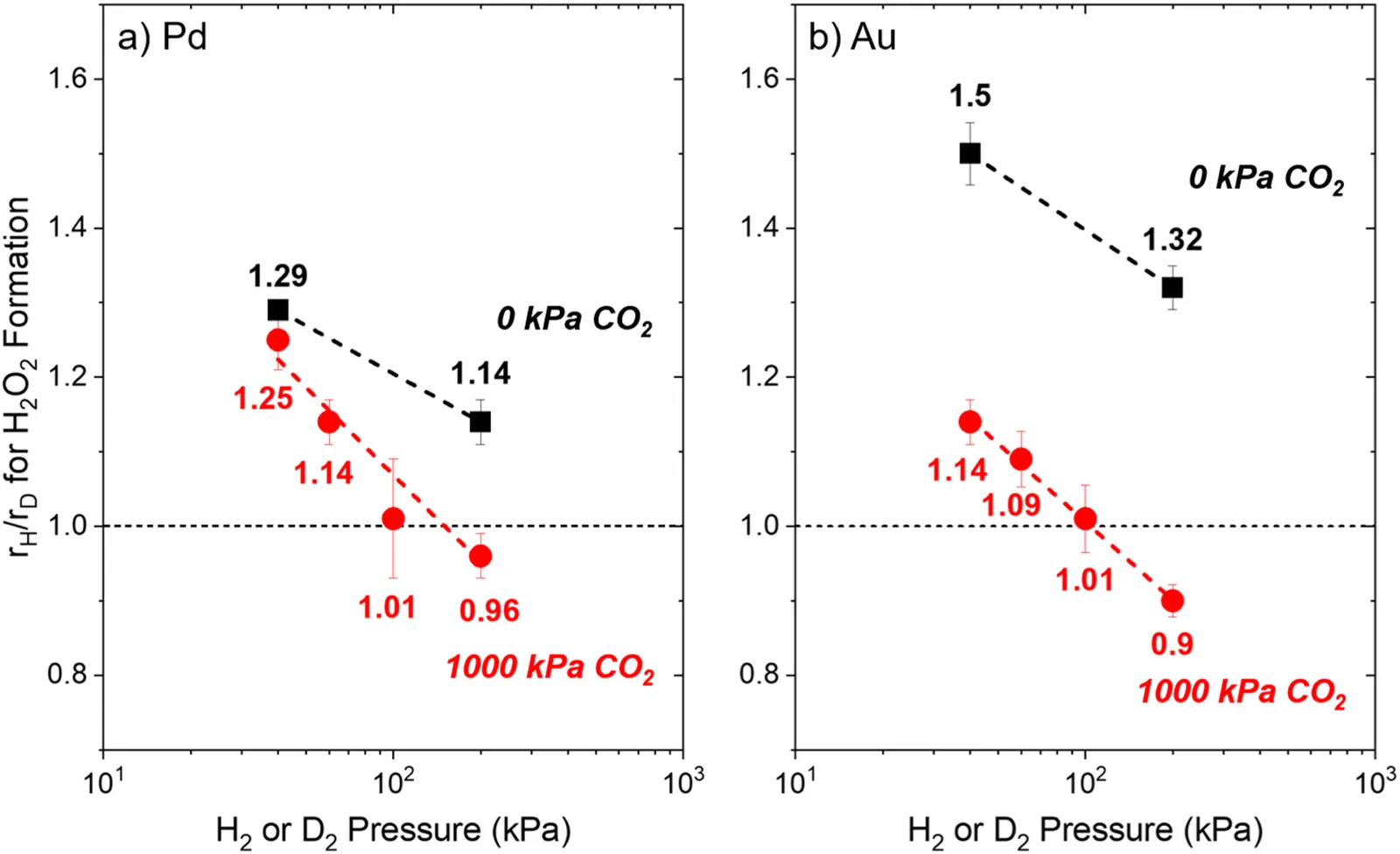
Ratio of the rates of hydrogen peroxide formation from H_2_ or D_2_ reactants (*r*_H_/*r*_D_) on (a) Pd-SiO_2_ and (b) Au-SiO_2_ as functions of H_2_ or D_2_ pressures (20–200 kPa H_2_ or D_2_, 60 kPa O_2_, 286 K) with 0 kPa CO_2_ (black ■) and 1000 kPa CO_2_ (red 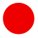).

**Table 2:** Effects of H_2_O-D_2_O isotopic substitution on rate constants for H_2_O_2_ formation and decomposition on Pd and Au catalysts in the absence and presence of CO_2_ (5 kPa H_2_, 5 kPa O_2_, 0 or 91 kPa CO_2_, 80 cm^3^ H_2_O or D_2_O, 298 K)

*k*_H_2_O_/*k*_D_2_O_	Pd		Au	
	0 kPa CO_2_	91 kPa CO_2_	0 kPa CO_2_	91 kPa CO_2_
H_2_ + O_2_ → H_2_O_2_	1.1 ± 0.1	2.0 ± 0.4	1.1 ± 0.1	1.0 ± 0.2
H_2_O_2_ + H_2_ → 2H_2_O	1.2 ± 0.2	1.6 ± 0.5	1.0 ± 0.1	1.2 ± 0.3

The addition of CO_2_ causes H_2_O_2_ formation rates to remain greater in H_2_ than D_2_ (*r*_H_/*r*_D_ > 1) at low H_2_ or D_2_ pressures (Fig. 4 and S17; <80 kPa H_2_ or D_2_, 60 kPa O_2_, 1000 kPa CO_2_, 286 K) on both Pd and Au nanoparticles, suggesting CO_2_ does not induce significant changes to the kinetic relevance of steps involved in H_2_O_2_ formation at these conditions. However, H_2_O_2_ formation rates remain similar and subsequently become lower in H_2_ than in D_2_ (*r*_H_/*r*_D_ ≤ 1) with increasing H_2_ or D_2_ pressures (>80 kPa H_2_ or D_2_) in the presence of CO_2_. The decrease in *r*_H_/*r*_D_ values below unity in the presence of CO_2_ for both Pd and Au implies a transition from a normal kinetic isotope effect to a regime influenced by equilibrium isotope effects. Notably, introducing CO_2_ leads to a pronounced solvent kinetic isotope effect on Pd for both H_2_O_2_ formation ((*k*_H_2_O_/*k*_D_2_O_)_Pd_ = 2.0 ± 0.4) and decomposition ((*k*_H_2_O_/*k*_D_2_O_)_Pd_ = 1.6 ± 0.5) (Fig. S18; 5 kPa H_2_, 5 kPa O_2_, 91 kPa CO_2_, 298 K). In contrast, rates of H_2_O_2_ formation ((*k*_H_2_O_/*k*_D_2_O_)_Au_ = 1.0 ± 0.2) and decomposition ((*k*_H_2_O_/*k*_D_2_O_)_Au_ = 1.2 ± 0.3) on Au remain insensitive to isotopic substitution of the solvent water in the presence of CO_2_. These comparisons give evidence that CO_2_ introduces new reaction pathways on Pd in which proton transfer from water becomes kinetically relevant (Scheme 2b).

Mixed potential theory provides a powerful and unique framework for interpreting catalytic reactions that proceed through coupled oxidation-reduction pathways, allowing electrochemical descriptors to be linked directly to underlying thermochemical steps.^36–38,47,48^ In thermochemical systems involving oxidation-reduction steps like the coupled H_2_ oxidation-O_2_ reduction steps that form H_2_O_2_ and H_2_O, the open-circuit potential (OCP) or catalyst potential (*E*_cat_) reflects the dynamic balance between the simultaneously occurring oxidation and reduction half-reactions on the catalyst surface. Since the relative rates and energetics of these half-reactions govern values of *E*_cat_, *in situ* measurements of *E*_cat_ can detect shifts in reaction pathways, changes in adsorbate coverages, and the formation of surface redox mediators.^49^Fig. 5 shows *E*_cat_ measurements from Pd and Au under H_2_O_2_ reaction conditions as functions of H_2_ pressures in the presence of different quantities of CO_2_ that suggest the appearance of new reaction pathways and CO_2_-derived intermediates upon Pd surface but not on Au surfaces (20–200 kPa H_2_, 60 kPa O_2_, 298 K). Fig. 5a shows *E*_cat_ values of Pd foil decrease logarithmically with H_2_ pressures for any given CO_2_ pressure (0–1110 kPa). The values of *E*_cat_ are reported in comparison to the reversible hydrogen electrode (RHE), which accounts for changes in pH induced by different CO_2_ pressures. These values of *E*_cat_ deviate from the slope for Nernstian HOR and ORR behavior, which demonstrates multiple half-reactions contribute to the measured *E*_cat_ values. The sensitivity of *E*_cat_ to H_2_ pressures confirms that *E*_cat_ reflects a mixed potential determined by reactions among hydrogen, oxygen, water, and potentially carbon dioxide during H_2_O_2_ and H_2_O synthesis (eqn (5) and (6)). Furthermore, the magnitude of *E*_cat_ should not be directly compared with the cathodic potentials required for conventional electrochemical CO_2_ reduction since *E*_cat_ represents a mixed potential under open-circuit reaction conditions; instead, the deviations in *E*_cat_ suggest that CO_2_ participates in additional surface reactions under these thermochemical oxygen reduction reactions. Values of *E*_cat_ obtained from Au across H_2_ pressures (Fig. 5b) exhibit similar behavior.

5 *i*_net_ = ∑*i*_ox_ + ∑*i*_red_ = 0

6 *i*_net_=(*i*_HOR_ + *i*_CO_2_mediator oxidizing_) + (*i*_4e_ − _ORR_ + *i*_2e_ − _ORR_ + *i*_CO_2_reducing_ + *i*_HER_) = 0

**Fig. 5:**
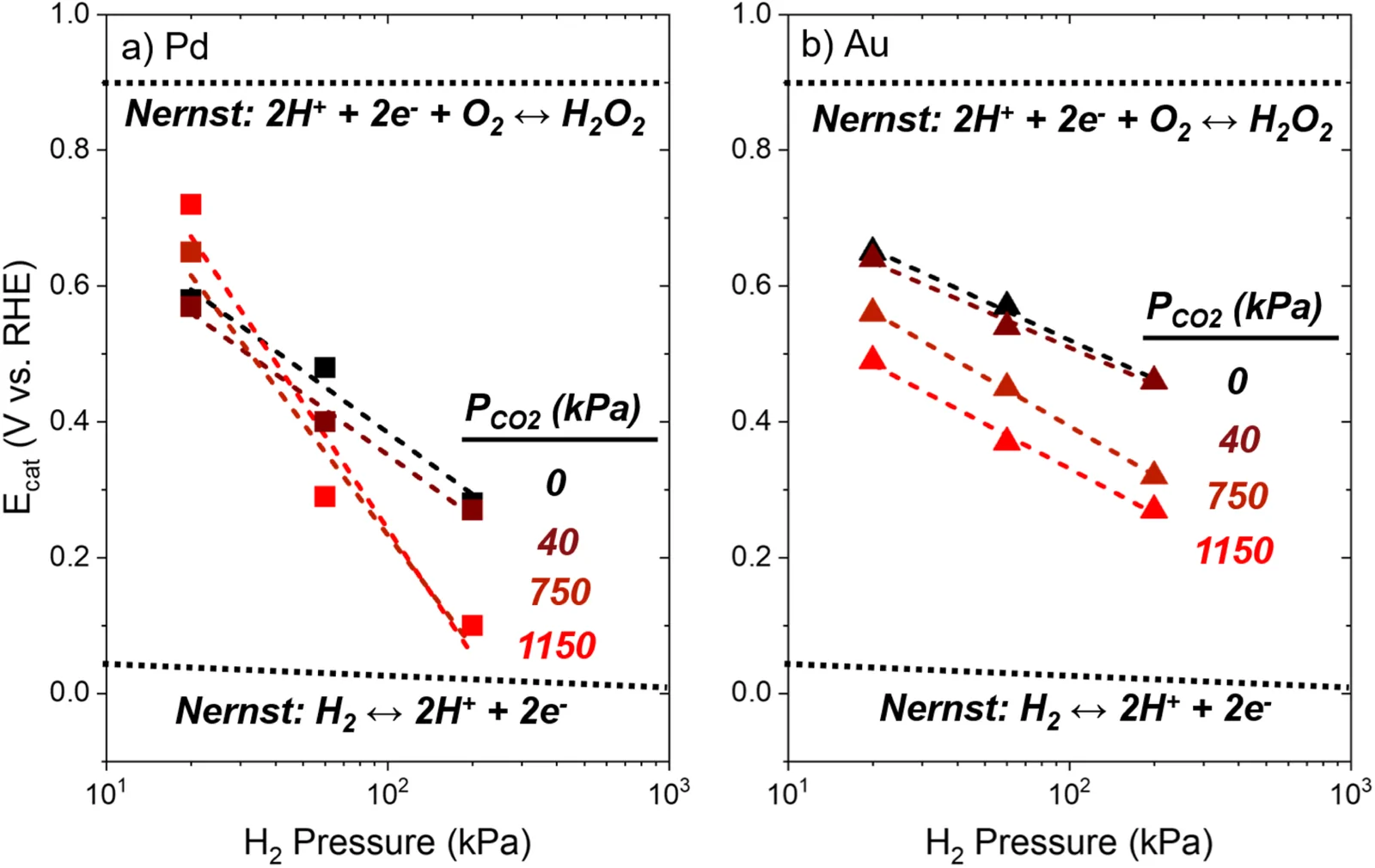
Open-circuit potentiometry measurements of catalyst potentials for (a) Pd (■) and (b) Au (▲) foils as functions of H_2_ pressure (20–200 kPa of H_2_, 60 kPa of O_2_, 298 K) at different CO_2_ pressures in comparison to the Nernstian response for the HOR and ORR paths. Nernstian expressions were calculated using the unit activities of H_3_O^+^ and H_2_O species (Section 2.6).

While *E*_cat_ values for Pd follow the same trend with increasing H_2_ pressures at all CO_2_ pressures, these observations reveal distinct dependence of *E*_cat_ on H_2_ pressure with greater sensitivity at the greatest CO_2_ pressures (Fig. 5a). This trend agrees with the reduced magnitude of the H_2_/D_2_ kinetic isotope effect and equilibrium isotope effect for H_2_ oxidation observed when both CO_2_ and H_2_ pressures remain high (Fig. 4a). The changes in *E*_cat_ values and the slopes (or scaling relationships) with H_2_ and CO_2_ pressures on Pd further imply changes to the kinetic relevance of half-reactions that mediate the overall process for hydrogen oxidation and the emergence of new redox reactions that involve CO_2_-derived surface intermediates. These changes do not appear for Au (Fig. 5b): the sensitivity of *E*_cat_ to changes in the H_2_ pressure remains constant at all CO_2_ pressures while values of *E*_cat_ become more negative with increasing CO_2_ pressures. This suggests only a shift in mixed potential towards HOR and increased hydrogen coverages on Au as described by thermochemical isotopic measurements (Fig. 5b) without changes to the kinetic relevance of half-reactions or the addition of new half-reactions. These differences provide further evidence for a consequential change in surface chemistry upon Pd that does not occur on Au.

Results from kinetic dependencies, isotopic substitution, and catalyst potential measurements suggest CO_2_ influences O_2_ reduction catalysis through mechanisms that extend beyond simple pH effects. On Au, the promotion of H_2_O_2_ formation arises primarily from increased surface hydrogen coverages resulting from increased proton concentration and rates of Volmer reduction of protons in the presence of CO_2_. This subsequently results in H_2_ activation becoming increasingly reversible at high pressures and hydrogen coverages in the presence of CO_2_ (Fig. 3d), making equilibrium isotope effects more prominent. Together, these effects diminish the observed H_2_/D_2_ kinetic isotope effect in the presence of CO_2_ and reflect a transition from kinetically controlled H_2_ activation to a regime governed by high hydrogen coverages and near-equilibrated surface hydrogen species. In summary, these differences indicate that the mechanisms for H_2_ activation and oxidation to form H_2_O_2_ and H_2_O remain similar with and without CO_2_ on Au surfaces, but the addition of CO_2_ and increasing [H^+^] introduces changes in the kinetic relevance of H_2_ activation and surface coverages of intermediates. On Pd surfaces, in addition to the promotional effects outlined for Au, CO_2_ and CO_2_-derived intermediates formed *in situ* act as redox mediators facilitating proton transfer between water and O_2_-derived species to promote selective H_2_O_2_ formation.

### 3.4. Role and identity of CO_2_-derived intermediates formed in situ on modifying Pd surfaces

While CO_2_ promotes H_2_O_2_ formation on both Au and Pd, the markedly different kinetic responses in H_2_O formation rates and H_2_O_2_ selectivity upon CO_2_ introduction suggest that CO_2_-derived species alter the kinetic relevance of elementary steps and metal–adsorbate interactions differently across both metals. On Pd, isotopic measurements confirm that CO_2_-derived intermediates mediate proton transfer to O_2_-derived species to increase H_2_O_2_ rates and selectivity. Interpretation of *operando* attenuated total reflection-infrared (ATR-IR) spectroscopy, temperature programmed oxidation (TPO), and X-ray absorption spectroscopy (XAS) expose the extent of mediation (*i.e.*, surface-bound *versus* liquid phase mediation) and concomitant modifications to Pd nanoparticles induced by CO_2_.

*Operando* ATR-IR spectra collected under H_2_O_2_ reaction conditions (Fig. 6; 200 kPa H_2_, 60 kPa O_2_) reveal the formation of organic surface species on Pd over time in CO_2_-rich conditions (1100 kPa CO_2_). The spectra show features corresponding to adsorbed methyl groups (*ν*_as_(CH_3_) at 2920 cm^−1^ and *ν*_s_(CH_3_) at 2850 cm^−1^), aqueous carbon dioxide (*ν*(CO_2_)_aq._ at 2343 cm^−1^), hydroxyl groups (*δ*(HOH) at 1640 cm^−1^), bicarbonate/carbonates and adsorbed formates (*ν*(OCO) or *ν*_3_(CO_3_^2−^) at 1390 cm^−1^, *ν*_3_(HCO_3_^−^) at 1354 cm^−1^).^50–55^ These feature gradually emerge with increased exposure time to O_2_ reduction conditions in the presence of CO_2_. H_2_O_2_ rates measured in the presence of CO_2_ from the outlet of the infrared cell increase two-fold, similar to results observed from trickle-bed reactor kinetic measurements (Fig. 1). The presence of these features indicates the formation of CO_2_-derived formate as well as methyl species, and carbonate/bicarbonate intermediates adsorbed on Pd that mediate proton electron pathways and increase H_2_O_2_ rates and selectivities on Pd, consistent with prior observations in methanol-containing systems.^15^ These features persist for hours after removal of CO_2_ from the gas-saturated liquid stream (Fig. S19), while H_2_O_2_ rates return to values observed before CO_2_ exposure, which demonstrates that the CO_2_-derived intermediates remain strongly bound to Pd. Additionally, the *operando* spectra with CO_2_ do not show any features corresponding to CO formation that would appear at ∼2100 cm^−1^ on Pd surfaces (Fig. 6 and S19–21). In contrast to Pd, the infrared spectra collected on Au under identical conditions (Fig. S22) show no discernible features associated with organic intermediates or CO under CO_2_, consistent with the absence of surface redox mediation on Au. The CO_2_-derived organic species formed on Pd could potentially migrate from Pd onto the SiO_2_ support. However, reactor calibration experiments indicate that such species do not form in appreciable quantities on bare SiO_2_ under oxygen reduction reaction conditions. While these experiments do not exclude the possibility that organics formed on Pd subsequently migrate to adsorb on SiO_2_, the lack of reactivity towards H_2_O_2_ on SiO_2_ and the absence of CO_2_-derived intermediates on Au-SiO_2_, suggest that any organic species adsorbed on SiO_2_ do not measurably contribute to the observed redox mediation on Pd in the presence of CO_2_.

**Fig. 6:**
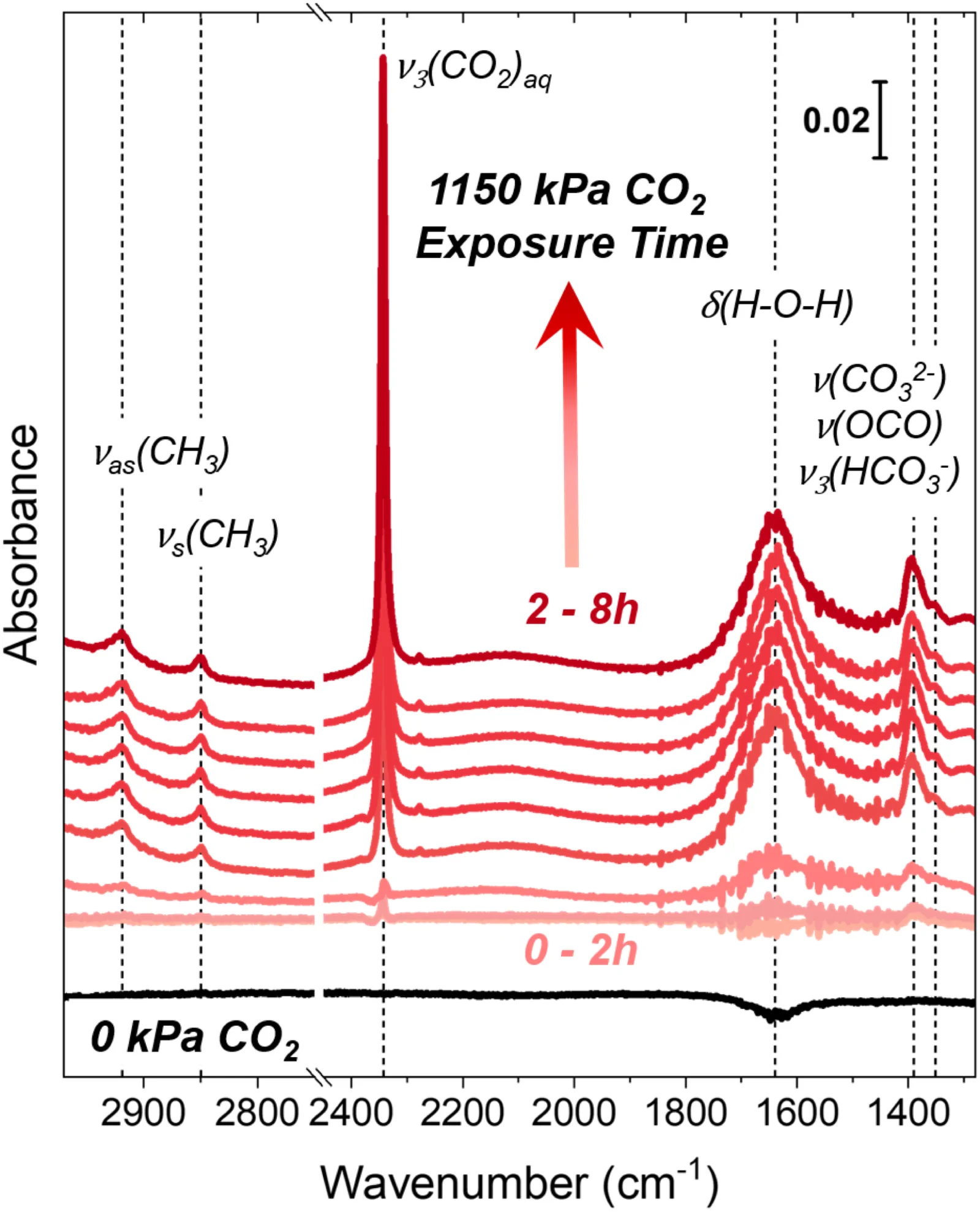
*Operando* infrared spectra of Pd-SiO_2_ accumulating organic intermediates upon CO_2_ exposure over 8 h under thermochemical ORR conditions (200 kPa H_2_, 60 kPa O_2_, 1150 kPa CO_2_, 298 K). The black spectrum (bottom) was obtained after 4 h under CO_2_-free ORR conditions.

The organic surface intermediates generated *in situ* from CO_2_ adsorb on to Pd and occupy a fraction of the available surface sites on Pd. Subsequently, these CO_2_-derived species act as surface redox mediators, facilitating proton–electron transfer to adsorbed oxygen species, thereby promoting H_2_O_2_ formation (Scheme S2). Similar increases in H_2_O_2_ selectivity have been reported upon the addition of methanol,^15^ quinones, and other organic species^16^ that adsorb on catalyst surfaces and participate in proton–electron transfer. The presence of these organic species appears responsible for an increase in the apparent activation enthalpies for H_2_ oxidation and those for H_2_O_2_ and H_2_O formation (Table S7). Together, these observations suggest that CO_2_-derived intermediates alter both the fraction and nature of the available Pd active sites, thereby hindering H_2_ activation and O–O bond rupture, potentially through changes to the kinetic mechanism. TPO profiles of Pd catalysts exposed to CO_2_ under H_2_-rich conditions further confirm the presence of strongly-bound organic species through the appearance of O_2_ consumption and CO_2_ evolution at elevated temperatures (Fig. S25), whereas the TPO profiles of Au show negligible features compared to Pd (Fig. S26). The persistent nature of these CO_2_-derived intermediates on Pd aligns with the irreversible increase in H_2_O_2_ selectivity observed after CO_2_ exposure (Fig. 1). Collectively, these results confirm that formate, methyl, and carbonate species form *in situ* when CO_2_ is added to thermochemical O_2_ reduction reaction conditions, adsorb strongly to Pd and act as surface redox mediators, but do not form in appreciable amounts on Au surfaces to modify reaction pathways.

*Operando* liquid-phase XAS^33^ performed on Pd and Au catalysts reveal structural and electronic changes induced by CO_2_ under thermochemical O_2_ reduction reactions beyond those described by kinetic measurements. Structural parameters including bond distance, coordination number (CN), and Debye–Waller factor were obtained by fitting the extended X-ray absorption fine structure (EXAFS) spectra with models^34,35^ including the first coordination shell only (Table 3) as well as the nearest three shells (Table S8). The Pd–Pd first shell CN of reduced Pd-SiO_2_ under He of 10.7 ± 0.8 appears consistent with an average size of 3.1 nm from CO chemisorption and 3.9 ± 0.8 nm from TEM (Table S1 and Fig. S2).^56^ Analysis of the Fourier transformed (FT) EXAFS spectra of Pd catalysts under H_2_-rich conditions in the absence of CO_2_ (200 kPa H_2_, 60 kPa O_2_) show Pd–Pd scattering shift towards higher *R* values in comparison to spectra obtained at O_2_-rich conditions (40 kPa H_2_, 60 kPa O_2_) and from the *in situ* reduced (metallic) Pd sample under He (Fig. 7a). The Pd–Pd bond distance elongates to 2.823 Å under H_2_ rich conditions, which differs considerably to that of the metallic Pd particle (2.756 Å). This increase in Pd–Pd bond length reflects the formation of a hydride: these values correspond to a H to Pd ratio of ∼0.4 (ref. 15, 57 and 58) and formation of the β-hydride phase (β-PdH_*x*_). As expected, the formation of β-PdH_*x*_ also results in the highest bond disordering and lowest FT magnitude among all the other conditions. Finally, these changes manifest also in a shift of X-ray absorption near edge fine structure (XANES) features to lower energies (peaks at 24 387 and 24 424 eV, Fig. S30 and S31).^59^ Under O_2_-rich reaction conditions in the absence of CO_2_, the XANES features (Fig. S30 and S31), FT-EXAFS spectra, and Pd–Pd bond distance remain similar to the spectra under He, suggesting that the nanoparticles persist as metallic structures under these conditions.

**Table 3:** EXAFS parameters for the Pd–Pd scattering path of Pd-SiO_2_ catalyst under *operando* O_2_ reduction reaction conditions (298 K)

H_2_ partial pressure (kPa)	O_2_ partial pressure (kPa)	CO_2_ partial pressure (kPa)	Radial distance (Å)	First shell coordination number (CN)	*σ* ^2^ × 10^3^ (A^2^)
40	60	0	2.749 ± 0.003	10.2 ± 1.2	6.2 ± 0.8
40	60	1150	2.743 ± 0.002	9.0 ± 0.8	5.9 ± 0.7
200	60	0	2.823 ± 0.004	10.0 ± 1.3	7.9 ± 1.0
200	60	1150	2.754 ± 0.004	8.7 ± 1.3	6.2 ± 1.0

**Fig. 7:**
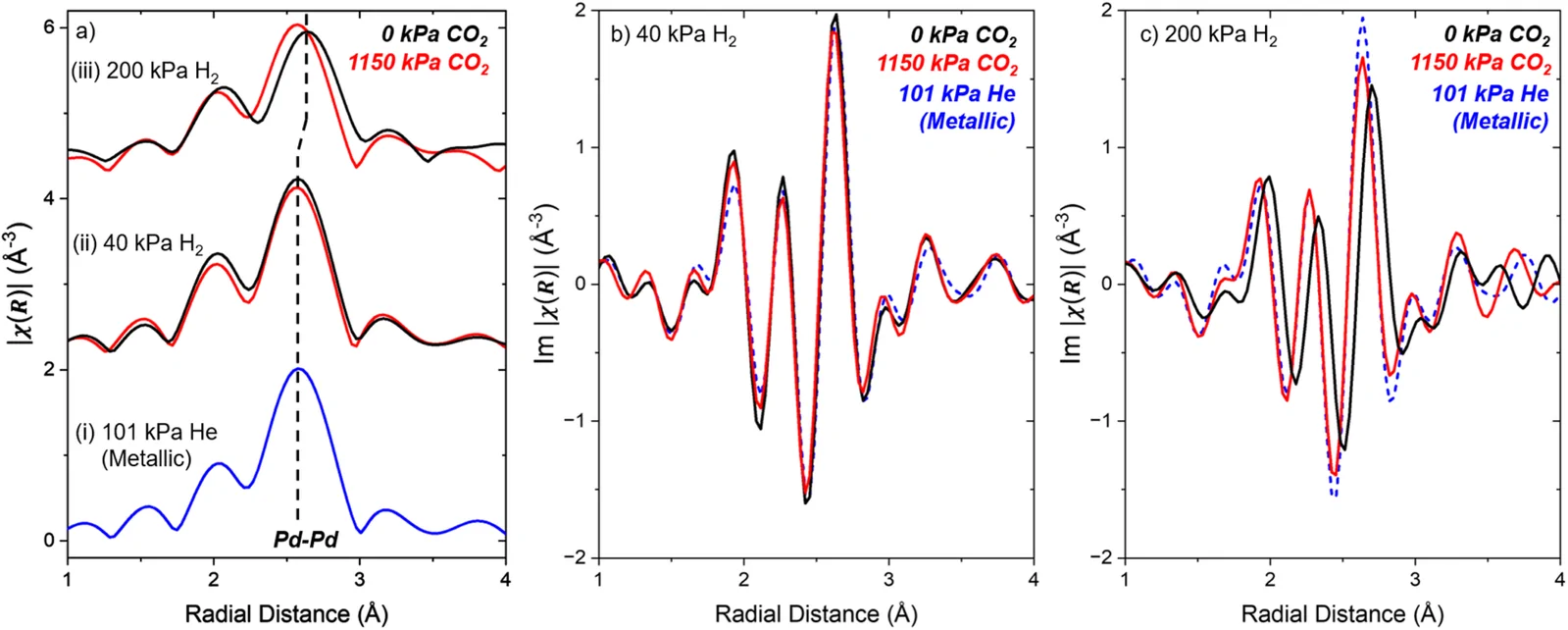
(a) Pd K-edge EXAFS magnitude of the Fourier transformed *k*^2^-weighted *χ*(*k*) data (*k* = 3 to 10.9 Å^−1^) from *in situ* reduced Pd-SiO_2_ under 101 kPa He and *operando* EXAFS measurements at indicated H_2_ pressures with 0 kPa CO_2_ (black) and 1150 kPa CO_2_ (red) (60 kPa O_2_, 298 K). Imaginary *χ*(*R*) data at (b) 40 kPa H_2_ and (c) 200 kPa H_2_ (60 kPa O_2_, 298 K).

Contact of CO_2_ with Pd nanoparticles suppresses the formation of β-PdH_*x*_ phase originally observed under H_2_-rich conditions, as evidenced by the Pd–Pd scattering and bond distance being similar at 2.756 Å under both H_2_-rich and O_2_-rich conditions from the EXAFS spectra and the imaginary part of the spectra (Fig. 7b and c). Furthermore, the Pd–Pd first shell CN decreased under both H_2_- and O_2_-rich conditions in the presence of CO_2_ (Table 3). This decrease in Pd–Pd CN in the presence of CO_2_ may indicate either that the particles evolve from a spherical structure towards a lower symmetry particle shape^56^ or that Pd leaches from the solid due to the lower pH and the mean particle diameter decreases as a consequence. Analysis of sample composition by ICP-OES shows that the Pd content of the as-synthesized catalysts and spent catalysts following exposure to CO_2_ remain similar (Table S1), therefore, the formation of CO_2_-derived residues must modify the structure of the Pd nanoparticles. This interpretation also aligns with a change in the second and third shell CN upon exposure of Pd nanoparticles to CO_2_ (Table S9). Notably, these conclusions do not originate from artifacts in the fitting procedure, because the change in first shell CN appears when either one or three shells are included in the fit. The evident suppression of β-PdH_*x*_ phase and the change in nanoparticle shape induced by contact with mixtures of CO_2_, H_2_, and O_2_ likely reflect the multifold roles of organic species in modifying surface free energies for Pd nanoparticles.^60^ Further evidence for these species emerges in the ΔXANES spectra (taking metallic Pd-SiO_2_ under He as standard, Fig. S30), which contains features between 24 350–24 365 eV that signify the presence of light elements (C-, O-atoms) on the surface. These peaks do not correspond to the signature of adsorbed CO (*e.g.*, peaks between 24 340–24 350 eV),^61^ which agrees with the absence of features indicative of CO* during ATR-IR spectroscopy. The suppression of the β-PdH_*x*_ phase in the presence of carbonaceous species on Pd has been observed during benzaldehyde and furfural hydrogenation.^62,63^ The heat of adsorption for atomic carbon and CH_*x*_ groups on Pd has been theoretically calculated to be more exothermic and favorable than the experimentally measured β-PdH_*x*_ formation enthalpies.^64–68^ Taken together with the diminished kinetic isotope effect for H_2_ activation (Fig. 3a, b and 4a) and the more reducing catalyst potential (Fig. 5a) observed on Pd under high H_2_ and CO_2_ pressures further suggest that CO_2_-derived surface species alter the equilibrium for hydrogen activation such that hydrogen incorporation into the Pd lattice and formation of β-PdH_*x*_ phase become less favorable. In contrast, CO_2_ exposure to Au-SiO_2_ under O_2_ reduction conditions leads to negligible differences in the first shell CN and Au–Au bond distance (Fig. S32 and Table S11), which confirms that CO_2_ neither alters the nanoparticle structure nor forms CO_2_-derived intermediates *via* CO_2_ hydrogenation on Au.

The β-PdH_*x*_ phase formed in purely aqueous systems exhibits greater H_2_O_2_ selectivities (55%) than metallic Pd (22%); however, the suppression of the β-PdH_*x*_ phase in aqueous solutions with CO_2_ leads to even greater selectivities on metallic Pd (90%). Prior research in aqueous methanol solvents showed organics stabilize the β-PdH_*x*_ phase under all reaction conditions, but methanol-derived surface redox mediators increased H_2_O_2_ selectivities only when Pd surfaces were surrounded by large fractions of H_2_O molecules, which helped solvate and stabilize H_2_O_2_ transition states, and co-catalyze reactions involving the mediator.^15^ This work supplements conclusions from prior research^69^ and suggests that H_2_O_2_ selectivities depend more strongly on the nature and identity of organic redox mediators and on the solvation environment of transition states than on the phase of Pd. Taken together, these findings show that CO_2_ induces structural modifications to Pd nanoparticles by forming CO_2_-derived surface intermediates that also mediate proton–electron transfer steps, in addition to CO_2_ increasing proton or hydrogen availability through solvent acidification. The combination of these effects accounts for the two-fold increase in H_2_O_2_ formation rates and ∼90% H_2_O_2_ selectivity observed on Pd catalysts for thermochemical oxygen reduction in the presence of CO_2_.

## 4. Conclusions

The catalytic consequences and molecular origins of the promotional effects of carbon dioxide (CO_2_) in thermochemical O_2_ reduction to hydrogen peroxide (H_2_O_2_) over silica supported Pd and Au nanoparticles were investigated through a combination of kinetic and isotopic measurements, spectroscopy, and open circuit potentiometry measurements. We demonstrate the addition of CO_2_ as a gas diluent in pure aqueous solvents increases H_2_O_2_ rates (two-fold) and selectivities (55% to 90%) on Pd-SiO_2_ catalysts at 278 K without the need for halides, mineral acids, or organic solvents. The addition of CO_2_ brings two significant changes. First, steady-state kinetics indicate that CO_2_ serves as an *in situ* acid promoter by forming carbonic acid, lowering local pH, and increasing rates of proton reduction (Volmer reaction), which increases surface coverages of H-atoms on Pd and Au and doubles H_2_O_2_ formation rates. Second, and specific to Pd alone, isotopic kinetic measurements coupled with *operando* infrared and X-ray absorption spectroscopy reveal that CO_2_ hydrogenates *in situ* and generates organic surface-bound intermediates (formate, methyl-derivatives). These species suppress Pd-hydride formation, inhibit O–O bond scission, and function as surface redox mediators that facilitate proton–electron transfer pathways. Complementary open-circuit potentiometry measurements demonstrate these species permit the catalyst to reside at lower electrical potentials than unmodified Pd or Au nanoparticles, which signifies more rapid and reversible H_2_ activation in the presence of these species.

These findings demonstrate that CO_2_ fundamentally alters catalyst structure, surface coverages, and the local reaction environment to drive significant improvements in both H_2_O_2_ rates and selectivity, which extends beyond the prior expectation that CO_2_ primarily increased reactant solubility in the solvent and reduced secondary pathways for H_2_O_2_ decomposition. More broadly, these findings provide new perspectives on the role of CO_2_ and other purported diluent molecules in catalytic systems: these species may actively participate in the reaction network rather than only modifying bulk properties. This insight emerges from judicious use of kinetic, isotopic, spectroscopic, and electrochemical methods to identify such roles. In particular, CO_2_ may form redox mediators on the surface of metal nanoparticles in the presence of H_2_, and while discussed here for O_2_ reduction reactions, such redox mediators likely facilitate the reduction of other substrates present in aqueous reaction environments and perhaps in the absence of water. These insights open new opportunities to leverage intrinsically safe molecules (*e.g.*, CO_2_) as promoters, mediators, or co-catalysts across a wide range of thermo- and electrochemical transformations without requiring significant changes to catalyst synthesis, composition, or operating conditions.

## Author contributions

S. V. synthesized the nanoparticle catalysts and performed characterization and kinetic measurements. H.-L. Y. performed and analyzed the *operando* X-ray absorption spectroscopy measurements. A. J. S. assisted with spectroscopy measurements. N. R. performed kinetic measurements and assisted with data analysis. S. V. and D. W. F. planned the project and conceptualized the manuscript. The manuscript was drafted by S. V. and H-L. Y. and revised by S. V., H.-L. Y., A. J. S., N. R., A. M. K., and D. W. F. All authors have given approval to the final version of the manuscript.

## Conflicts of interest

There are no conflicts to declare.

## Supplementary Material

SC-OLF-D6SC05682B-s001

## Data Availability

Supplementary information (SI): TEM micrographs, X-ray diffractograms, *ex situ* infrared spectra, supplemental O_2_ reduction kinetics, mass transfer tests, rate expression derivations, isotopic measurements, *operando* attenuated total reflection FT-infrared spectra, *operando* X-ray absorption spectra and fitting parameters, temperature programmed oxidation profiles, and catalyst potential or open circuit potential measurements. See DOI: https://doi.org/10.1039/d6sc05682b.
