# Supplementary material for: In situ reduction of CO2 forms surface redox mediators on nanoparticle catalysts
Source: Chem Sci. 2026 Oct 14. Online ahead of print. doi: 10.1039/d6sc05682b (PMC13663033; doi:10.1039/d6sc05682b)
Supplement: Supplementary file 1 [file SC-OLF-D6SC05682B-s001.pdf]

Supplementary Information (SI) for Chemical Science.
This journal is © The Royal Society of Chemistry

In Situ Reduction of CO_2 Forms Surface Redox Mediators on Nanoparticle Catalysts

Sucharita Vijayaraghavan[1], Hung-Ling Yu[2], Adam J. Senko[1], Nishchay Rathi[1], Ayman M. Karim[2], David W. Flaherty[1]*

[1]School of Chemical and Biomolecular Engineering, Georgia Institute of Technology, GA, 30332, USA
[2]Department of Chemical Engineering, University of Virginia, Charlottesville, VA 22903

*Corresponding Author
Phone: (404)-894-5922
Email: dflaherty3@gatech.edu

S1. Catalyst Characterization

S1.1. Determining Nanoparticle Size, Surface Structure, and Crystal Structure of Catalysts Post CO_2 Exposure

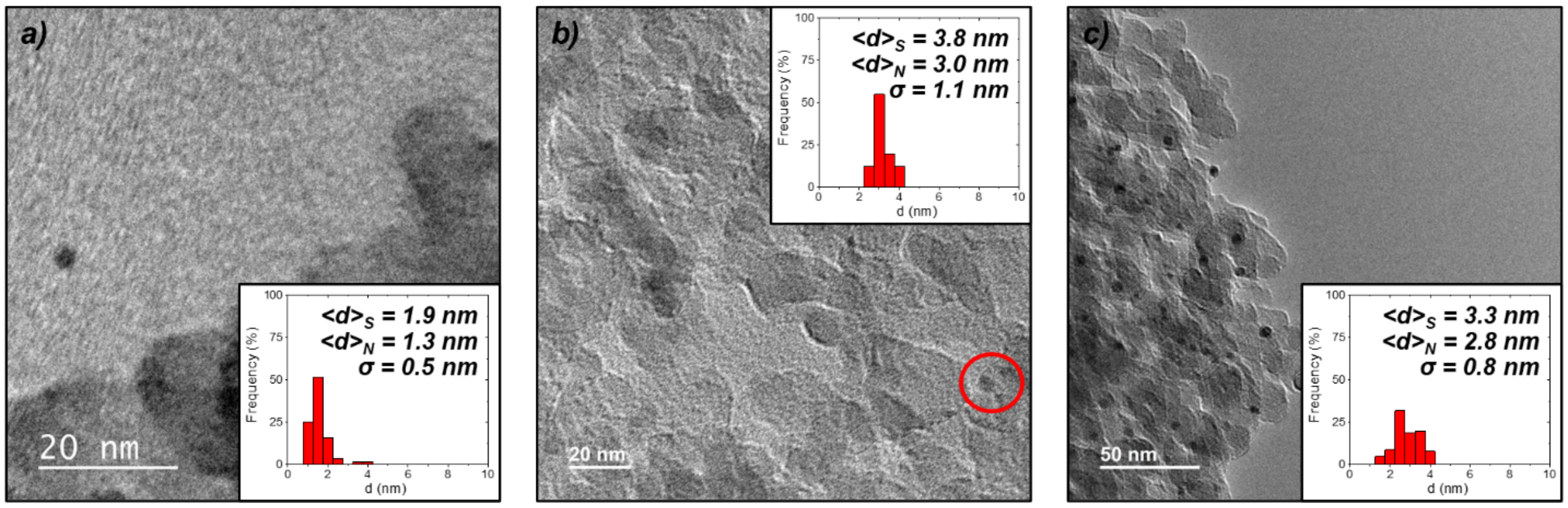

Figure S1. Representative TEM images of as-synthesized (a) low weight loading Pd, (b) high weight loading Pd, and (c) Au catalysts used in the study. Inset shows the particle size distribution, average nanoparticle diameters and standard deviation.

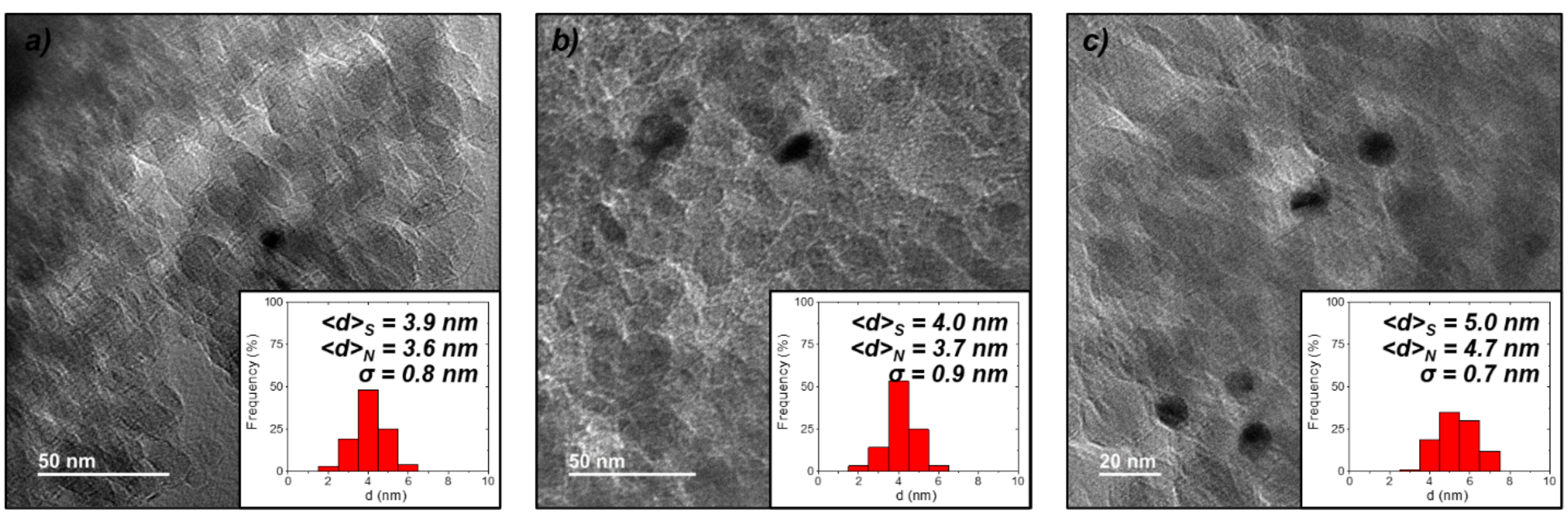

Figure S2. Representative TEM images of spent (a) low weight loading Pd, (b) high weight loading Pd and (c) Au catalysts after exposure to thermochemical oxygen reduction reactions with CO_2 (200 kPa H_2, 60 kPa O_2, 1150 kPa CO_2, 278 K). Inset shows the particle size distribution, average nanoparticle diameters and standard deviation.

Figures S1 and S2 present representative TEM images with particle size distributions as an inset for as-synthesized catalysts and spent catalysts after exposure to steady-state reaction conditions respectively.

The spent catalysts were exposed to reaction conditions for 24 h and vacuum dried at room temperature (298 K) for 24 h prior to characterization.

Table S1. Palladium and gold metal content, and nanoparticle size estimated by TEM and pulse CO chemisorption of catalysts used in the study as-synthesized and after exposure to thermochemical oxygen reduction reactions.

Catalyst	Conditions	Metal Weight Loading (%)	$\langle d \rangle_{s, TEM}$ (nm)	$d_{CO\ Chemi}$ (nm)
$Pd\text{-}SiO_2$[a]	As synthesized	0.02	1.9 ± 0.5	1.6
	24 h post reaction<br>200 kPa H_2-60 kPa O_2-1150 kPa CO_2	N/A	3.9 ± 0.8	3.1
$Pd\text{-}SiO_2$[b]	As synthesized	0.89	3.8 ± 1.1	3.0
	24 h post reaction<br>200 kPa H_2-60 kPa O_2-0 kPa CO_2	0.84	4.1 ± 1.0	3.3
	24 h post reaction<br>200 kPa H_2-60 kPa O_2-1150 kPa CO_2	0.85	4.0 ± 0.9	3.8
$Au\text{-}SiO_2$	As synthesized	1.69	3.3 ± 0.8	N/A
	24 h post reaction<br>200 kPa H_2-60 kPa O_2-0 kPa CO_2	1.61	5.1 ± 1.4	N/A
	24 h post reaction<br>200 kPa H_2-60 kPa O_2-1150 kPa CO_2	1.56	5.0 ± 0.7	N/A

[a]Low weight loading Pd catalyst used for steady-state kinetic measurements and *operando* XAS
[b]High weight loading Pd catalyst used for characterization

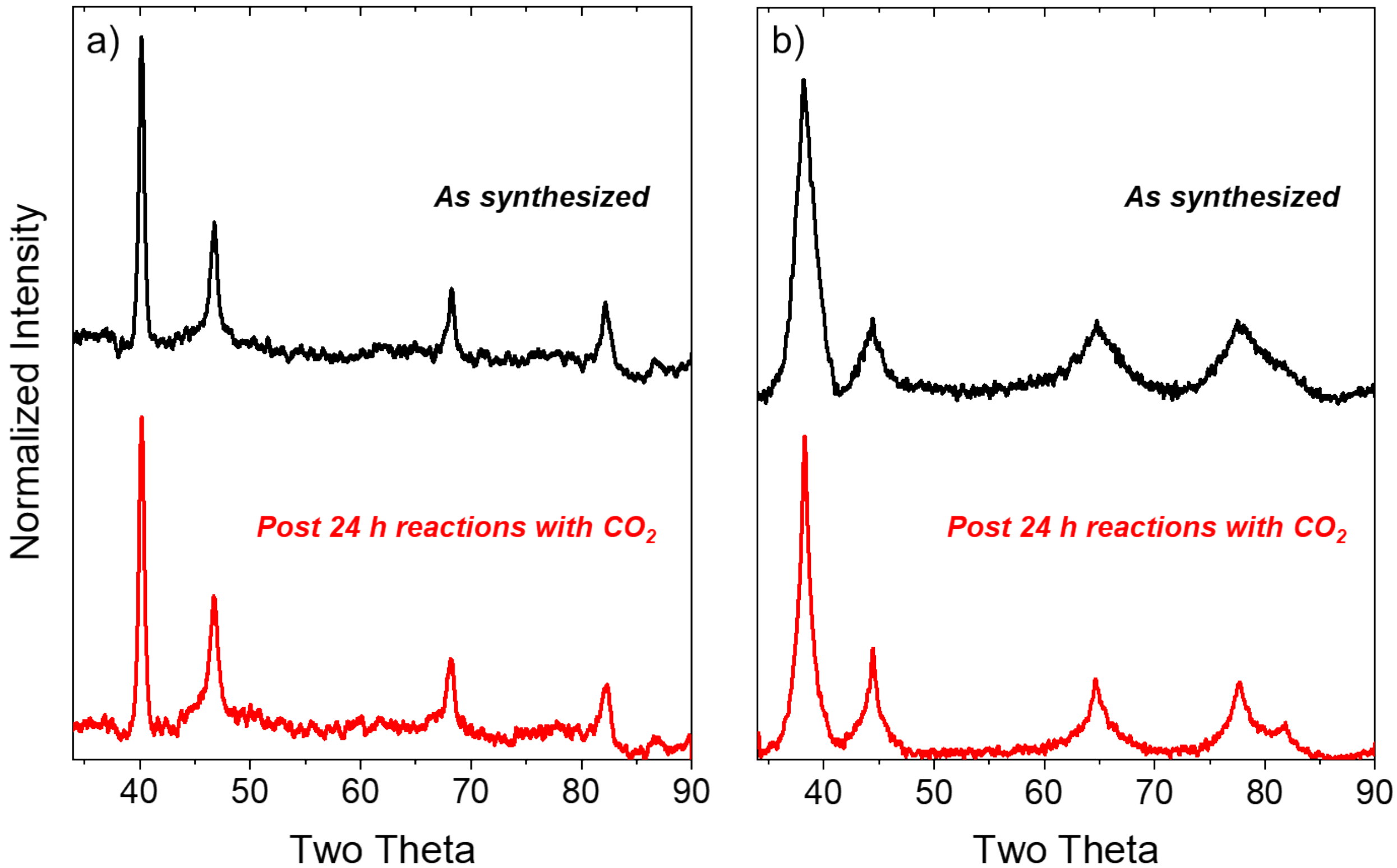

Figure S3. X-ray diffractograms collected *ex situ* in ambient conditions at 298 K over a 2θ range of 10-90° for (a) high weight loading Pd and (b) Au nanoparticles as-synthesized and after exposure to thermochemical oxygen reduction reactions with CO_2 (200 kPa H_2, 60 kPa O_2, 1150 kPa CO_2, 278 K).

Figure S3 presents the XRD patterns for Pd (Fig. S3a) and Au (Fig. S3b) conducted over a 2θ range of 10-90° with a 0.01° step size and a scan rate of 10° min^{-1}. XRD patterns reveal minimal changes to the crystal structure and nanoparticle size before and after CO_2 exposure.

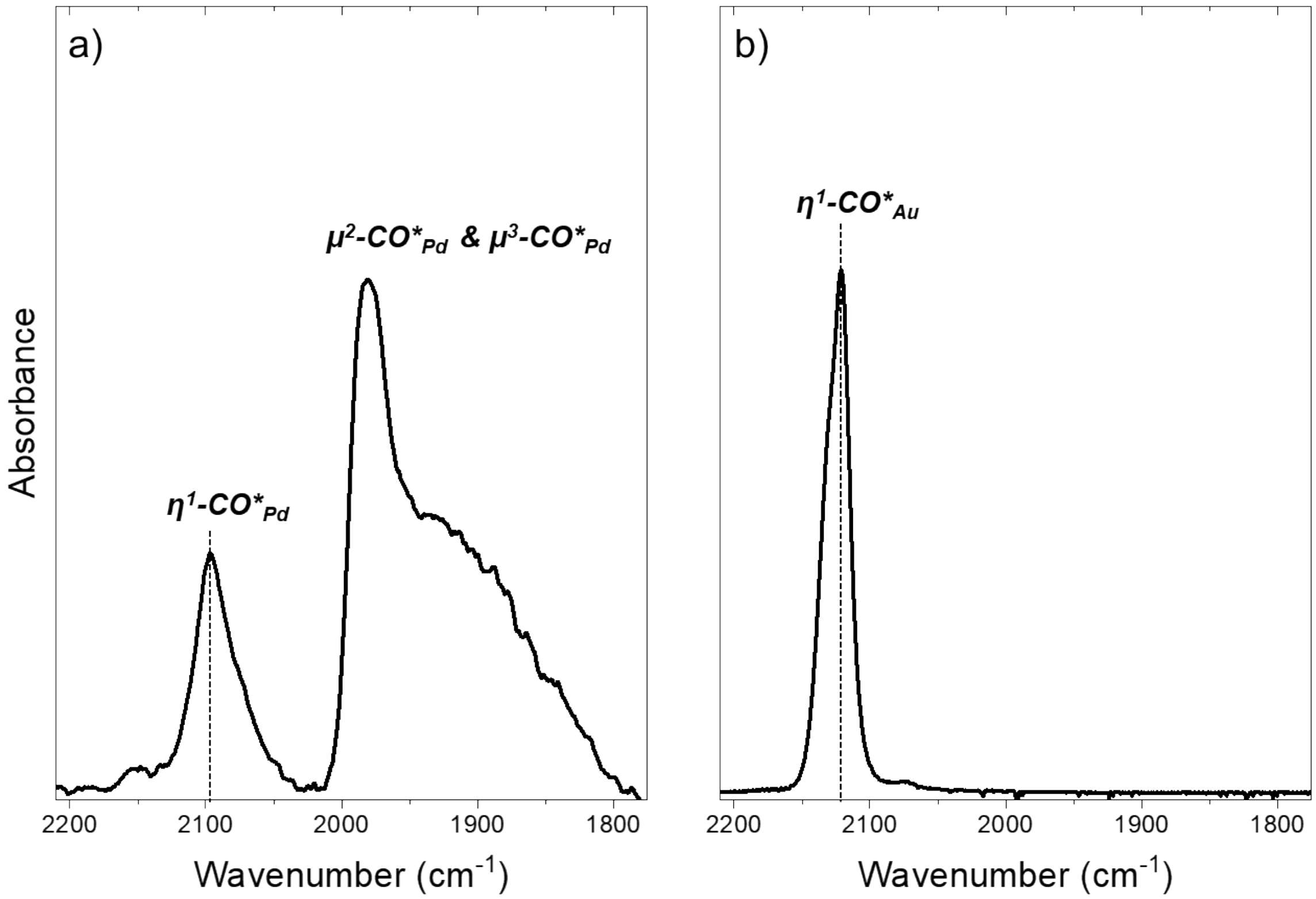

Figure S4. Infrared spectra of adsorbed CO on as-synthesized (a) Pd and (b) Au catalysts at 303 K (0.04 kPa CO) following *in situ* oxidative (20 kPa O_2, 81 kPa Ar, 30 cm^3 min^{-1}, 573 K, 1 h) and reductive pretreatments (20 kPa H_2, 81 kPa Ar, 30 cm^3 min^{-1}, 423 K, 1 h).

Figure S4 shows steady-state spectra of 0.04 kPa CO adsorbed onto as-synthesized Pd (Fig. S4a) and Au (Fig. S4b) at 303 K. The Pd spectra in Fig. S4a shows features corresponding to CO adsorbed linearly atop Pd atoms ($\square^1$-CO^*_{Pd}) at around 2100 cm^{-1}, and multiply bound CO at two- ($\square^2$-CO^*_{Pd}) and three-fold sites ($\square^3$-CO^*_{Pd}) at wavenumbers below 2000 cm^{-1}. Similarly, the spectra for Au shown in Fig. S4b shows only a feature corresponding to CO adsorbed linearly atop Au atoms ($\square^1$-CO^*_{Au}) at around 2120 cm^{-1}.

S1.2. Catalyst Break-In and Mass Transfer Tests in Trickle Bed Reactor

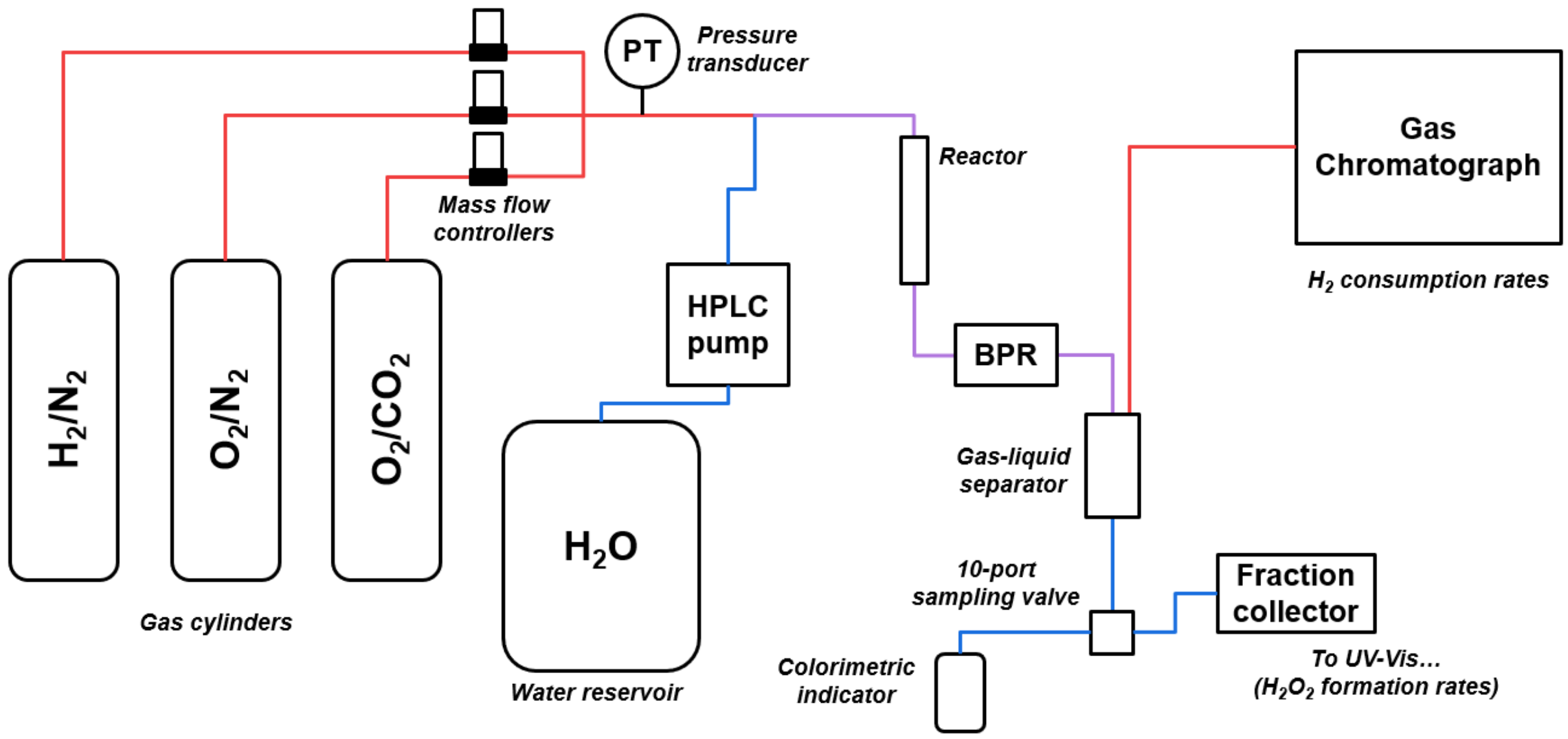

Figure S5. Scheme that depicts the process flow diagram of the trickle bed reactor system used for continuous rate measurements.

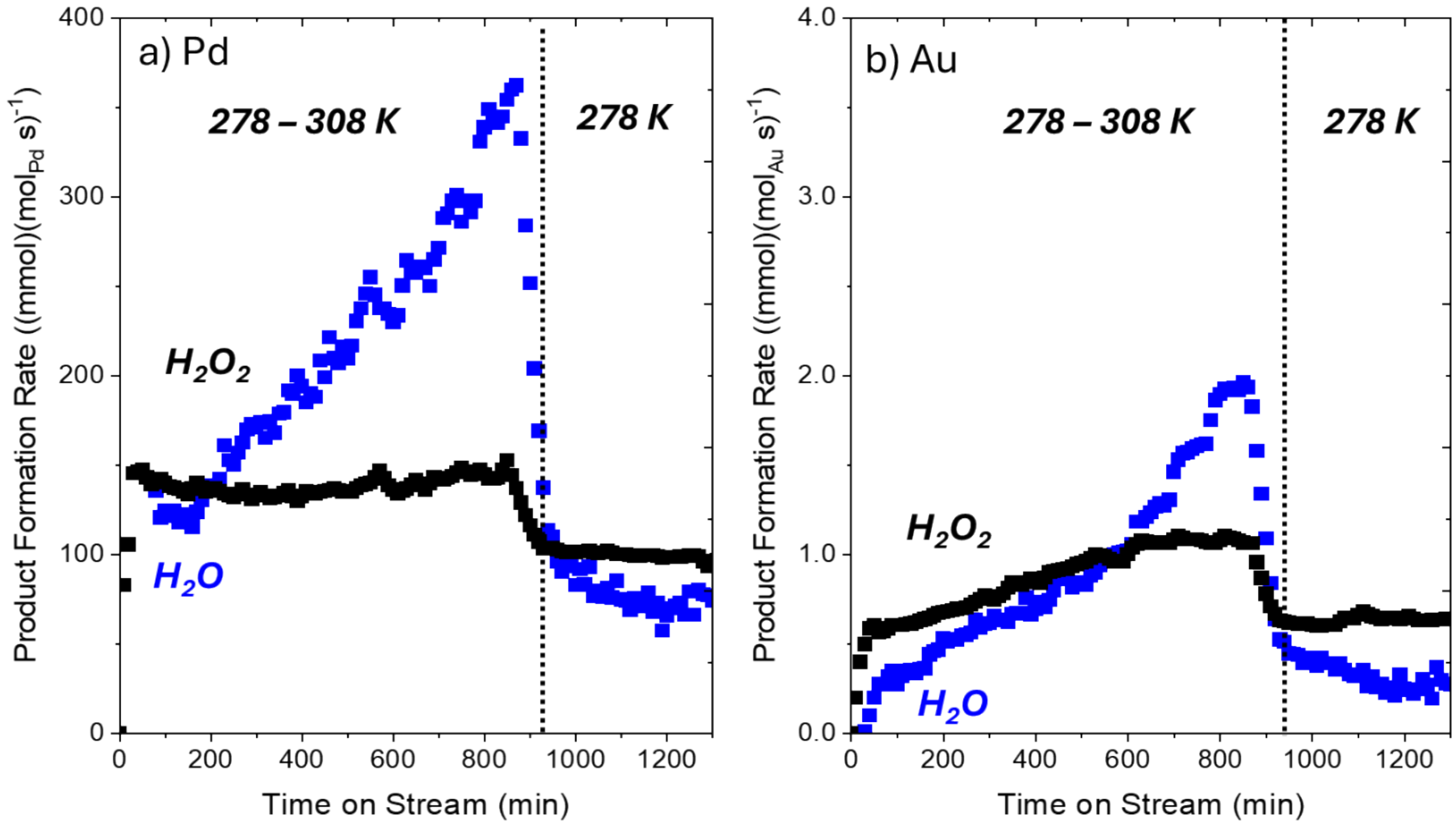

Figure S6. Initial H_2O_2 (black) and H_2O (blue) formation rates as a function of time on stream representing steady-state rates after catalyst break-in for (a) Pd and (b) Au catalysts supported on SiO_2 (200 kPa H_2, 60 kPa O_2, 278 – 308 K break-in, 278 K steady-state).

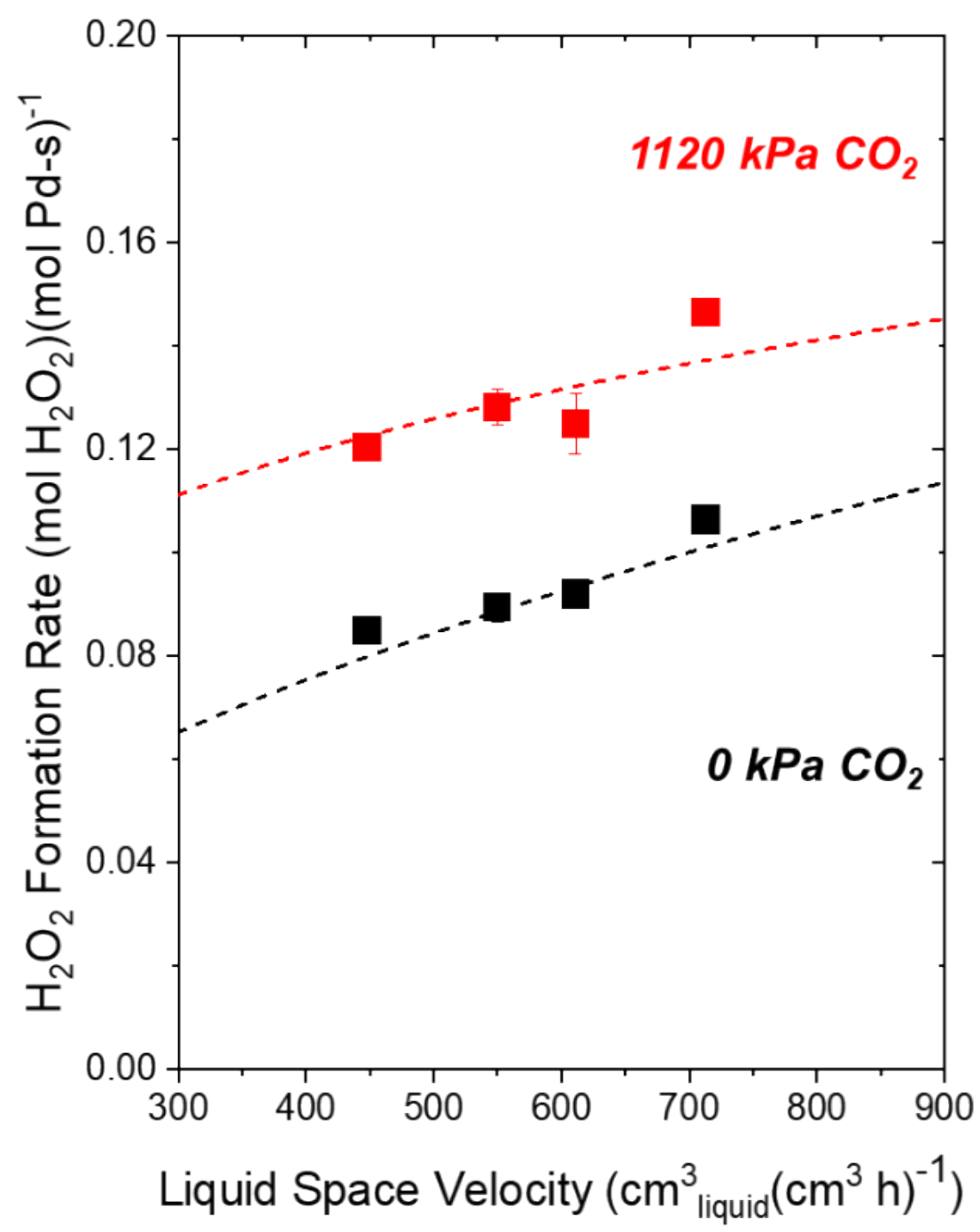

Figure S7. H_2O_2 formation rates as a function of liquid space velocity calculated from the liquid volumetric flow rate on 0.02 wt% Pd-SiO_2 catalysts at 0 kPa CO_2 (black) and 1120 kPa CO_2 (red) in water (200 kPa H_2, 60 kPa O_2, 278 K). Lines are intended to guide the eye.

S1.3. Fitting Parameters for *Operando* X-ray Absorption Spectroscopy

Table S2. Fitting constraints of different shells for *operando* Pd K edge measurements.

Condition	Pd-Pd Coordination shell	Coordination number (-)	Bond disorder σ^2 × 10^3 (Å^2)	∆R(Å)[a]
In- situ reduced, under He	1NN	No constraint	No constraint	No constraint
40 kPa H_2, 60 kPa O_2 Pd/SiO_2	1NN[b]	No constraint	No constraint	No constraint
	2NN	No constraint	No constraint	No constraint
	3NN	No constraint	Same as 2NN	Same as 2NN
	Multiple scattering	15 (set)[c]	2 × 10^{-4} (set)[c]	No constraint
40 kPa H_2, 60 kPa O_2, 1150 kPa CO_2 Pd/SiO_2	1NN	No constraint	No constraint	No constraint
	2NN	No constraint	No constraint	No constraint
	3NN	No constraint	Same as 2NN	Same as 2NN
	Multiple scattering	10 (set)[c]	1 × 10^{-4} (set)[c]	No constraint
200 kPa H_2, 60 kPa O_2 Pd/SiO_2	1NN	No constraint	No constraint	No constraint
	2NN	No constraint	9.0 (set)[c]	No constraint
	3NN	No constraint	7.5 (set)[c]	No constraint
	Multiple scattering	No constraint	2 × 10^{-4} (set)[c]	No constraint
200 kPa H_2, 60 kPa O_2, 1150 kPa CO_2 Pd/SiO_2	1NN	No constraint	No constraint	No constraint
	2NN	No constraint	11 (set)[c]	No constraint
	3NN	No constraint	10 (set)[c]	Same as 2NN
	Multiple scattering	10 (set)[c]	1 × 10^{-4} (set)[c]	No constraint

[a]The bond length reported = 2.751 (the interatomic distance from bulk Pd FCC metal) + ∆R (Å).
[b]The constraints for including only 1NN and multiple shells are the same.
[c]Set refers to the values being set to the constant indicated in the table, due to the limited amount of k space data available.

Due to the limited k space available, the constraints listed in Table S2 is imposed while fitting the Pd K edge EXAFS with paths of the first three shell.

Table S3. Fitting constraints of different shells for *operando* Au L edge measurements.

Condition	Coordination shell Au-Au	Coordination number (-)	Bond disorder σ^2 × 10^3 (Å^2)	∆R(Å)[a]
40 kPa H_2, 60 kPa O_2	1NN	No constraint	No constraint	No constraint
	2NN	No constraint	No constraint	No constraint
	3NN	No constraint	6.5 (set)[b]	No constraint
	Multiple scattering	No constraint	1.3 (set)[b]	No constraint
40 kPa H_2, 60 kPa O_2, 1150 kPa CO_2	1NN	No constraint	No constraint	No constraint
	2NN	No constraint	No constraint	No constraint
	3NN	No constraint	9.0 (set)[b]	No constraint
	Multiple scattering	No constraint	1.3 (set)[b]	No constraint

[a]The bond length reported = 2.885 (the interatomic distance from bulk Au FCC metal) + ∆R (Å).
[b]Set refers to the values being set to the constant indicated in the table, due to the limited amount of k space data available.

The L_3 and L_2 Au-SiO_2 spectra are fitted simultaneously to decrease the fitting uncertainty. The L_3 FT k range and fitting R space is 2.4~11.6 Å^{-1} and 1.7~5.3 Å, respectively. The L_2 FT k range and fitting R space is 3.0~10.4 Å^{-1} and 1.7~3.8 Å, respectively. The available k range for L_2 is limited due to the overlap of L_1 edge, hence the shorter R range. The ∆E_0 and S_0^2 are 5.4 eV and 0.85 for L_3 edge spectra, while for the L_2 edge spectra the ∆E_0 and S_0^2 are 3.3 eV and 1.0, respectively. The fitting constraints for the SiO_2 supported Au catalysts are shown in Table S3.

S1.4. Reactor Setup and Catalyst Potential Profiles for Open Circuit Potential Measurements

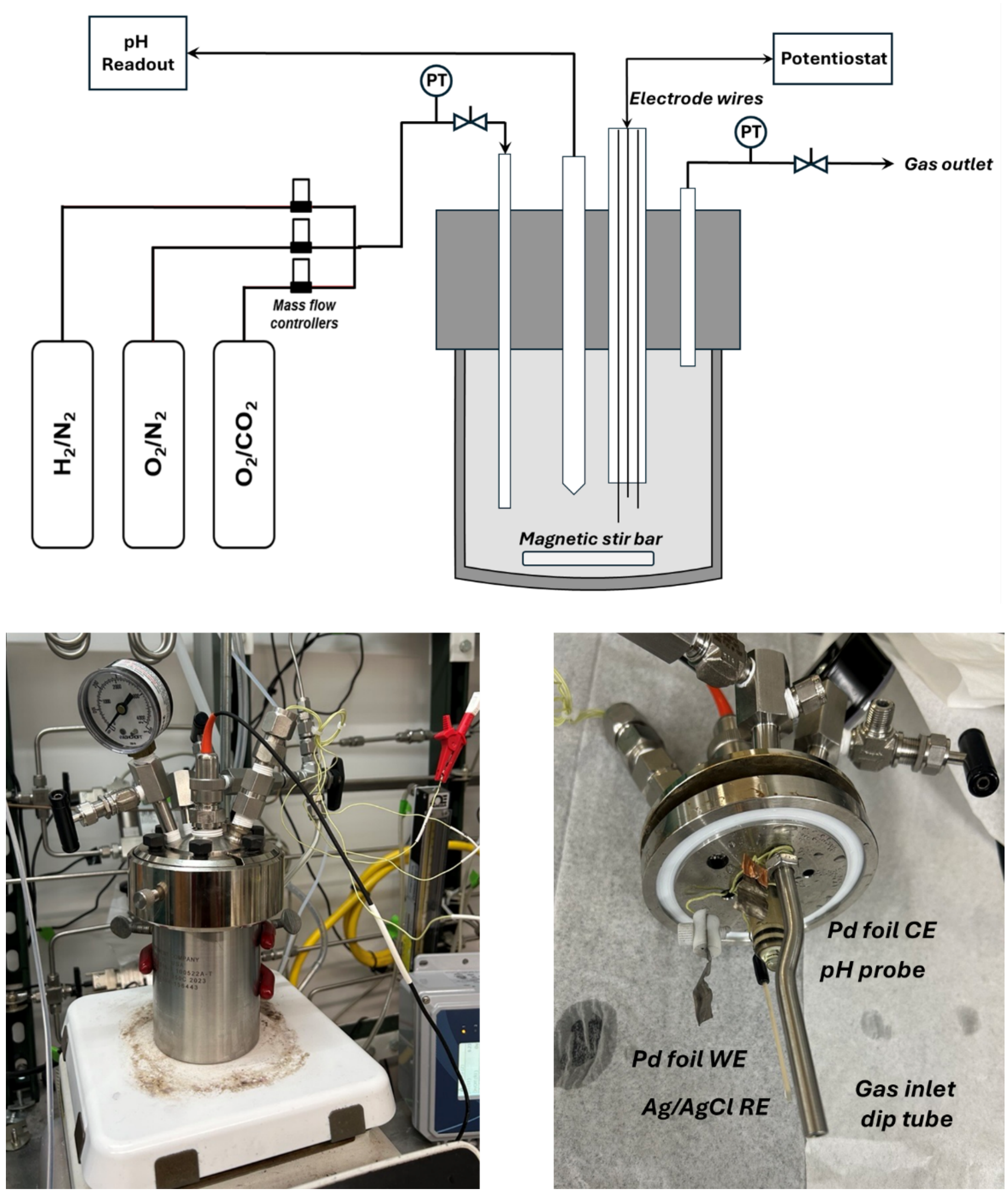

Figure S8. Schematic and images of the pressurized *in situ* open circuit potentiometry Parr reactor setup. This design comprises of a three-electrode setup with a working electrode (WE), a counter electrode (CE), and a leakless Ag/AgCl reference electrode (RE) sealed by compression fittings and connected to a Potentiostat. Additionally, the reactor houses a pH probe, gas inlet and outlet, with the gas inlet dip tube submerged in a 0.1 M sodium perchlorate solution.

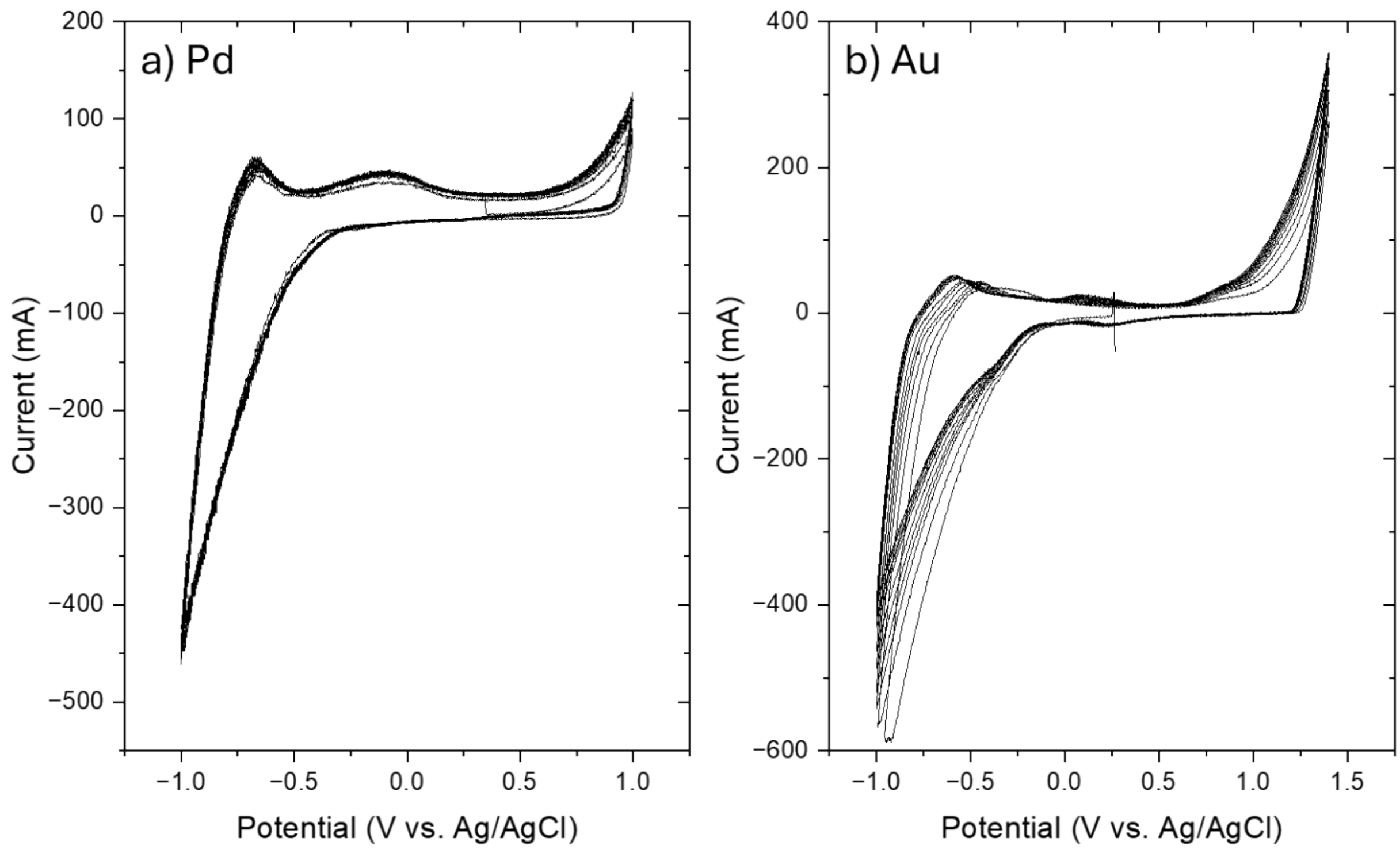

Figure S9. Representative *in situ* cleaning cycling voltammograms of (a) Pd and (b) Au foils (20 cycles, 100 mV s^{-1}) in a cleaning electrolyte solution (0.1 M $NaClO_4$ aqueous solution, 100 cm^3 min^{-1} Ar).

Cyclic voltammetry (20 cycles, -1 to 1 V for Pd and -1 to 1.5 V vs. Ag/AgCl for Au, 100 mV s^{-1}) with a cleaning electrolyte solution (0.1 M aqueous $NaClO_4$) under Ar flow (100 cm^3 min^{-1}) was performed prior to all catalyst potential or open circuit potentiometry measurements. Cleaning was done to ensure any organic surface contaminants were removed from the foils through desorption or reaction. Figure S9 shows 20 cyclic voltammograms overlaid for Pd (S10a) and Au (S10b) foils. Both figures show an oxidation feature between -0.5 to 0.5 V vs. Ag/AgCl corresponding to Pd and Au surface oxidation. The Au cyclic voltammogram also shows two reduction features below 0.2 V vs. Ag/AgCl corresponding to surface gold oxide and hydroxide reduction. Additionally, the oxygen evolution reaction (OER) and hydrogen evolution reaction (HER) features are also prominent at potentials above 0.9 V and below -0.5 V vs. Ag/AgCl respectively. The cyclic voltammograms converged after the tenth or fifteenth cycles as the oxidation and reduction features had minimal changes, indicating that the surfaces no longer contained any adsorbed contaminants.

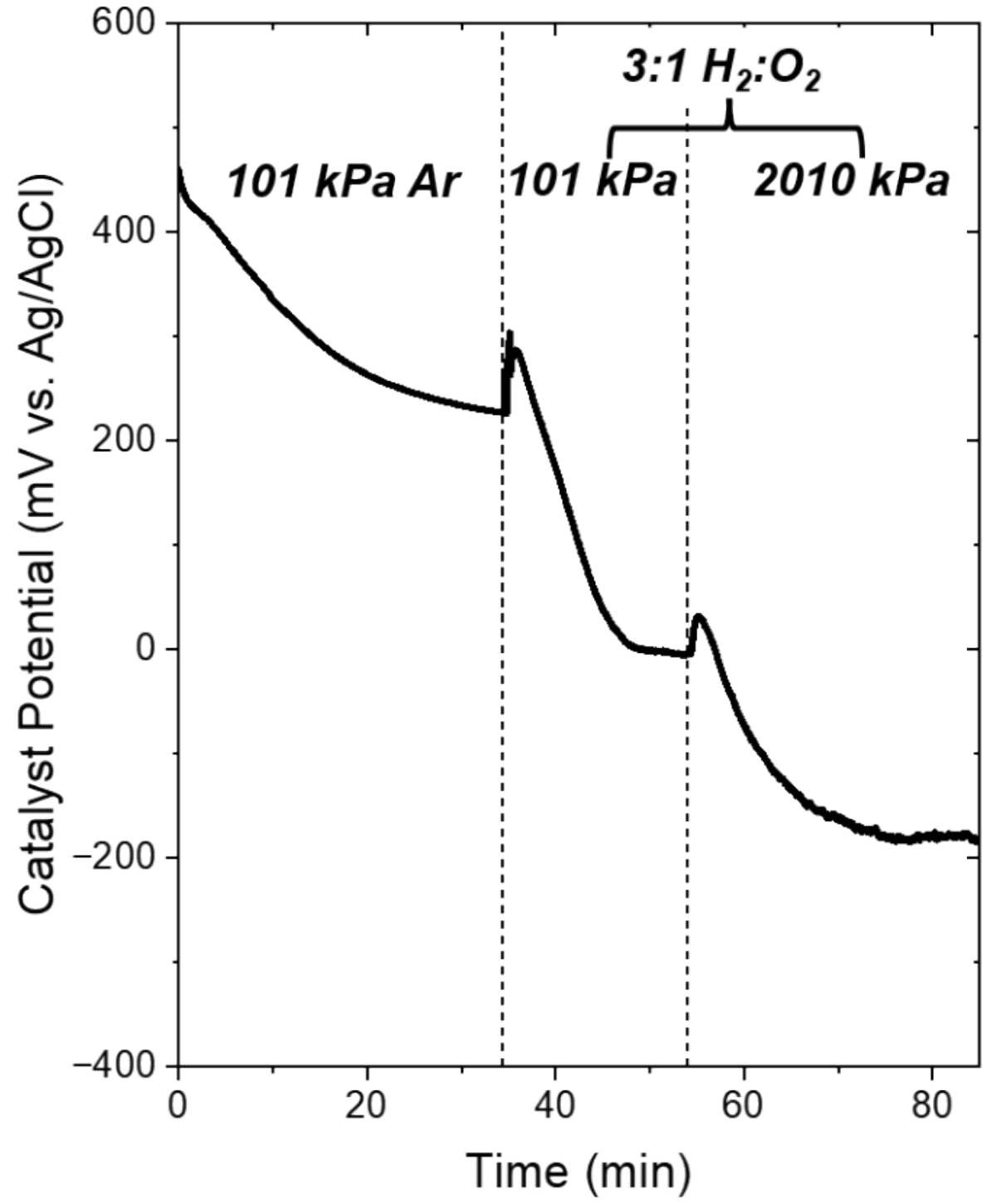

Figure S10. Representative catalyst potential profile of Pd foil as a function of time with changing conditions from Ar at 101 kPa to H_2 and O_2 at a molar ratio of 3:1 at 101 kPa and the reactor pressurized to 2010 kPa with 3:1 molar ratio of H_2: O_2 (200 kPa H_2, 60 kPa O_2, balance N_2, 298 K).

Figure S10 shows a representative transient catalyst potential profile of Pd foil with time approaching steady-state at different conditions. The catalyst potential decreases steadily after continuous Ar exposure post cleaning cyclic voltammograms. A sharp change and a decrease are observed when the gas is switched from Ar to a H_2-rich stream (reducing environment) containing H_2 and O_2 in a molar ratio of 3:1 at ambient pressure. Once the electrode potential reaches steady-state values around 55 min, the Parr reactor is pressurized to 2010 kPa to match thermochemical oxygen reduction reaction conditions in the trickle-bed reactor (200 kPa H_2, 60 kPa O_2, balance N_2). The gases are turned off once the Parr reactor reaches the desired pressure and the final, steady-state value of catalyst potential (~75 – 85 min) is reported in Figures 5, S33-S35 after calculations outlined earlier in this section.

S2. H_2O_2 Rates and Concentrations as a function of Proton Concentration or pH

The effect of pH and proton concentration ($[H^+]$) on Pd and Au catalysts and how CO_2 addition affects $[H^+]$ was tested in a semi batch reactor setup outlined in Section S1.4 using CO_2 and different concentrations of sulfuric acid (H_2SO_4) to obtain the same $[H^+]$ as with CO_2 addition. Figures S12 and S13 show transient H_2O_2 concentration ($[H_2O_2]$) profiles in water and aqueous mixtures of H_2SO_4 as solvents respectively for both Pd and Au catalysts. The concentration profiles for H_2O_2 formation were fitted to equation S2.1. derived previously,[1]

$$[H_2O_2] = \frac{k_1}{k_2}(1 - e^{-k_2 t}) \tag{S2.1}$$

where $[H_2O_2]$ depends on the primary rate constant for H_2O_2 formation (k_1) and secondary H_2O_2 decomposition (k_2) as a function of time (t).

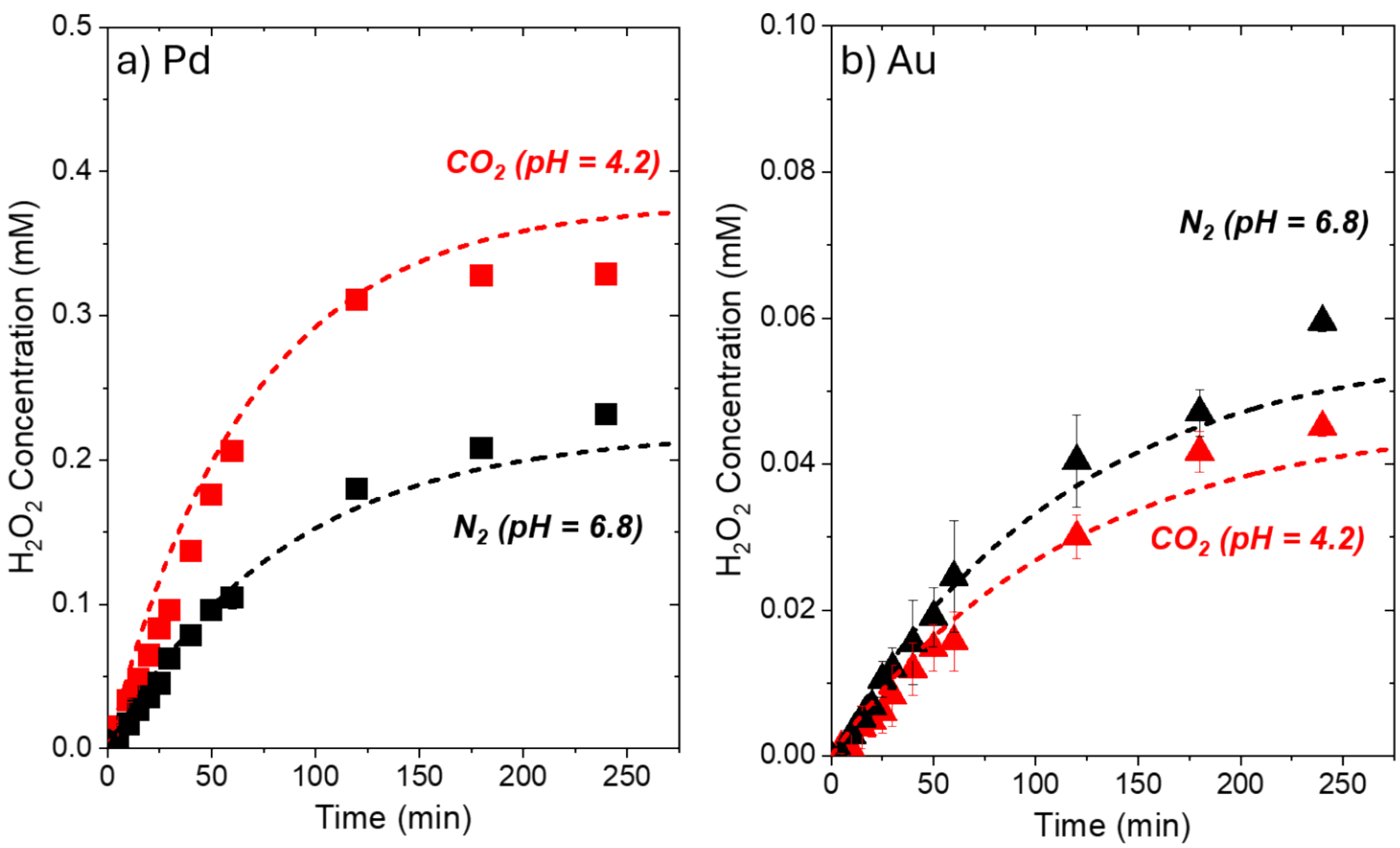

Figure S11. Cumulative H_2O_2 concentration over 4 hours when reacting H_2 with O_2 in water and N_2 (black) or CO_2 (red) over (a) 50 mg of 2 nm Pd-SiO_2, (b) 200 mg of 2 nm Au-SiO_2 (4.8 kPa H_2, 4.8 kPa O_2, 80 cm^3 solvent, 298 K). Dashed lines fitted to equations and analyzed using methods reported previously.[1, 2]

Figure S11 shows H_2O_2 concentration profiles in water with both N_2 and CO_2. The addition of CO_2 to the reactant gases lowers the solution pH from 6.8 to 4.2. On Pd, H_2O_2 concentrations are higher in

CO_2 (lower pH) than in N_2 (S11a) while on Au, H_2O_2 concentrations in CO_2 are lower or similar to those observed in N_2 under these conditions.

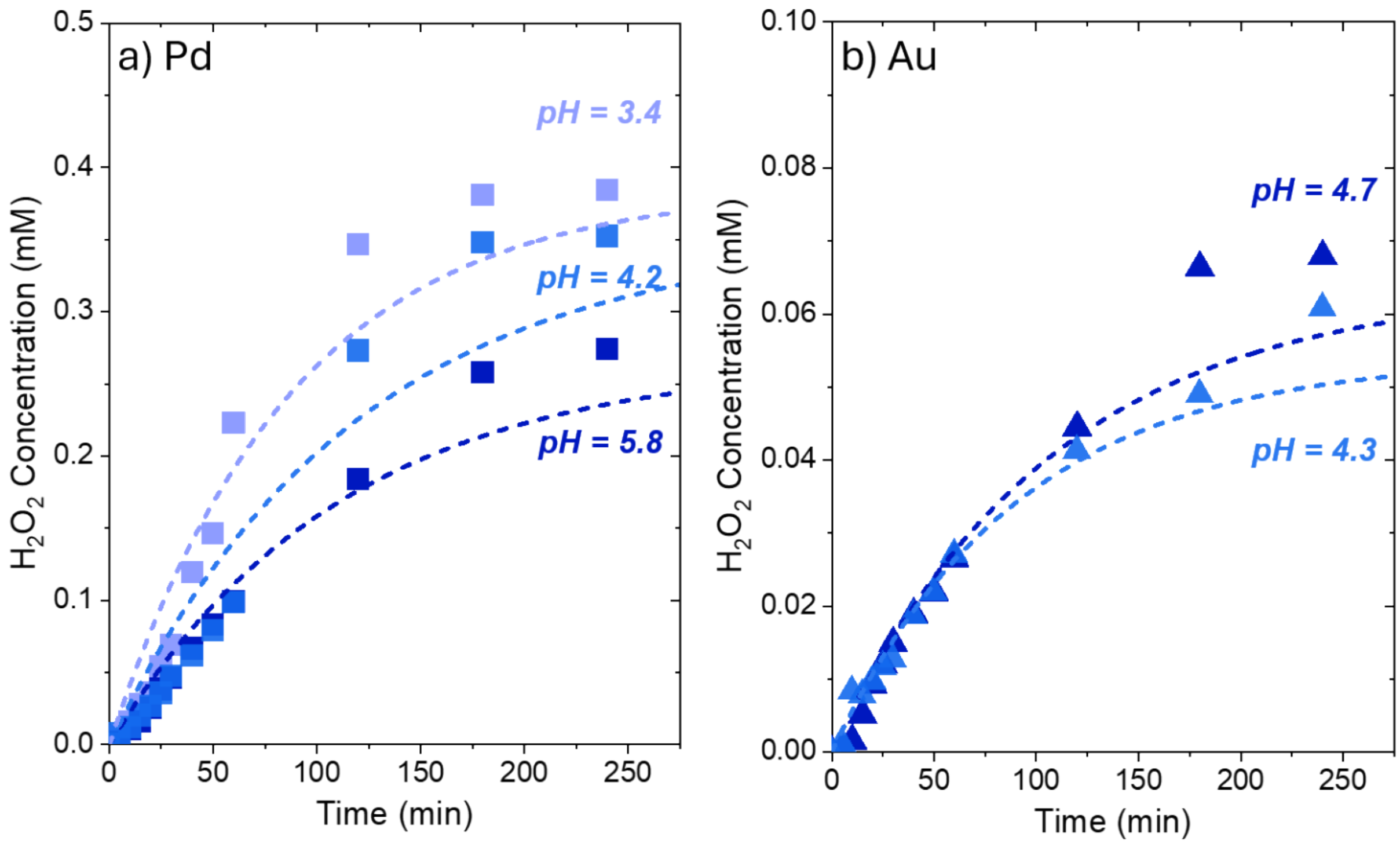

Figure S12. Cumulative H_2O_2 concentration over 4 hours when reacting H_2 with O_2 and N_2 in aqueous sulfuric acid solvent at different pH values over (a) 50 mg of 2 nm Pd-SiO_2, (b) 200 mg of 2 nm Au-SiO_2 (4.8 kPa H_2, 4.8 kPa O_2, 94.4 kPa N_2, 80 cm^3 solvent, 298 K). Dashed lines fitted to equations and analyzed using methods reported previously.[1, 2]

Figure S12 shows concentration profiles of H_2O_2 formation in H_2O solvent containing different sulfuric acid (H_2SO_4) concentrations to change the pH of the reaction solution. Similar to the trends observed with [H_2O_2] as CO_2 lowers the pH in Figure S11, H_2O_2 concentrations increase with decreasing pH with H_2SO_4 in Pd. However, H_2O_2 concentration is not a strong function of pH on Au catalysts. The final H_2O_2 concentrations (at 4 h) observed with CO_2 closely resemble those obtained with H_2SO_4 and N_2 at comparable pH for both Pd and Au catalysts. But values of apparent rate constants for H_2O_2 formation (k_1) and H_2O_2 decomposition (k_2) obtained by fitting the concentration profiles to equation S2.1. (Tables S4 and S5) reveal that reactions with CO_2 show different apparent rate constants that do not follow trends observed with decreasing the pH with H_2SO_4 on both Pd and Au catalysts.

Table S4. Apparent rate constants for H_2O_2 formation (k_1) and H_2O_2 decomposition (k_2) for Pd-SiO_2 catalysts at different pH using different H_2SO_4 concentrations and with CO_2 derived from H_2O_2 concentration profiles from Figures S12 and S13 fitted to equation S2.1.

Conditions / Solvent	pH	Apparent H_2O_2 Formation Rate Constant k_1 ((mmol H_2O_2)(L min)$^{-1}$)	Apparent H_2O_2 Decomposition Rate Constant k_2 (min^{-1})
N_2 / H_2SO_4	3.4	0.0044	0.0114
	4.2	0.0030	0.0085
	5.8	0.0024	0.0090
N_2/ H_2O	6.8	0.0026	0.0118
CO_2 / H_2O	4.2	0.0056	0.0148

Table S5. Apparent rate constants for H_2O_2 formation (k_1) and H_2O_2 decomposition (k_2) for Au-SiO_2 catalysts at different pH using different H_2SO_4 concentrations and with CO_2 derived from H_2O_2 concentration profiles from Figures S12 and S13 fitted to equation S2.1.

Conditions / Solvent	pH	Apparent H_2O_2 Formation Rate Constant k_1 ((mmol H_2O_2)(L min)$^{-1}$)	Apparent H_2O_2 Decomposition Rate Constant k_2 (min^{-1})
N_2 / H_2SO_4	4.3	0.0006	0.0111
	4.7	0.0006	0.0094
N_2/ H_2O	6.8	0.0005	0.0088
CO_2 / H_2O	4.2	0.0004	0.0086

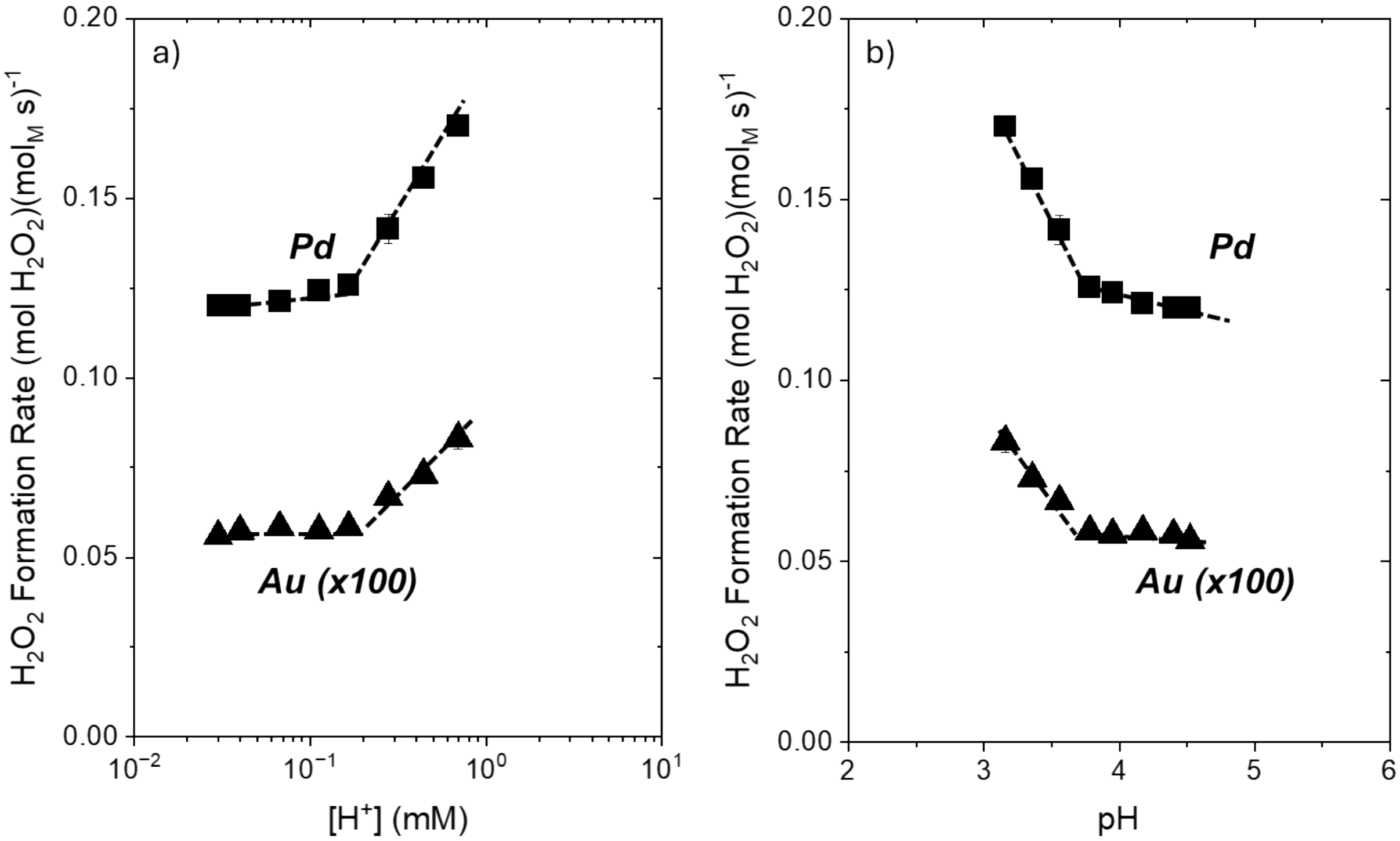

Figure S13. H_2O_2 formation rates as a function of (a) $[H^+]$ and (b) pH from changing CO_2 pressures on Au-SiO_2 (▲; rates multiplied by a factor of 100) and Pd-SiO_2 (□; 200 kPa H_2, 60 kPa O_2, 0-1150 kPa CO_2, 278 K).

Figure S13 shows H_2O_2 formation rates depend on proton concentration ($[H^+]$) and pH for both Pd and Au catalysts. Increasing CO_2 pressure (Figure 2a) increases $[H^+]$ and therefore decreases the pH of the surrounding solvent according to the following equilibrium reactions with CO_2 and H_2O,

$$CO_{2,\,gas} \;\square\; CO_{2,\,dissolved} \qquad \text{(S2.2)}$$

$$CO_2 + H_2O \;\square\; H_2CO_3 \qquad \text{(S2.3)}$$

$$H_2CO_3 \;\square\; HCO_3^- + H^+ \qquad \text{(S2.4)}$$

$$HCO_3^- \;\square\; CO_3^{2-} + H^+ \qquad \text{(S2.5)}$$

CO_2 in equilibrium with H_2O at elevated pressures forms carbonic acid (H_2CO_3, eq. S2.3) which dissociates into bicarbonate (HCO_3^-, eq. S2.4) and carbonate (CO_3^{2-}, eq. S2.5) ions releasing protons in solution. The reactions listed above have associated Henry's law constants (k_h), hydration equilibrium constant (K_h), and acid dissociation constants (K_a) tabulated,[3] given by

$$k_{Henry} = \frac{P_{CO_2}}{[CO_2]} \quad (S2.6)$$

$$K_h = \frac{[H_2CO_3]}{[CO_2][H_2O]} \quad (S2.7)$$

$$K_{a1} = \frac{[HCO_3^-][H^+]}{[H_2CO_3]} \quad (S2.8)$$

$$K_{a2} = \frac{[CO_3^{2-}][H^+]}{[HCO_3^-]} \quad (S2.9)$$

In addition to the reactions associated with CO_2 in equilibrium with water, water equilibrium and ion charge balance equations were also considered,

$$H_2O \square H^+ + OH^- ; K_w = \frac{[H^+][OH^-]}{[H_2O]} \quad (S2.10)$$

$$4[H^+] = [OH^-] + [HCO_3^-] + 2[CO_3^{2-}] \quad (S2.11)$$

Equations S2.6 to S2.11 were solved simultaneously to obtain $[H^+]$ and pH from CO_2 pressures to attain H_2O_2 rate dependencies on $[H^+]$ and pH shown in Figure S13.

S3. Mechanism Derivation for O_2 Reduction Reactions on Au and Pd Surfaces

$$H_2 + \# + H_2O \longrightarrow H^{\#} + e^- + H_3O^+ \quad \text{(1a)}$$

$$H^{\#} + H_2O \longrightarrow \# + e^- + H_3O^+ \quad \text{(1b)}$$

$$O_2 + * \rightleftarrows O_2^* \quad \text{(2)}$$

$$O_2^* + e^- + H^+ \longrightarrow OOH^* \quad \text{(3)}$$

$$OOH^* + e^- + H^+ \longrightarrow H_2O_2^* \quad \text{(4)}$$

$$OOH^* + e^- + H^+ \longrightarrow O^* + H_2O \quad \text{(5)}$$

$$O^* + e^- + H^+ \longrightarrow OH^* \quad \text{(6)}$$

$$OH^* + e^- + H^+ \longrightarrow H_2O^* \quad \text{(7)}$$

$$H_2O_2^* + e^- + H^+ \longrightarrow OH^* + H_2O \quad \text{(8)}$$

$$H_2O_2^* \rightleftarrows H_2O_2 + * \quad \text{(9)}$$

$$H_2O^* \rightleftarrows H_2O + * \quad \text{(10)}$$

Scheme S1. Proposed system of elementary steps for H_2O_2 and H_2O formation via the catalytic reduction of O_2 with H_2 on SiO_2-supported Au and Pd nanoparticles. The symbol → denotes an irreversible reaction, whereas ⇄ signifies a reversible reaction and $\rightleftarrows$ corresponds to a quasi-equilibrated reaction. * and # indicate the independent sites that bind O_2-derived intermediates and H-atoms, respectively.

Rate expressions to describe thermocatalytic O_2 reduction reactions over Au and Pd nanoparticles in the absence of CO_2 are derived following isotopic and kinetic measurements outlined in Section 3.2 of the main text and prior works.[1, 2, 4] Scheme S1 depicts a multi-site model where sites that bind hydrogen are referred to as # and sites that bind oxygen species are * and lists the set of elementary steps involved in thermocatalytic O_2 reduction in water over Au and Pd surfaces. Kinetically relevant H_2 dissociation with the assistance of water (step 1a) generates chemisorbed hydrogen atoms ($H^{\#}$), which oxidize to form hydronium ions and electrons (step 1b). Concurrently, quasi-equilibrated O_2 adsorption yields O_2^* (step 2), which reduces to form OOH* irreversibly through proton electron transfer *via* liquid H_2O (step 3). The OOH* undergoes a second proton electron transfer process to reduce to $H_2O_2^*$ (step 4) or dissociate into H_2O^* and O* (step 5) when O-O bonds in OOH* cleave. The resulting O* or $H_2O_2^*$ can further reduce to form OH* (steps 6 and 8 respectively). Oxygen intermediates OH* ultimately reduce through proton electron transfer steps to form water (H_2O^*; step 7). Both $H_2O_2^*$ and H_2O^* desorb in a quasi-equilibrated manner into solution to yield H_2O_2 and H_2O (steps 9 and 10).

Kinetic isotope effect experiments, and rate dependencies on reactant pressures outlined in Section 3.2. suggests that the kinetically relevant consumption of H_2 ($-r_{H_2}$) limits the total rate of O_2 reduction ($-r_{O_2}$) on both Pd and Au catalysts. The reaction rate for H_2 consumption depends on water-assisted H_2 activation (step 1a) during H_2O_2 and H_2O formation, as given by

$$-r_{H_2} = k_{1a}[H_2][H_2O][\#] = r_{H_2O_2} + r_{H_2O} \quad \text{(S3.1)}$$

where k_i is the rate constant for step *i*, [#] is the number of unoccupied, available sites that bind H_2 and $[H_2]$ and $[H_2O]$ reflect activities of the respective species. Additionally, rate of O_2 reduction can be written as,

$$-r_{O_2} = r_3 = \frac{1}{2}r_{H_2O} + r_{H_2O_2} \quad \text{(S3.2)}$$

Rates of H_2O_2 and H_2O formation can be expanded and written as,

$$r_{H_2O_2} = r_4 = k_4[OOH^*][H^+][e^-] \quad \text{(S3.3)}$$

$$r_{H_2O} = r_5 = 2k_5[OOH^*][H^+][e^-] \quad \text{(S3.4)}$$

with a dependence on the activity of electrons generated from H_2 activation and the coverage of adsorbed peroxy ([OOH*]) intermediate.

The pseudo-steady state hypothesis can be applied on the peroxy species

$$\frac{d[OOH^*]}{dt} = \left(k_3[O_2^*] - (k_4 + k_5)[OOH^*]\right)[H^+][e^-] = 0 \quad \text{(S3.5)}$$

to yield,

$$[OOH^*] = \frac{k_3}{k_4 + k_5}[O_2^*] \quad \text{(S3.6)}$$

where the coverage of adsorbed peroxy species depends on the relative rate that adsorbed oxygen reduces to OOH* by proton-electron transfer (k_3) versus the rate that [OOH*] reduces to form either H_2O_2 (k_4) or H_2O (k_5).

Combining equations S3.3, S3.4, and S3.6 gives

$$r_{H_2O_2} = \left(\frac{k_4 k_3}{k_4 + k_5}\right)[O_2^*][H^+][e^-] = \left(\frac{k_4}{k_4 + k_5}\right) r_3 \quad (S3.7)$$

and

$$r_{H_2O} = \left(\frac{2k_5 k_3}{k_4 + k_5}\right)[O_2^*][H^+][e^-] = \left(\frac{2k_5}{k_4 + k_5}\right) r_3 \quad (S3.8)$$

Equations S3.7 and S3.8 suggest that O_2 reduction rate reflects the ratio of OOH* hydrogenation (k_4) to dissociation (k_5) which governs H_2O_2 selectivity.

Combining equations S3.1, S3.7, and S3.8 yields

$$r_3 = \left(\frac{k_4 + k_5}{k_4 + 2k_5}\right) k_{1a}[H_2][H_2O][\#] \quad (S3.9)$$

Substituting equation S3.9 into S3.7 and S.8 gives

$$r_{H_2O_2} = \left(\frac{k_4}{k_4 + 2k_5}\right) k_{1a}[H_2][H_2O][\#] \quad (S3.10)$$

and

$$r_{H_2O} = \left(\frac{2k_5}{k_4 + 2k_5}\right) k_{1a}[H_2][H_2O][\#] \quad (S3.11)$$

Where H_2O_2 and H_2O formation rates depend directly on H_2 activity.

H_2 activation (steps 1a and 1b) is predominantly irreversible on Au and at low H_2 pressures on Pd as evidenced by HD scrambling experiments in Figure 3 in the main text. Hence, pseudo steady-state hypothesis can be applied to the coverage of sites that bind H_2 ($[H^\#]$).

$$\frac{d[H^\#]}{dt} = k_{1a}[H_2][H_2O][\#] - k_{1b}[H^\#][H_2O] = 0 \quad (S3.12)$$

to obtain,

$$[H^{\#}] = \frac{k_{1a}}{k_{1b}}[H_2][\#] \tag{S3.13}$$

Performing a total site balance on the sites that bind hydrogen ($[L_{\#}]$) shows

$$[L_{\#}] = [\#] + [H^{\#}] \tag{S3.14}$$

and

$$[L_{\#}] = [\#]\left(1 + \frac{k_{1a}}{k_{1b}}[H_2]\right) \tag{S3.15}$$

Complete H_2O_2 and H_2O turnover rate expressions can be obtained by combining equations S3.10 and S3.11 with S3.15.

$$\frac{r_{H_2O_2}}{[L_{\#}]} = \left(\frac{k_4}{k_4 + 2k_5}\right)\left(\frac{k_{1a}[H_2][H_2O]}{1 + \frac{k_{1a}}{k_{1b}}[H_2]}\right) \tag{S3.16}$$

$$\frac{r_{H_2O}}{[L_{\#}]} = \left(\frac{2k_5}{k_4 + 2k_5}\right)\left(\frac{k_{1a}[H_2][H_2O]}{1 + \frac{k_{1a}}{k_{1b}}[H_2]}\right) \tag{S3.17}$$

where H_2O_2 and H_2O formation rates depend on the rate of hydrogen consumption multiplied by the selectivity to either product.

Figures 2b and 2c in the main text shows that rates increase linearly with H_2 pressure on both Pd and Au catalysts at these conditions (10-80 kPa H_2 for Pd, 10-400 kPa H_2 for Au, 60 kPa O_2), such that $H^{\#}$ species exist at low fractional coverages and oxygen-derived species are the most abundant reactive intermediates (MARI). Therefore, equations S3.16 and S3.17 can be simplified to obtain the final rate expressions for H_2O_2 and H_2O formation.

$$\frac{r_{H_2O_2}}{[L_{\#}]} = \left(\frac{k_{1a}k_4}{k_4 + 2k_5}\right)[H_2][H_2O] \tag{S3.18}$$

$$\frac{r_{H_2O}}{[L_{\#}]} = \left(\frac{2k_{1a}k_5}{k_4 + 2k_5}\right)[H_2][H_2O] \tag{S3.19}$$

At high H_2 pressures, rates of H_2O_2 and H_2O formation do not change with increasing H_2 pressure on Pd catalysts (>100 kPa H_2, 60 kPa O_2) and H_2 activation (steps 1a and 1b) are quasi-equilibrated.

Performing a similar derivation, the rate expressions for H_2O_2 and H_2O formation at high H_2 pressures on Pd catalysts are,

$$\frac{r_{H_2O_2}}{[L]} = \frac{\frac{2k_3K_2k_4}{(k_4+k_5)}[H_2O][O_2]}{1+K_2\left(1+\frac{k_3}{k_4+k_5}+\frac{k_5k_3}{k_4+k_5}\left(\frac{1}{k_6}+\frac{1}{k_7}\right)\right)[O_2]} \tag{S3.20}$$

$$\frac{r_{H_2O}}{[L]} = \frac{\frac{4k_3K_2k_5}{(k_4+k_5)}[H_2O][O_2]}{1+K_2\left(1+\frac{k_3}{k_4+k_5}+\frac{k_5k_3}{k_4+k_5}\left(\frac{1}{k_6}+\frac{1}{k_7}\right)\right)[O_2]} \tag{S3.21}$$

These expressions state that H_2O_2 and H_2O rates on Pd will initially increase with O_2 pressures before approaching constant values at high O_2 pressures (>100 kPa O_2).

$$H_2 + \# + H_2O \rightleftarrows H^{\#} + e^- + H_3O^+ \tag{1a}$$

$$H^{\#} + H_2O \rightleftarrows \# + e^- + H_3O^+ \tag{1b}$$

$$H^+ + e^- + \# \rightleftarrows H^{\#} \tag{1'}$$

$$O_2 + * \overset{\bigcirc}{\rightleftarrows} O_2^* \tag{2}$$

$$O_2^* + e^- + H^+ \longrightarrow OOH^* \tag{3}$$

$$O_2^* + 2HCOOH^* \longrightarrow OOH^* + HCOOH^* + HCOO^* \tag{3'}$$

$$OOH^* + e^- + H^+ \longrightarrow H_2O_2^* \tag{4}$$

$$OOH^* + 2HCOOH^* \longrightarrow H_2O_2^* + HCOOH^* + HCOO^* \tag{4'}$$

$$OOH^* + e^- + H^+ \longrightarrow O^* + H_2O \tag{5}$$

$$OOH^* + 2HCOOH^* \longrightarrow O^* + H_2O + HCOOH^* + HCOO^* \tag{5'}$$

$$O^* + e^- + H^+ \longrightarrow OH^* \tag{6}$$

$$OH^* + e^- + H^+ \longrightarrow H_2O^* \tag{7}$$

$$H_2O_2^* + e^- + H^+ \longrightarrow OH^* + H_2O \tag{8}$$

$$H_2O_2^* \overset{\bigcirc}{\rightleftarrows} H_2O_2 + * \tag{9}$$

$$H_2O^* \overset{\bigcirc}{\rightleftarrows} H_2O + * \tag{10}$$

$$CO_2 + H_2O \overset{\bigcirc}{\rightleftarrows} H_2CO_3 \tag{11}$$

$$H_2CO_3 \overset{\bigcirc}{\rightleftarrows} HCO_3^- + H^+ \tag{12}$$

Scheme S2. Proposed system of elementary steps for H_2O_2 and H_2O formation via the catalytic reduction of O_2 with H_2 on SiO_2-supported Au and Pd nanoparticles in the presence of CO_2. The symbol → denotes an irreversible reaction, whereas ⇄ signifies a reversible reaction and $\overset{\bigcirc}{\rightleftarrows}$ corresponds to a quasi-equilibrated reaction. * and # indicate the independent sites that bind O_2-derived intermediates and H-atoms, respectively.

Scheme S2 depicts the proposed list of elementary steps for CO_2 mediated O_2 reduction reactions to form H_2O_2 and H_2O. Steps 11 and 12 represent the formation and dissociation of carbonic acid from equilibrium reactions between CO_2 and H_2O. The additional steps that arise from CO_2 addition are denoted by prime (').Step 1' involves Volmer-type conversion of solution-phase H_3O^+ generated from CO_2 (Steps 11-12), forming additional chemisorbed hydrogen. Steps 3', 4', and 5' represent formate-mediated steps involved in reducing adsorbed oxygen (O_2^*) to OOH^*, $H_2O_2^*$, and H_2O.

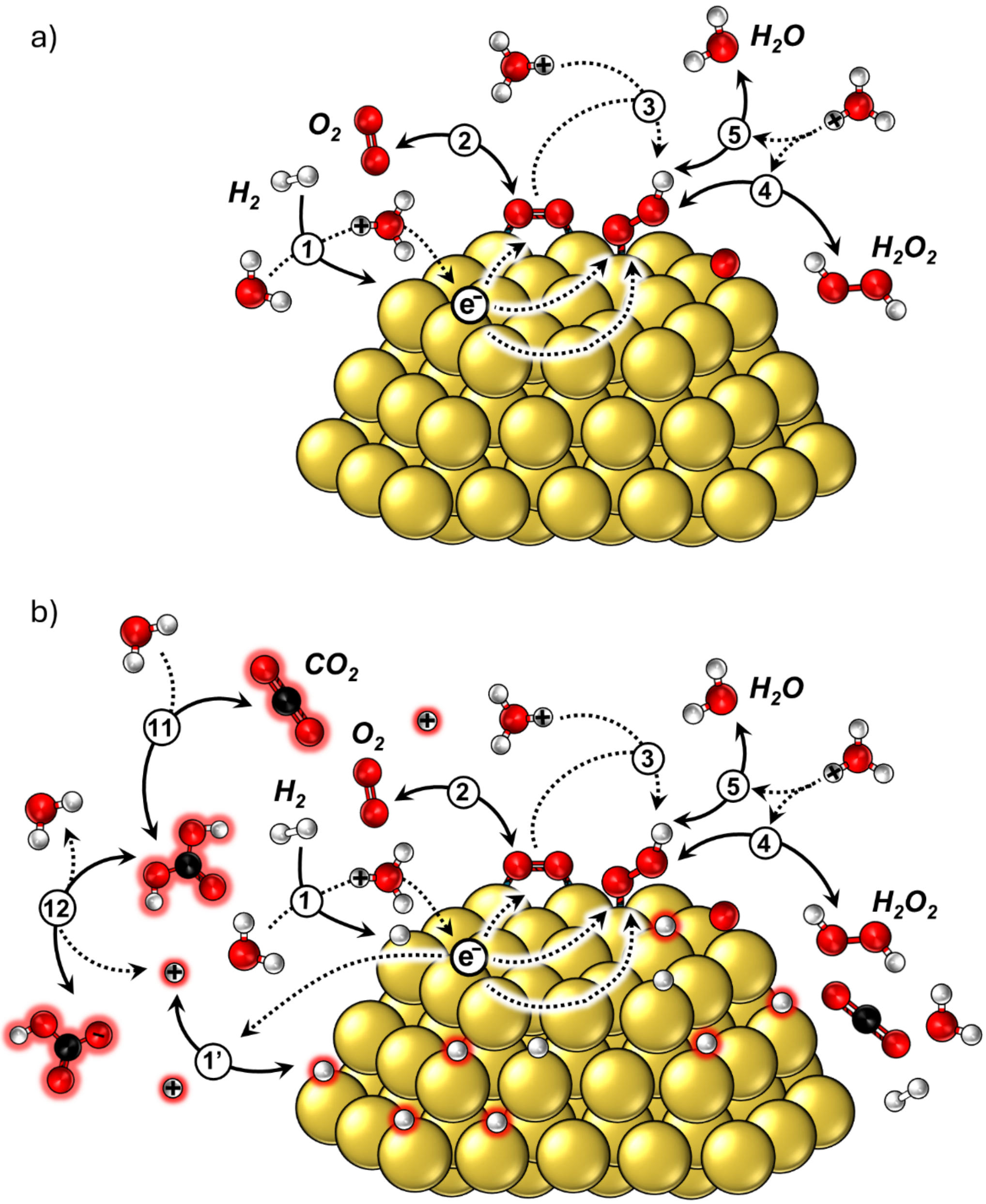

Scheme S3. Simplified elementary steps for primary H_2O_2 and H_2O formation by catalytic O_2 reduction on Au-SiO_2 catalysts in the (a) absence of CO_2, and (b) proposed changes to the mechanism in the presence of CO_2. Dashed lines represent proton-electron transfer steps. The symbol → denotes irreversible reactions and ↔ signifies reversible or quasi-equilibrated reactions.

Scheme S3 illustrates a simplified version of the elementary steps outlined in Scheme S1 for the thermochemical O_2 reduction reaction in the absence of CO_2 (Scheme S3a), and the additional steps involved in the presence of CO_2 in Scheme S3b (listed in Scheme S2). For Au, this only involves the formation and dissociation of carbonic acid from equilibrium reactions between CO_2 and H_2O to

introduce additional H^+, increasing the coverage of chemisorbed hydrogen but no redox mediation effects from CO_2-derived surface intermediates.

S4. Supplementary Kinetics on the Mechanism of O_2 Reduction Reactions on Pd and Au

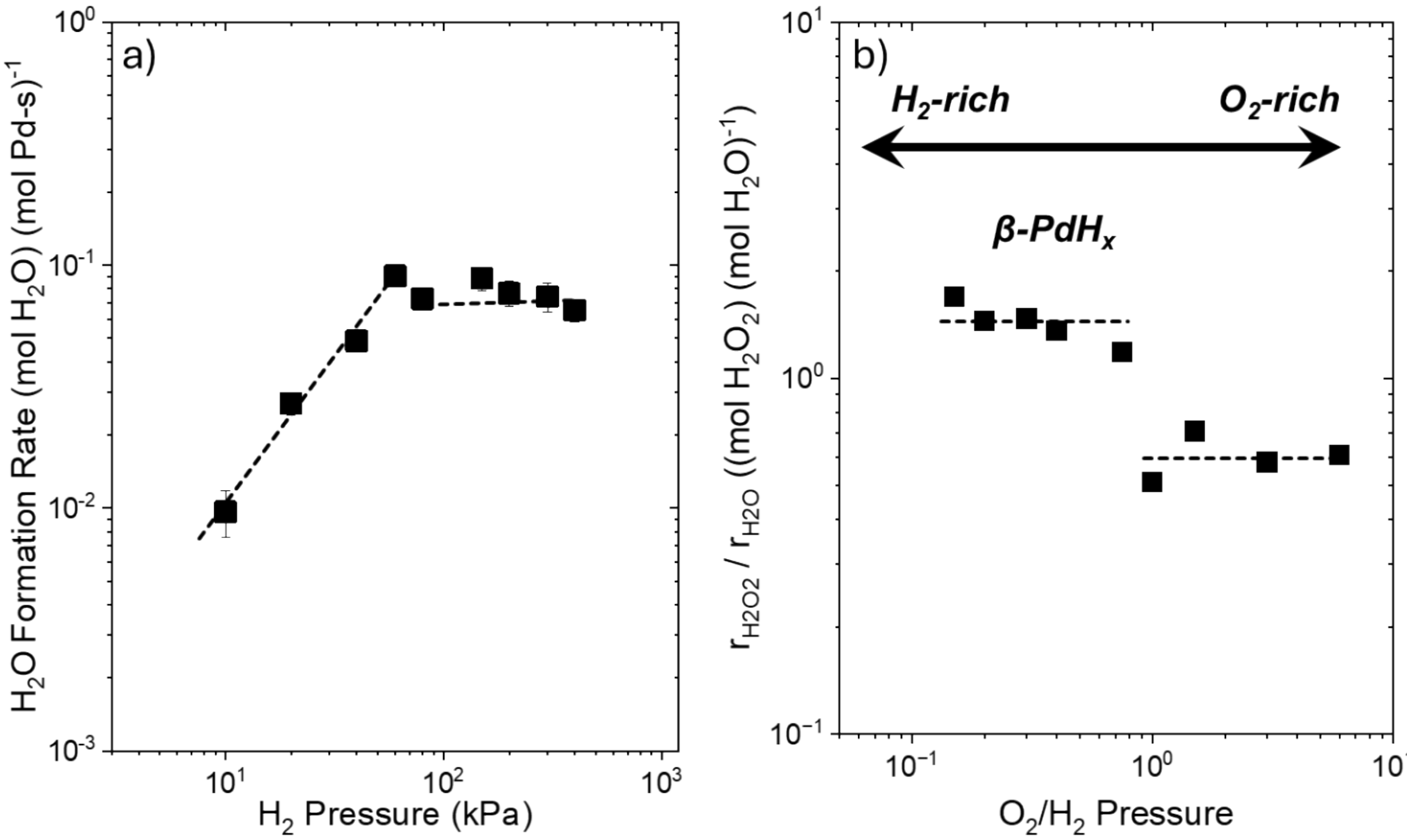

Figure S14. Steady-state (a) H_2O formation rates as a function of H_2 pressure and (b) rate ratio of H_2O_2 and H_2O formation rates as a function of ratio of O_2 to H_2 partial pressures for 2 nm Pd-SiO_2 in the absence of CO_2 (10-400 kPa H_2, 60 kPa O_2, 278 K).

Figure S14a shows steady-state H_2O formation rates as a function of H_2 pressure for Pd-SiO_2. Similar to H_2O_2 rates, H_2O rates also increase linearly with H_2 pressure at H_2 pressure lower than 80 kPa and become constant at pressures greater than 100 kPa. H_2O rates follow rate expression derived in Section S3 in the absence of CO_2. Rate ratios of H_2O_2 and H_2O formation rates (or H_2O_2 selectivity) remain constant when O_2/H_2 pressures are greater than 0.8 (corresponding to 10-80 kPa H_2), show a step increase at O_2/H_2 pressure ratios less than 1 (corresponding to 100-400 kPa H_2), and remain constant for lower O_2/H_2 pressure ratios (Figure S14b). This discontinuity and increase in rate ratio of H_2O_2 and H_2O formation rates or selectivity at high H_2 pressures is due to the formation of a β-PdH_x phase in the absence of CO_2 as reported previously.[1]

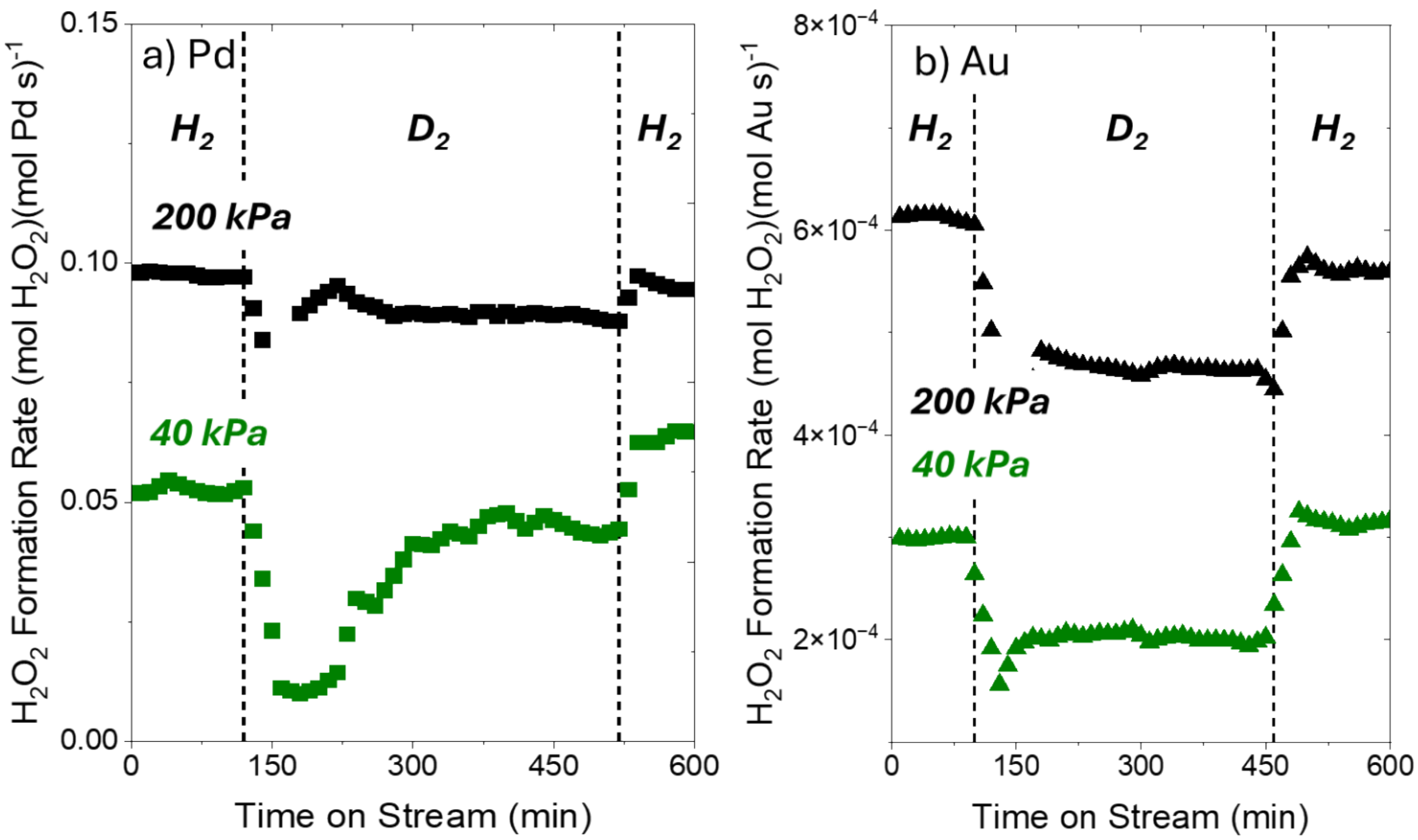

Figure S15. H_2O_2 formation rates as a function of time in water on (a) Pd and (b) Au nanoparticles in a trickle-bed reactor in the absence of CO_2 at 200 kPa H_2 or D_2 (black) and 40 kPa H_2 or D_2 (green) (60 kPa O_2 at 286 K).

Figure S15 shows the rates of H_2O_2 formation as a function of time on stream in a trickle bed reactor at H_2-rich (black; 200 kPa H_2, 60 kPa O_2) and O_2-rich (green; 40 kPa H_2, 60 kPa O_2) conditions on Pd and Au nanoparticles in response to changes in the identity of the hydrogen isotope (H_2 vs D_2). Here, H_2 is replaced with D_2 at ~120 minutes and returned to H_2 at ~530 minutes for Pd and ~460 minutes for Au. H_2O_2 formation rates in H_2 are greater than those with D_2 for both Pd and Au at H_2-rich and O_2-rich conditions in the absence of CO_2 (Figure 4), which agrees with H-H (or D-D) bond dissociation proceeding in a kinetically relevant fashion as shown in prior publications.[1, 2, 5, 6]

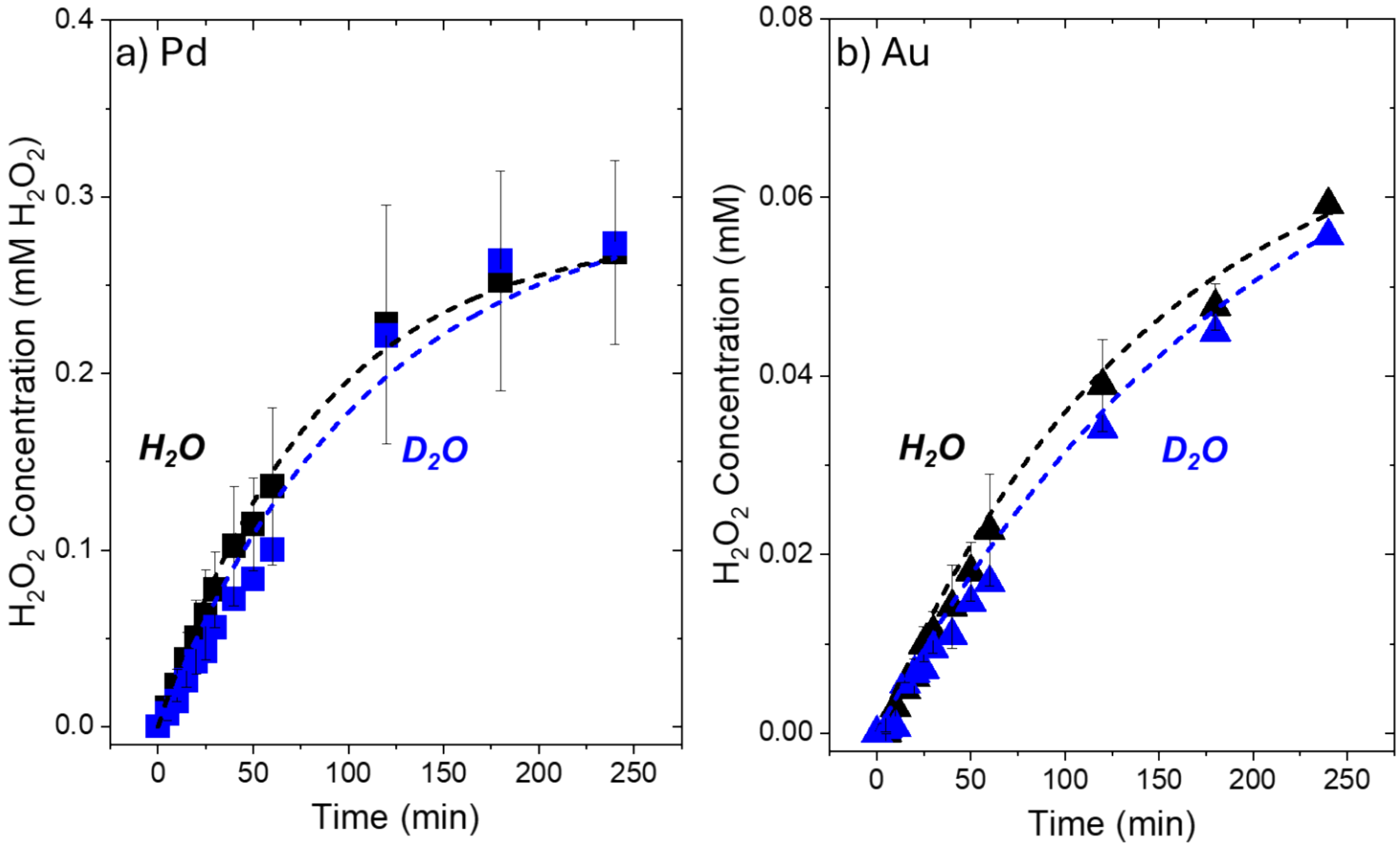

Figure S16. Cumulative H_2O_2 concentration over a period of 4 hours during direct synthesis when reacting H_2 with O_2 in water (black) or heavy water (blue) over (a) 50 mg of Pd-SiO_2, (b) 200 mg of Au-SiO_2 (4.8 kPa H_2, 4.8 kPa O_2, 80 cm^3 solvent, 298 K). Dashed lines fitted to equations and analyzed using methods reported in our prior work.[1, 2]

Figure S16 shows the transient concentration profiles of H_2O_2 formation in H_2O and D_2O solvents for Pd (S16a) and Au (S16b) nanoparticles. Comparisons of concentration profiles within H_2O and D_2O solvents show similar formation rates of peroxide within error for both Pd and Au in the absence of CO_2. This indicates that rates of peroxide formation and decomposition, and the apparent rate constants (k_1 and k_2) are similar within perhydrogenated and perdeuterated water (Table 2), and that proton transfer steps are not kinetically relevant for the primary formation and secondary decomposition of peroxide on Pd and Au in the absence of CO_2.

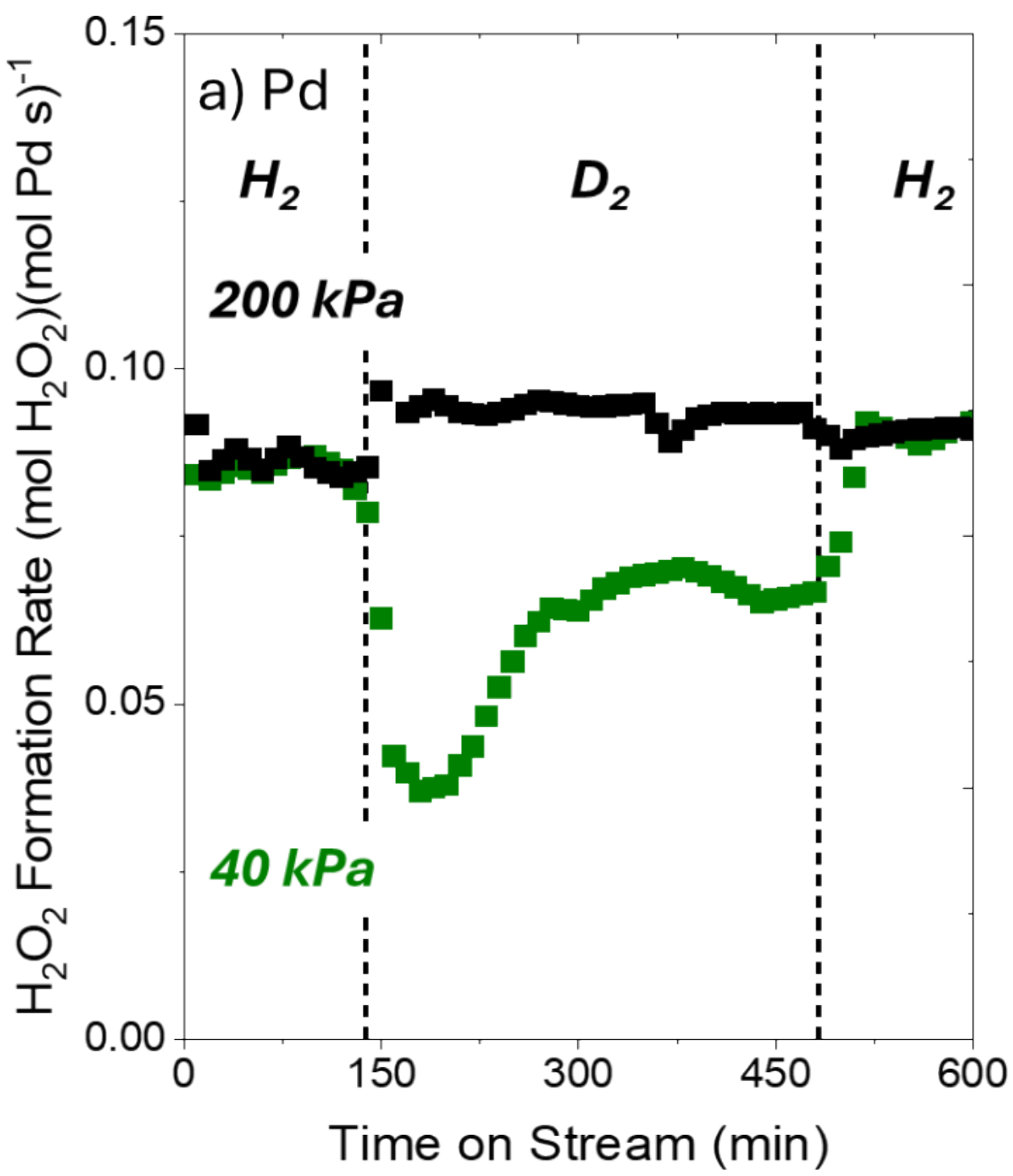

Figure S17. H_2O_2 formation rates as a function of time in water on (a) Pd and (b) Au nanoparticles in a trickle-bed reactor in the presence of CO_2 at 200 kPa H_2 or D_2 (black) and 40 kPa H_2 or D_2 (green) (60 kPa O_2, 1000 kPa CO_2 at 286 K).

Figure S17 shows the rates of H_2O_2 formation as a function of time on stream in a trickle bed reactor at H_2-rich (black; 200 kPa H_2, 60 kPa O_2) and O_2-rich (green; 40 kPa H_2, 60 kPa O_2) conditions on Pd and Au nanoparticles in the presence of CO_2 (1000 kPa) in response to changes in the identity of the hydrogen isotope (H_2 vs D_2). Here, H_2 is replaced with D_2 at ~130 minutes and returned to H_2 at ~470 minutes for Pd and ~400 minutes for Au. H_2O_2 formation rates in H_2 are greater than those with D_2 for both Pd and Au at O_2-rich conditions even in the presence of CO_2 (Figure 4), suggesting H-H (or D-D) bond dissociation proceeds in a kinetically relevant fashion. However, H_2O_2 formation rates in H_2 are lower than those with D_2 for both Pd and Au in H_2-rich conditions in the presence of CO_2 (200 kPa H_2 or D_2, 1000 kPa CO_2). This suggests that an increase in hydrogen (or deuterium) coverage in the presence of CO_2 and additional reaction pathways introduced by CO_2 alter the kinetic relevance of H-H bond dissociation such that there is a transition from a normal kinetic isotope effect to a regime influenced by equilibrium isotope effects.

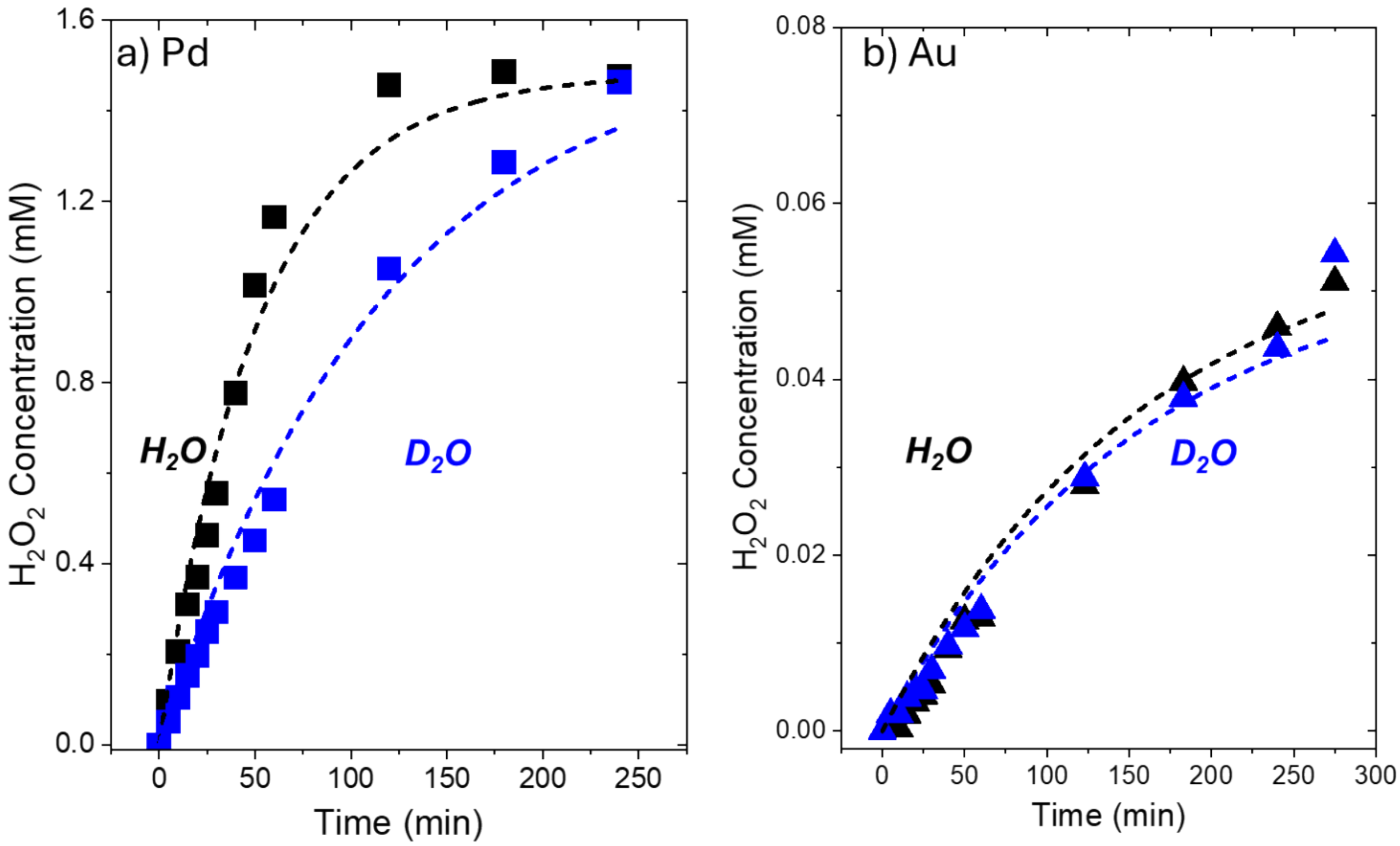

Figure S18. Cumulative H_2O_2 concentration over a period of 4 hours during direct synthesis when reacting H_2 with O_2 in water (black) or heavy water (blue) in the presence of CO_2 over (a) 50 mg of Pd-SiO_2, (b) 200 mg of Au-SiO_2 (4.8 kPa H_2, 4.8 kPa O_2, 91.4 kPa CO_2, 80 cm^3 solvent, 298 K). Dashed lines fitted to equations and analyzed using methods reported in our prior work.[1, 2]

Figure S18 shows the transient concentration profiles of H_2O_2 formation in H_2O and D_2O solvents for Pd (S18a) and Au (S18b) nanoparticles in the presence of CO_2. Comparisons of concentration profiles in H_2O and D_2O solvents for Au show similar peroxide formation rates within error. This indicates rates of peroxide formation and decomposition and apparent rate constants (k_1 and k_2) are similar within perhydrogenated and perdeuterated water (Table 2) and that proton transfer steps are not kinetically relevant for the primary formation and secondary decomposition of peroxide. However, concentration profiles for H_2O_2 within D_2O are lower than those within H_2O for Pd. Furthermore, the rates and apparent rate constants for peroxide formation (k_1) and decomposition (k_2) are higher in perhydrogenated than in perdeuterated water with CO_2 for Pd catalysts. This confirms that the addition of CO_2 makes proton transfer steps kinetically relevant by mediating these steps on Pd (Scheme S2).

S5. Supplementary Characterization and Kinetics Validating CO_2-Derived Intermediates on Pd and Au

S5.1. *Operando* Infrared Spectra

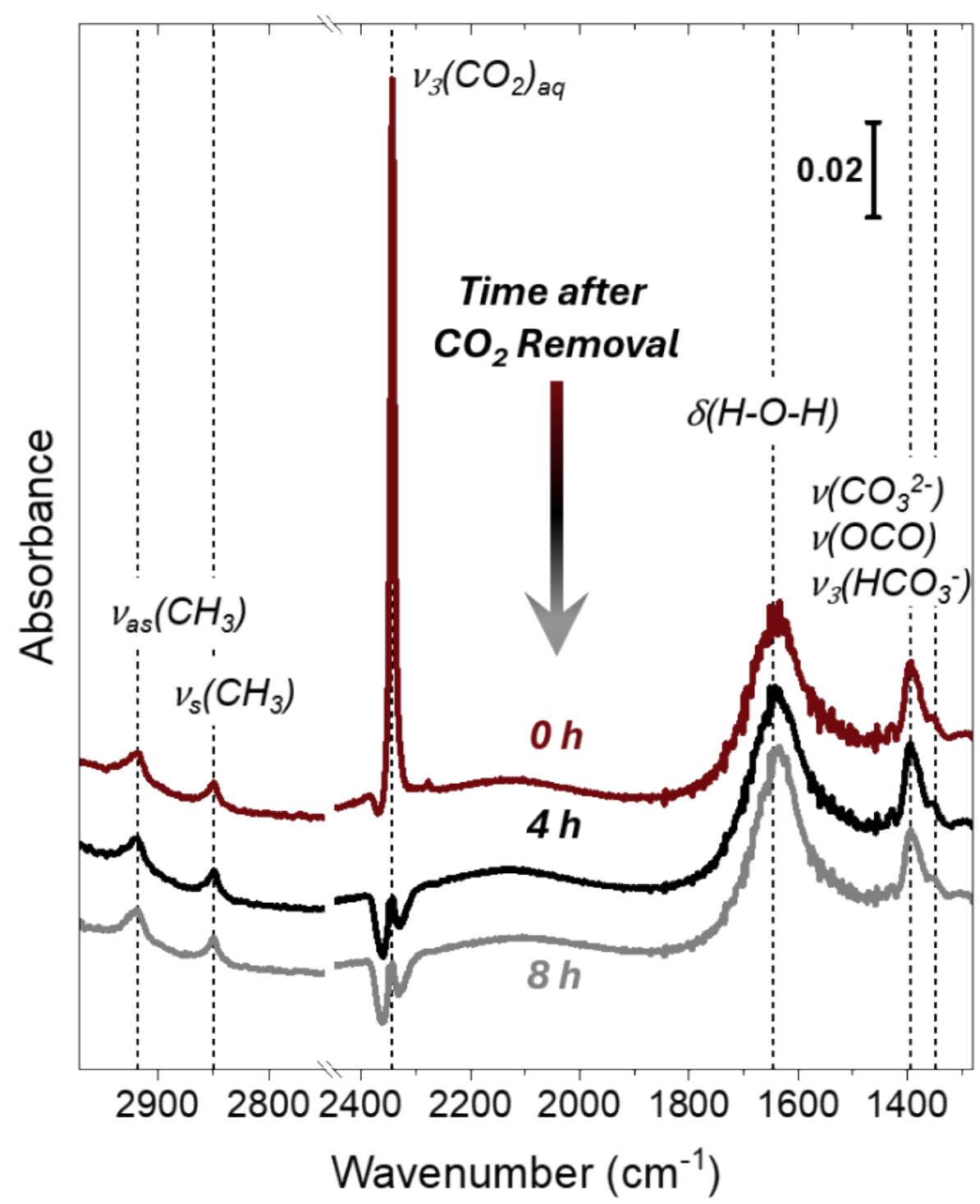

Figure S19. *Operando* infrared spectra of Pd-SiO_2 flushing with H_2:O_2:Ar gas saturated water post CO_2 exposure (200 kPa H_2, 60 kPa O_2, 0 kPa CO_2, 298 K) Dark red spectrum represents Pd sample 0 h after flushing, black represents 4 h, and grey 8 h after flushing with gas saturated liquid stream not containing CO_2.

Figure S19 shows *operando* infrared spectra of Pd nanoparticles following exposure to O_2 reduction conditions containing CO_2 shown in Figure 6 in the main text. Post CO_2 exposure, the gas stream was switched to containing H_2, O_2, and Ar (200 kPa H_2, 60 kPa O_2, 0 kPa CO_2). The spectra shown in Figure S19 shows that the features corresponding to adsorbed methyl groups ($\square_{as}(CH_3)$ at 2920 cm^{-1} and $\square_s(CH_3)$ at 2850 cm^{-1}), aqueous carbon dioxide ($\square(CO_2)_{aq}$ at 2343 cm^{-1}), hydroxyl groups ($\square$(HOH) at 1640 cm^{-1}), bicarbonate/carbonates and adsorbed formates ($\square$(OCO) or $\square_3(CO_3^{2-})$ at 1390 cm^{-1}, $\square_3(HCO_3^-)$ at 1354 cm^{-1})[7-9] remain after removing CO_2 from the gas stream for extended periods of time (~8 h or more), while the CO_2 feature at 2343 cm^{-1} disappears. This confirms that the species formed upon CO_2 exposure on Pd are strongly bound to the surfaces.

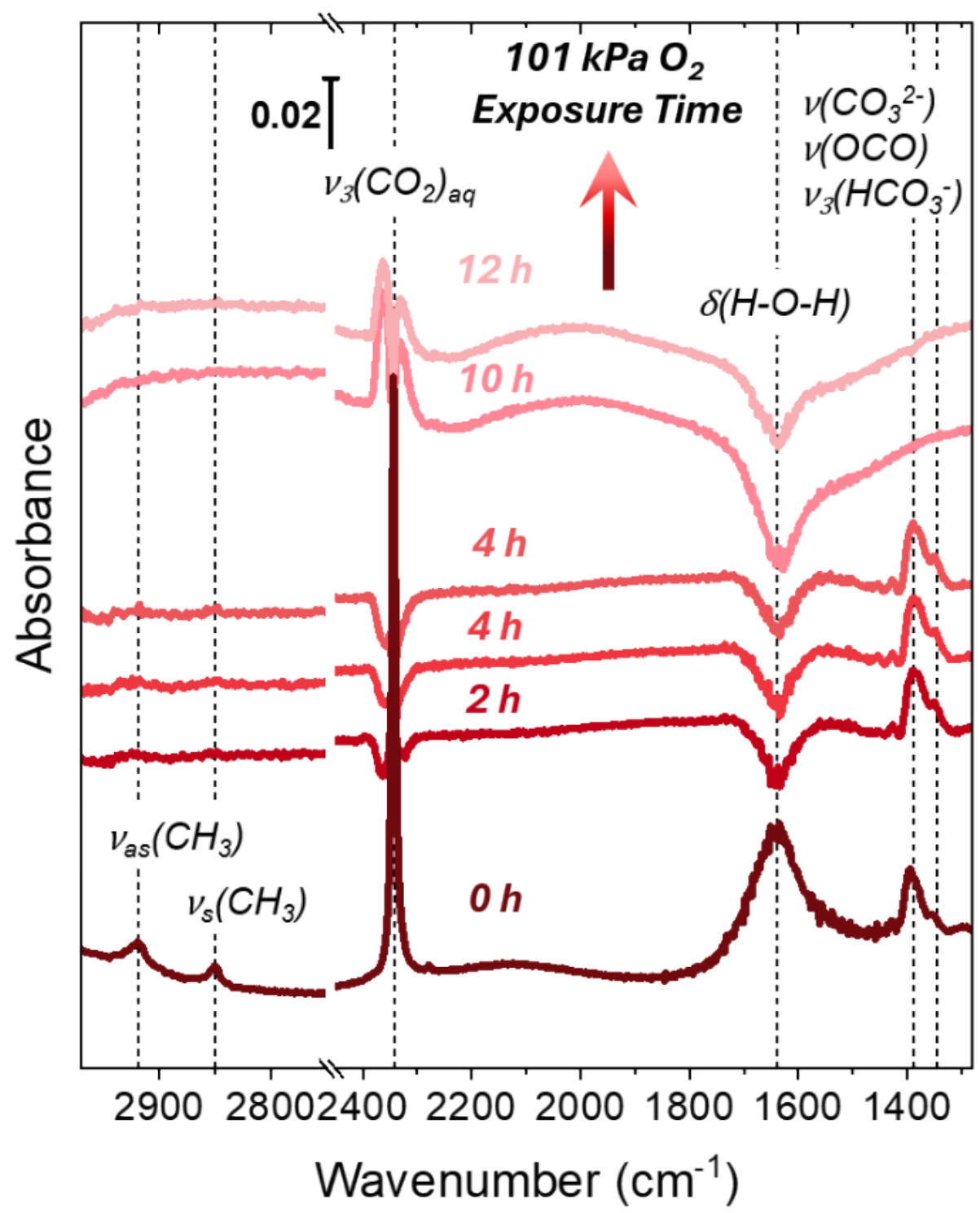

Figure S20. *In situ* infrared spectra of CO_2 exposed Pd-SiO_2 upon O_2 exposure over time (101 kPa O_2, 298 K, 12 h).

Figure S20 shows infrared spectra of Pd-SiO_2 catalyst exposed to gaseous O_2 (101 kPa O_2, 298 K) for 12 h post exposure to O_2 reduction reaction conditions with CO_2 shown in Figure 6 (Figure S20 brown spectrum). Upon exposure to O_2, the features corresponding to methyl groups, carbonates, and formates decrease and disappear over time. The carbonate and formate features persist for longer times than the methyl features, suggesting these are strongly adsorbed onto Pd surfaces.

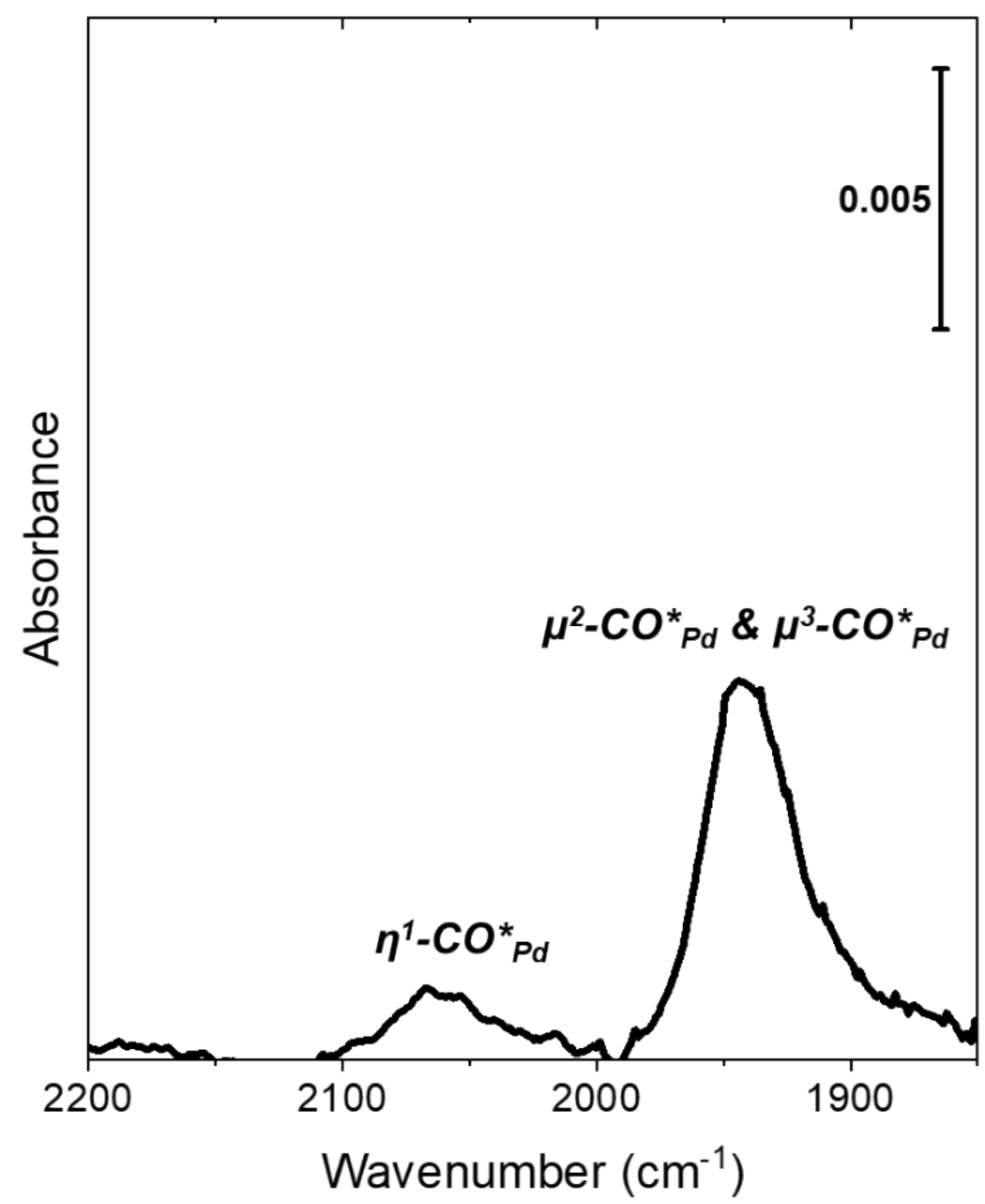

Figure S21. Infrared spectrum of Pd-SiO_2 in the ATR cell after CO exposure (1 kPa CO, 298 K).

Figure S21 shows the infrared spectrum of fresh Pd-SiO_2 catalyst exposed to CO (1 kPa, 298 K) prior to exposure to water and O_2 reduction reaction conditions. The spectrum shows features at ~2130 cm^{-1} corresponding to CO adsorbed linearly atop Pd atoms (η^1-CO^*_{Pd}), and multiply bound CO at two- (μ^2-CO^*_{Pd}) and three-fold sites (μ^3-CO^*_{Pd}) at wavenumbers below 2000 cm^{-1}. This confirms the presence of Pd nanoparticles deposited on the ATR-IR crystal and the position of peaks for CO adsorbed to Pd, which are not present when O_2 reduction reaction conditions with CO_2 are introduced to Pd (Figure 6).

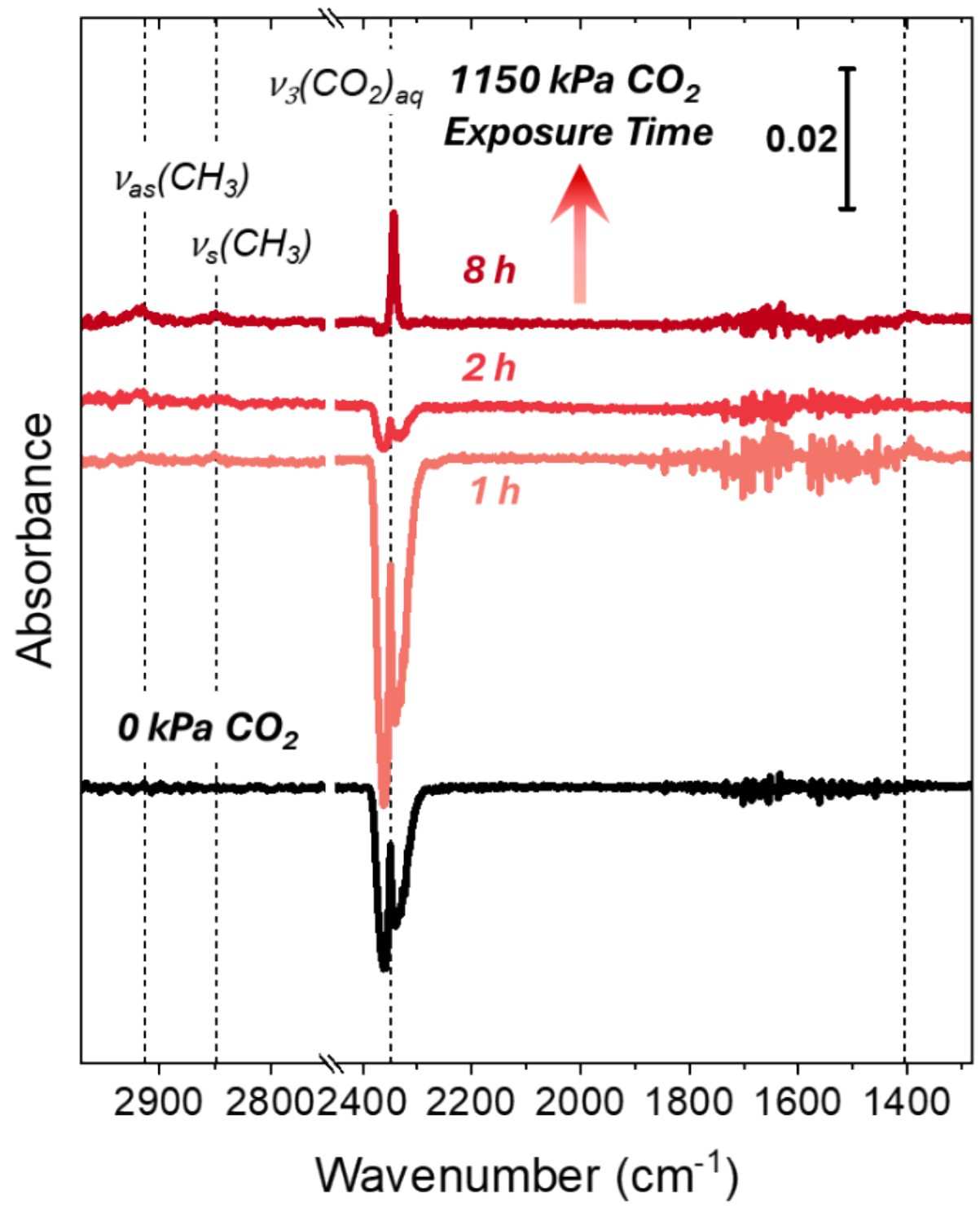

Figure S22. *Operando* infrared spectra of Au-SiO_2 upon CO_2 exposure over time under O_2 reduction reaction conditions for 8 h (200 kPa H_2, 60 kPa O_2, 1150 kPa CO_2, 298 K). Black spectrum represents O_2 reduction reaction conditions in the absence of CO_2 (200 kPa H_2, 60 kPa O_2, 1150 kPa Ar, 298 K)

Figure S22 shows *operando* ATR-IR spectra of Au nanoparticles following exposure to O_2 reduction conditions first without CO_2 (black) and with CO_2 over time (light to dark red – 8 h). Upon switching gas streams to that containing CO_2, H_2O_2 formation rates measured at the outlet of the infrared cell increase two-fold, consistent with trickly-bed reactor measurements (Figure 1), and a peak corresponding to aqueous CO_2 emerges (ν$(CO_2)_{aq}$ at 2343 cm^{-1}) and increases over time (2343 cm^{-1}). However, other features corresponding to formates and carbonates observed on Pd (Figures 6 and S19) are absent or very weak after 8 h of exposure. Additionally, features associated with CO adsorption on Au surfaces are also absent, as on Pd. These results confirm that CO_2-derived intermediates do not form and bind to Au surfaces or form in small quantities over longer periods of reaction time when compared to Pd.

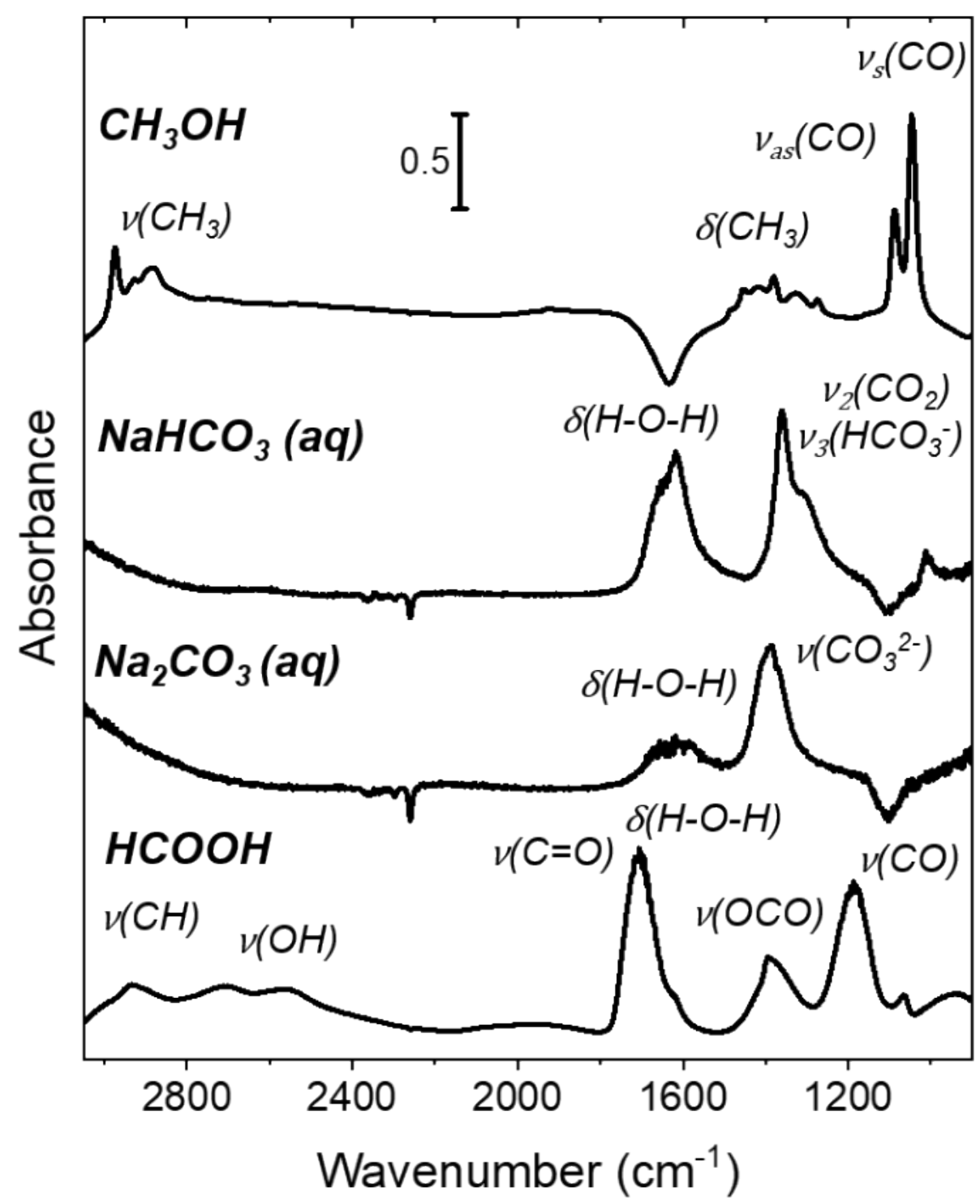

Figure S23. Infrared spectra of Ar-purged aqueous formic acid (1.3 M HCOOH), sodium carbonate (0.2 M Na_2CO_3), sodium bicarbonate (0.2 M $NaHCO_3$), and methanol (4.9 M CH_3OH) solutions over a blank ZnSe internal reflection element at 298 K.

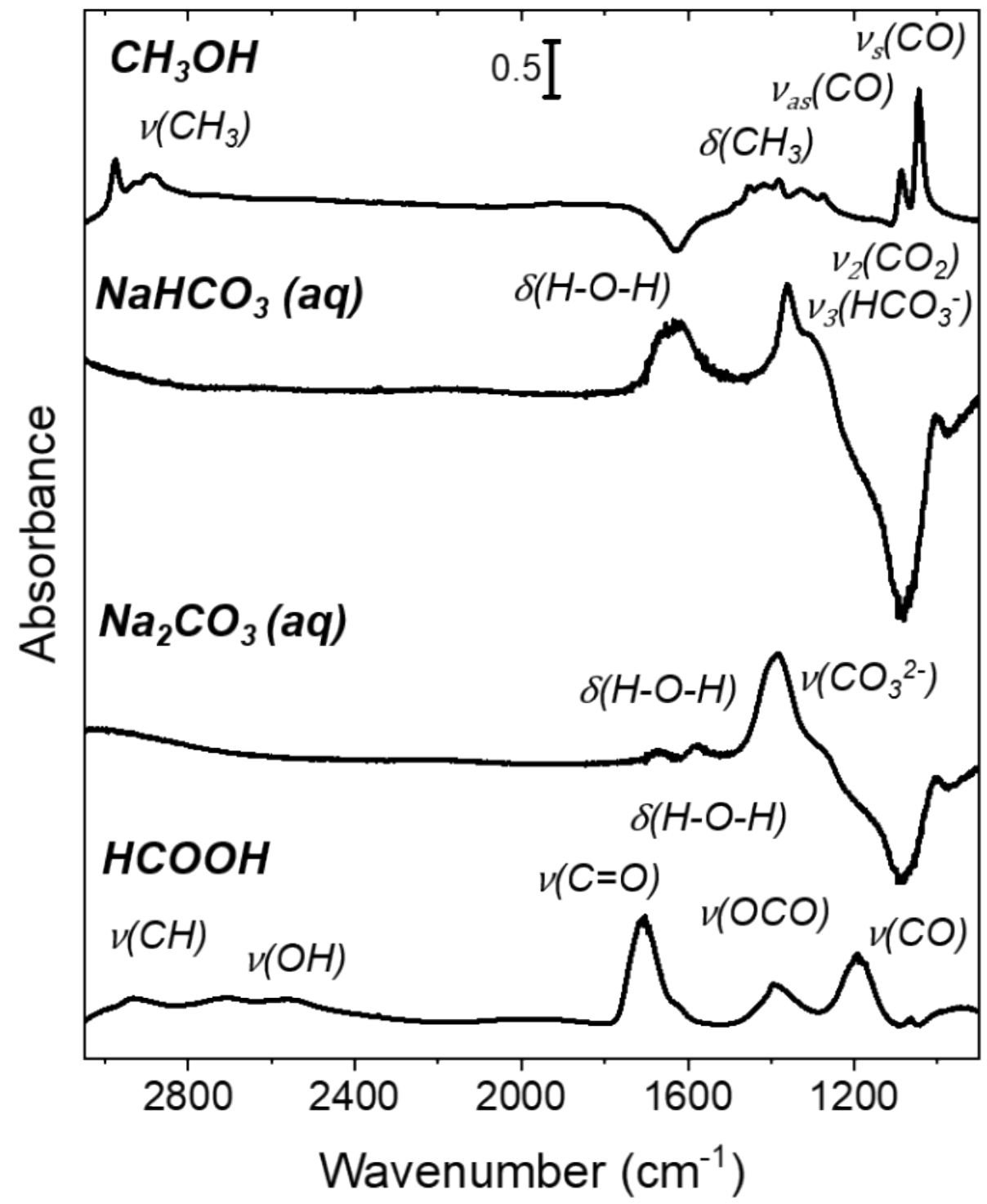

Figure S24. Infrared spectra of Ar-purged aqueous formic acid (1.3 M HCOOH), sodium carbonate (0.2 M Na_2CO_3), sodium bicarbonate (0.2 M $NaHCO_3$), and methanol (4.9 M CH_3OH) solutions over Pd-SiO_2 (298 K).

Figure S23 presents liquid phase infrared spectroscopy performed with a blank IRE loaded in the ATR-IR cell with formic acid, sodium carbonate, sodium bicarbonate, and methanol to assist with peak assignments for Figure 6 as these are common CO_2-reduction products. The same set of experiments were also conducted on the IRE coated with Pd-SiO_2 to further validate peak assignments and is presented in Figure S24. Peak assignments for the features in both figures were done following previously published works and are listed in Table S6 below.[7-12] The spectrum with methanol over Pd catalyst shows numerous features from 2850-2950 cm^{-1} and 1250-1450 cm^{-1} corresponding to the different stretching and bending modes of methyl groups [ν(CH_3), δ(CH_3)] respectively. Furthermore, the spectrum with under oxygen reduction reaction conditions evidenced the formation of surface methoxy (CH_3O^*) and hydroxymethyl (CH_2OH^*) species that act as redox mediators through the appearance of methylene [ν(CH_2) at 2780 cm^{-1} and δ(CH_2) at 1536 cm^{-1}] and carbon-oxygen bonds [ν(C-O) at 1150 cm^{-1}].[1, 12] The spectra shown in Figures 6, S20, and S21 of Pd catalysts exposed to oxygen reduction reaction conditions in the presence of CO_2 confirm the formation of methyl features, but no methoxy or hydroxymethyl features suggesting that the redox mediators formed in the presence of CO_2 differ from those formed in the presence of methanol. Furthermore, the lack of features around 1500-1600 cm^{-1} [ν(C=C)] confirms that aromatic organic species are not formed in the presence of CO_2.

The spectrum of aqueous sodium bicarbonate over Pd shows features associated with the vibrational stretching modes of C-O in bicarbonate at ~1309 and 1361 cm^{-1} [ν$_2$(CO_2), ν$_3$(HCO_3^-)] and the water bending mode between 1600 and 1650 cm^{-1} [δ(HOH)].[10] The spectrum of aqueous sodium carbonate over Pd shows C-O stretching modes associated with carbonates at higher wavenumbers than sodium bicarbonate at 1370 and 1385 cm^{-1} [ν$_3$(CO_3^{2-})].[10] The spectrum of formic acid over Pd shows peak corresponding to the vibrational stretching modes of C-O bonds in formic acid at ~1062 and 1195 cm^{-1} [ν$_s$(CO), ν$_{as}$(CO)], O-C-O bonds at ~1393 and 1627 cm^{-1} [ν$_s$(OCO), ν$_{as}$(OCO)], C=O bonds at 1705 cm^{-1} [ν(C=O)], O-H bonds between 2550 and 2710 cm^{-1} [ν(OH)], and C-H bonds at ~ 2939 cm^{-1} [ν(CH)].[11] Comparisons between these spectra and those shown in Figures 6, S20, and S21 of Pd catalysts exposed to oxygen reduction reaction conditions in the presence of CO_2 suggests a combination of features corresponding to methyl groups [ν(CH_3) at 2850 and 2920 cm^{-1}], and features that could be attributed to either carbonates, bicarbonates, formic acid/formates, or a combination of these species [ν$_3$(CO_3^{2-}) from carbonate or ν(OCO) from formic acid at 1390 cm^{-1}, and ν$_3$(HCO_3^-) from bicarbonate at 1354 cm^{-1}] are present under reaction conditions that act as mediators to selectively form H_2O_2. Scheme S2 outlines a plausible mechanism of redox mediation on Pd through formate/formic acid-like intermediates formed from CO_2 under oxygen reduction reaction conditions.

Table S6. Infrared spectroscopy peak assignments and vibrational modes for features observed on Pd catalysts when exposed to methanol, sodium bicarbonate, sodium carbonate, formic acid, and oxygen reduction reaction conditions involving CO_2.

Peak assignment	CH_3OH[1,12] (cm^{-1})	$NaHCO_3$[10] (cm^{-1})	Na_2CO_3[10] (cm^{-1})	HCOOH[11] (cm^{-1})	Pd-SiO_2 Fig. 6, S20, S21 (cm^{-1})	Pd-SiO_2[1] CH_3OH*, CH_3O*, CH_2OH* (cm^{-1})
ν$_{s}$(CO)<br>ν$_{as}$(CO)	1045<br>1088			1062<br>1195		1150
δ(CH_3)	1273<br>1325<br>1380<br>1419<br>1456					1390
δ(CH_2)						1536
ν$_3$(CO_3^{2-})			1370<br>1385		1390	
ν$_{s}$(OCO)<br>ν$_{as}$(OCO)				1393<br>1627	1390	
ν$_2$(CO_2)		1309				
ν$_3$(HCO_3^{-})		1361			1354	
δ(HOH)		1600-1650	1580		1640	
δ(HOH)<br>ν$_1$ + ν$_4$(CO_3^{2-})			1718			
ν(C=O)				1705		
ν(OH)				2564<br>2705		3200
ν(CH_3)	2888<br>2925<br>2975				2850<br>2920	2855-2960
ν(CH_2)						2780

□(CH)				2939		

S5.2. Comparison of Apparent Activation Enthalpies

Section S3 describes the rate expressions for H_2O_2 and H_2O formation rates on Pd and Au nanoparticles at H_2-rich and O_2-rich conditions. Transition state theory can be applied to describe the relationship between the ratio of H_2O_2 and H_2O formation rates and the free energy differences between the apparent activation free energies. Under both H_2- rich and O_2-rich regimes, the rate ratios simplify to,

$$\frac{r_{H_2O_2}}{r_{H_2O}} = \left(\frac{k_4}{2k_5}\right) \qquad (S5.1)$$

Which can be written in terms of apparent free energy or apparent activation enthalpies and entropies using transition state theory,

$$\frac{r_{H_2O_2}}{r_{H_2O}} = \frac{k_4}{2k_5} = \frac{1}{2}\frac{e^{-\frac{\Delta G_4^\ddagger}{RT}}}{e^{-\frac{\Delta G_5^\ddagger}{RT}}} = \frac{1}{2}e^{\frac{\left(\Delta S_4^\ddagger - \Delta S_5^\ddagger\right)}{R}} \square\, e^{\frac{\left(\Delta H_5^\ddagger - \Delta H_4^\ddagger\right)}{RT}} \qquad (S5.2)$$

which implies that the difference between $\Delta H^\ddagger_{H_2O_2}$ and $\Delta H^\ddagger_{H_2O}$ equals the difference between the intrinsic barriers of Step 4 ($\Delta H_4^\ddagger$) and Step 5 ($\Delta H_5^\ddagger$).

$$\Delta\Delta H^\ddagger = \Delta H^\ddagger_{H_2O} - \Delta H^\ddagger_{H_2O_2} = \Delta H_5^\ddagger - \Delta H_4^\ddagger \qquad (S5.3)$$

Similar forms of expression can be used to describe the rate of 2 consumption, which at high hydrogen coverages looks like,

$$-r_{H_2} = k_{H_2}[O_2] \square\, e^{-\frac{\Delta G^\ddagger_{H_2}}{RT}}[O_2] \;\square\; e^{-\frac{\Delta H^\ddagger_{H_2}}{RT}}[O_2] \qquad (S5.4)$$

where the sum of H_2O_2 and H_2O formation rates gives the apparent rate constant of H_2 activation (k_{H_2}) which reflects the activation enthalpy for H_2 consumption ($\Delta H^\ddagger_{H_2}$).

Table S7. H_2O_2 Selectivity and Apparent activation enthalpy values ($\square H^{\ddagger}$) for H_2O_2 and H_2O formation, and H_2 activation on Pd at different H_2 and CO_2 pressures (60 kPa O_2, 278-308 K).

H_2 Pressure (kPa)	CO_2 Pressure (kPa)	H_2O_2 Selectivity (%)	$\square H^{\ddagger}_{H_2O_2}$ (kJ mol^{-1})	$\square H^{\ddagger}_{H_2O}$ (kJ mol^{-1})	$\square H^{\ddagger}_{H_2}$ (kJ mol^{-1})
40	0	22	19.1 ± 2.0	11.3 ± 2.2	15.4 ± 1.5
	1150	41	13.1 ± 0.5	35.0 ± 4.9	22.1 ± 2.1
200	0	55	11.0 ± 2.0	18.1 ± 2.7	15.1 ± 1.0
	1150	90	15.1 ± 5.0	67.2 ± 5.8	33.4 ± 0.4

Apparent activation enthalpies for H_2O_2 and H_2O formation, and H_2 activation at different H_2 and CO_2 pressures for Pd is shown in Table S6. Under O_2-rich conditions, values of $\square H^{\ddagger}_{H_2O_2}$ are lower in the presence of CO_2 while $\square H^{\ddagger}_{H_2O}$ and $\square H^{\ddagger}_{H_2}$ are higher when CO_2 is added to the reaction. At H_2-rich conditions, values for $\square H^{\ddagger}_{H_2O_2}$, $\square H^{\ddagger}_{H_2O}$, and $\square H^{\ddagger}_{H_2}$ all increase in the presence of CO_2. Under both conditions, the values for $\square H^{\ddagger}_{H_2O}$ responsible for H_2O formation which involves steps that cleave O-O bonds increase remarkedly compared to H_2O_2 formation and H_2 activation. The differences in apparent activation enthalpies in the presence and absence of CO_2 reflect the increase in H_2O_2 selectivity observed in the presence of CO_2 under both conditions. This increase in all apparent enthalpies with CO_2 could suggest that CO_2-derived organic species bind to Pd surfaces and disrupt or block contiguous Pd ensembles which destabilize transition states and product states for reactions that cleave O–O bonds.

Table S8. H_2O_2 Selectivity and Apparent activation enthalpy values ($\Delta H^{\ddagger}$) for H_2O_2 and H_2O formation, and H_2 oxidation on Au (60 kPa O_2, 278-308 K).

**H_2 Pressure (kPa)**	**CO_2 Pressure (kPa)**	**H_2O_2 Selectivity (%)**	**$\Delta H^{\ddagger}_{H_2O_2}$ (kJ mol^{-1})**	**$\Delta H^{\ddagger}_{H_2O}$ (kJ mol^{-1})**	**$\Delta H^{\ddagger}_{H_2}$ (kJ mol^{-1})**
200	0	85	25 ± 2	74 ± 5	30 ± 4
	1150	93	16 ± 1	105 ± 15	37 ± 1

Table S8 shows apparent activation enthalpies for H_2O_2 and H_2O formation, and H_2 activation at H_2-rich conditions for Au nanoparticles at different CO_2 pressures. Similar to the trends observed for Pd under conditions where catalyst surfaces are not saturated with hydrogen, values of $\Delta H^{\ddagger}_{H_2O_2}$ are lower in the presence of CO_2 while $\Delta H^{\ddagger}_{H_2O}$ and $\Delta H^{\ddagger}_{H_2}$ are higher when CO_2 is added to the reaction. These trends coincide with increases in H_2O_2 rates and selectivities observed for Au and suggest CO_2 derived intermediates destabilize transition states for H_2O formation.

S5.3. Temperature Programmed Oxidation of Catalysts Exposed to Reaction Conditions

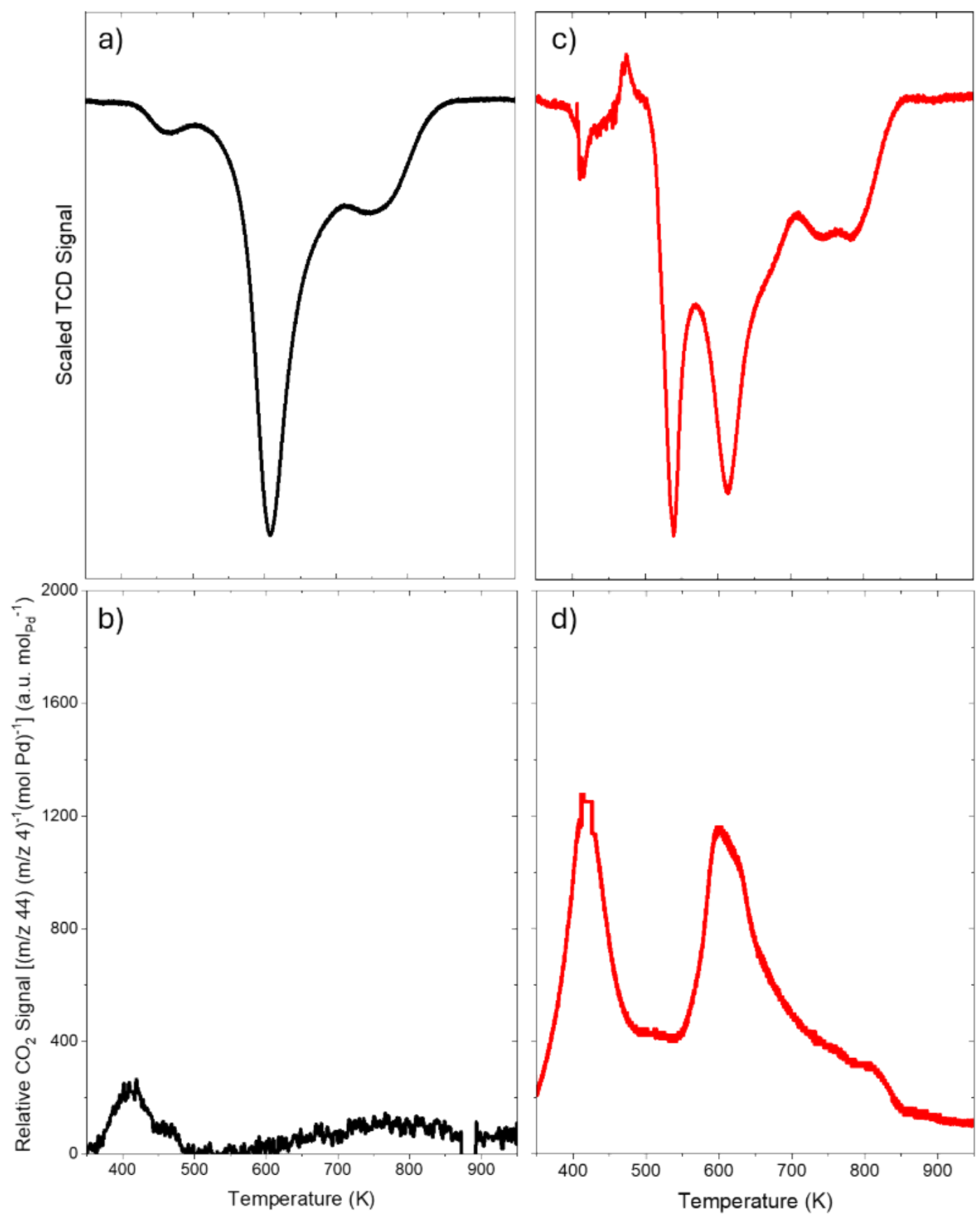

Figure S25. Temperature programmed oxidation (5 kPa O_2, 96 kPa He) thermal conductivity detector and mass spectrometry signals on Pd-SiO_2 catalysts post exposure to 24 h in the trickle-bed reactor following reactions with O_2 and H_2 in water (a, b) in the absence of CO_2 and (c, d) in the presence of CO_2 (200 kPa H_2, 60 kPa O_2, 0 or 1129 kPa CO_2, 278 K).

Figures S25a and S25c show the thermal conductivity detector (TCD) signals, scaled to the Pd oxidation peak at ~573 K, for Pd catalysts after exposure to O_2 reduction reaction conditions for 24 h, in the absence and presence of CO_2, respectively. Figures S25b and S25d show mass spectrometer (MS) signals for CO_2 (m/z = 44) relative to He (m/z = 4) normalized by moles of active metal corresponding to the TCD signals shown in S25a and S25c, respectively. The strong negative peaks in the TCD signal in S25a between 500 and 800 K for Pd catalysts not exposed to CO_2 correspond to O_2 consumption by Pd to oxidize metallic Pd to PdO. This also corresponds to CO_2 not being released during this process, as evidenced by the absence of an MS signal for CO_2 in this temperature range (Fig. S25c). When Pd is exposed to CO_2, the TCD signal between 500 and 800 K shows additional peaks corresponding to O_2 consumption (Fig. S25b), when compared with the sample not exposed to CO_2. The TCD signal also corresponds to CO_2 evolution observed between 500 and 800 K from the MS data (Fig. S25d). Together, these comparisons confirm that, in addition to oxidizing Pd to PdO, O_2 is further consumed to oxidize

adsorbed organics from the Pd surface when Pd is exposed to thermochemical oxygen reduction reaction conditions with CO_2. This confirms the presence of strongly bound CO_2-derived organic intermediates on Pd surfaces that are formed *in situ* during O_2 reduction reactions.

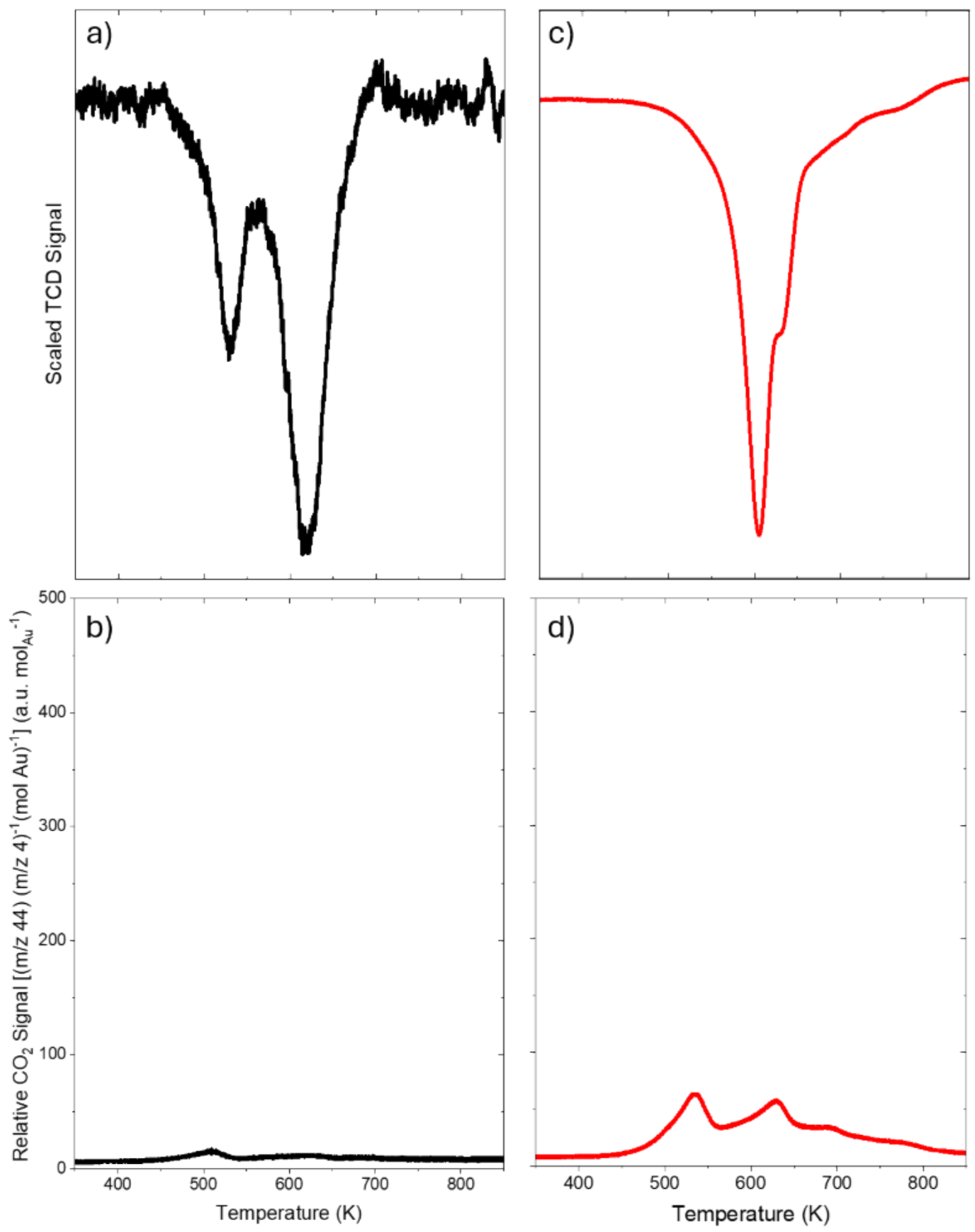

Figure S26. Temperature programmed oxidation (5 kPa O_2, 96 kPa He) thermal conductivity detector and mass spectrometry signals on Au-SiO_2 catalysts post exposure to 24 h in the trickle-bed reactor following reactions with O_2 and H_2 in water (a, b) in the absence of CO_2 and (c, d) in the presence of CO_2 (200 kPa H_2, 60 kPa O_2, 0 or 1129 kPa CO_2, 278 K).

Figures S26a and S26c show the thermal conductivity detector (TCD) signals, scaled to the prominent Au oxidation peak from 500 to 700 K for Au catalysts after exposure to O_2 reduction reaction conditions for 24 h, in the absence and presence of CO_2, respectively. Figures S26b and S26d show mass spectrometer (MS) signals for CO_2 (m/z = 44) relative to He (m/z = 4) normalized by moles of active metal corresponding to the TCD signals shown in S26a and S26c, respectively. The strong negative peaks in the TCD signal in S26a between 500 and 700 K for Au catalysts not exposed to CO_2 correspond to O_2 consumption by Au to oxidize metallic Au. This also corresponds to CO_2 not being released during this process, as evidenced by the absence of an MS signal for CO_2 in this temperature range (Fig. S26c). When Au is exposed to CO_2, the TCD signal between 500 and 700 K does not show any additional peaks corresponding to O_2 consumption (Fig. S26b), when compared with the sample not

exposed to CO_2. However, the onset of Au oxidation when Au is exposed to CO_2 shifts to higher temperatures than in the sample not exposed to CO_2. The corresponding MS signal for CO_2 shows that only small amounts of CO_2 are evolved in this temperature range (10-fold smaller than Pd). This could suggest that negligible quantities of CO_2-derived intermediates are adsorbed onto Au surfaces when Au catalysts are exposed to O_2 reduction reaction conditions in the presence of CO_2 for long periods, and that Au does not bind CO_2-derived intermediates as strongly as Pd.

S6. Detailed Analysis of Coordination Numbers, Bond Distances, and Catalyst Structure from *Operando* X-Ray Absorption Spectra

Table S9. Best-fit Pd K edge EXAFS Parameters for the *operando* measurements under different conditions for Pd-SiO_2 catalyst, the first three shells and multiple scattering are included[a].

Scan Conditions	Coordination shell Pd-Pd	Coordination number (-)	Bond disorder σ^2 × 10^3 (Å^2)	Bond length (Å)
In situ reduced, under He	1NN	10.7 ± 0.8	6.3 ± 0.5	2.756 ± 0.002
40 kPa H_2, 60 kPa O_2	1NN	10.3 ± 1.3	6.2 ± 0.9	2.749 ± 0.003
	2NN	6.6 ± 4.8	11 ± 6.0	3.870 ± 0.022
	3NN	11.6 ± 9.1	11 ± 6.0	4.744 ± 0.022
40 kPa H_2, 60 kPa O_2, 1150 kPa CO_2	1NN	9.2 ± 0.9	5.9 ± 0.7	2.744 ± 0.003
	2NN	5.9 ± 3.6	12 ± 5.3	3.865 ± 0.019
	3NN	9.9 ± 6.7	12 ± 5.3	4.740 ± 0.019
200 kPa H_2, 60 kPa O_2	1NN	9.9 ± 1.3	7.9 ± 1.0	2.823 ± 0.004
	2NN	3.9 ± 3.6	9.0 (set)	4.079 ± 0.070
	3NN	1.0 ± 3.3	7.5 (set)	4.812 ± 0.204
	1NN	9.1 ± 1.3	6.5 ± 1.0	2.754 ± 0.004

200 kPa H_2, 60 kPa O_2, 1150 kPa CO_2	2NN	6.5 ± 4.2	11 (set)	3.853 ± 0.027
	3NN	6.8 ± 4.6	10 (set)	4.727 ± 0.027

[a]The parameters for multiple scattering were not shown since the parameters are highly constrained.

Table S10. Comparison of Pd K edge *operando* EXAFS best fit results that includes only the first shell and three shells.

Scan Conditions	Coordination shell Pd-Pd	Coordination number (-)	Bond disorder σ² × 10³ (Å²)	Bond length (Å)
40 kPa H_2, 60 kPa O_2	1NN (fit 3 shells)	10.3 ± 1.3	6.2 ± 0.9	2.749 ± 0.003
	1NN (fit first shell only)	10.2 ± 1.2	6.2 ± 0.8	2.749 ± 0.003
40 kPa H_2, 60 kPa O_2, 1150 kPa CO_2	1NN (fit 3 shells)	9.2 ± 0.9	5.9 ± 0.8	2.744 ± 0.003
	1NN (fit first shell only)	9.0 ± 0.8	5.9 ± 0.7	2.743 ± 0.002
200 kPa H_2, 60 kPa O_2	1NN (fit 3 shells)	9.9 ± 1.3	7.8 ± 1.0	2.823 ± 0.004
	1NN (fit first shell only)	10.0 ± 1.3	7.9 ± 1.0	2.823 ± 0.004
200 kPa H_2, 60 kPa O_2, 1150 kPa CO_2	1NN (fit 3 shells)	9.1 ± 1.3	6.5 ± 1.0	2.754 ± 0.004
	1NN (fit first shell only)	8.7 ± 1.3	6.2 ± 1.0	2.754 ± 0.004

Table S11. Comparison of Au L edge *operando* EXAFS best fit results that includes the first three shells single scattering and multiple scattering[a].

Scan Conditions	Coordination shell Au-Au	Coordination number (-)	Bond disorder σ² × 10³ (Å²)	Bond length (Å)
40 kPa H_2, 60 kPa O_2	1NN	10.3 ± 0.9	8.4 ± 0.7	2.860 ± 0.003
	2NN	1.1 ± 1.5	2.3 ± 8.2	4.018 ± 0.026
	3NN	5.1 ± 1.9	6.5 (set)	4.950 ± 0.023
40 kPa H_2, 60 kPa O_2, 1150 kPa CO_2	1NN	9.9 ± 0.6	7.8 ± 0.5	2.858 ± 0.002
	2NN	2.3 ± 2.2	8.5 ± 7.7	4.029 ± 0.022
	3NN	6.3 ± 1.6	9.0 (set)	4.968 ± 0.017

[a]The parameters for multiple scattering were not shown since the parameters are highly constrained.

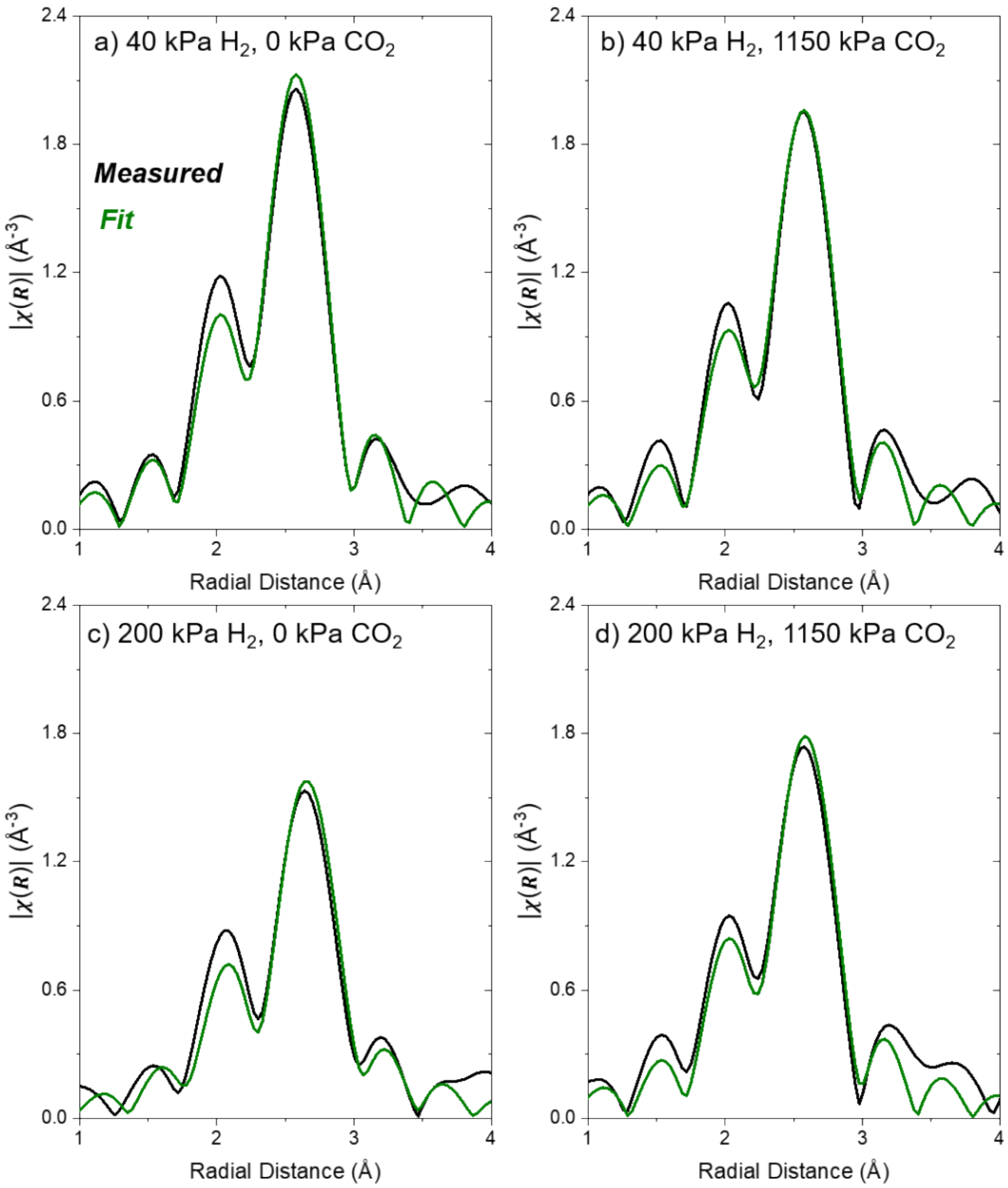

Figure S27. *Operando* k^2-weighted Pd K edge FT EXAFS (black) and fitted (green) spectra under O_2-rich conditions in the (a) absence and (b) presence of CO_2 (40 kPa H_2, 60 kPa O_2, 0 or 1150 kPa CO_2, 298 K). *Operando* k^2-weighted Pd K edge FT EXAFS spectra under H_2-rich conditions in the (c) absence and (d) presence of CO_2 (200 kPa H_2, 60 kPa O_2, 0 or 1150 kPa CO_2, 298 K). The fit includes only the first shell single scattering of Pd-Pd.

Figure S27 displays the fitted and measured Pd K edge FT EXAFS spectra for Pd-SiO_2 under O_2- and H_2-rich reaction conditions. The fitted spectra consider only the first shell Pd-Pd scattering. The fitted spectra between 2.3 and 3.5Å shows good agreement with the experimentally measured spectra, validating that contributions from only the Pd-Pd 1NN scattering are sufficient to describe the data. However, the fitted curve deviates from the experimentally measured spectra at ~2.0 Å, suggesting further contributions from light elements (e.g., O, C) that are adsorbed onto Pd under reaction conditions. This can be seen for the spectra obtained under both H_2- and O_2-rich conditions and in the presence of CO_2. The light elements were not included in the fits of the EXAFS spectra since the

compositions and exact identity of the surface species are unknown, and making assumptions could introduce artifacts, yielding an unrealistically low 1NN CN.

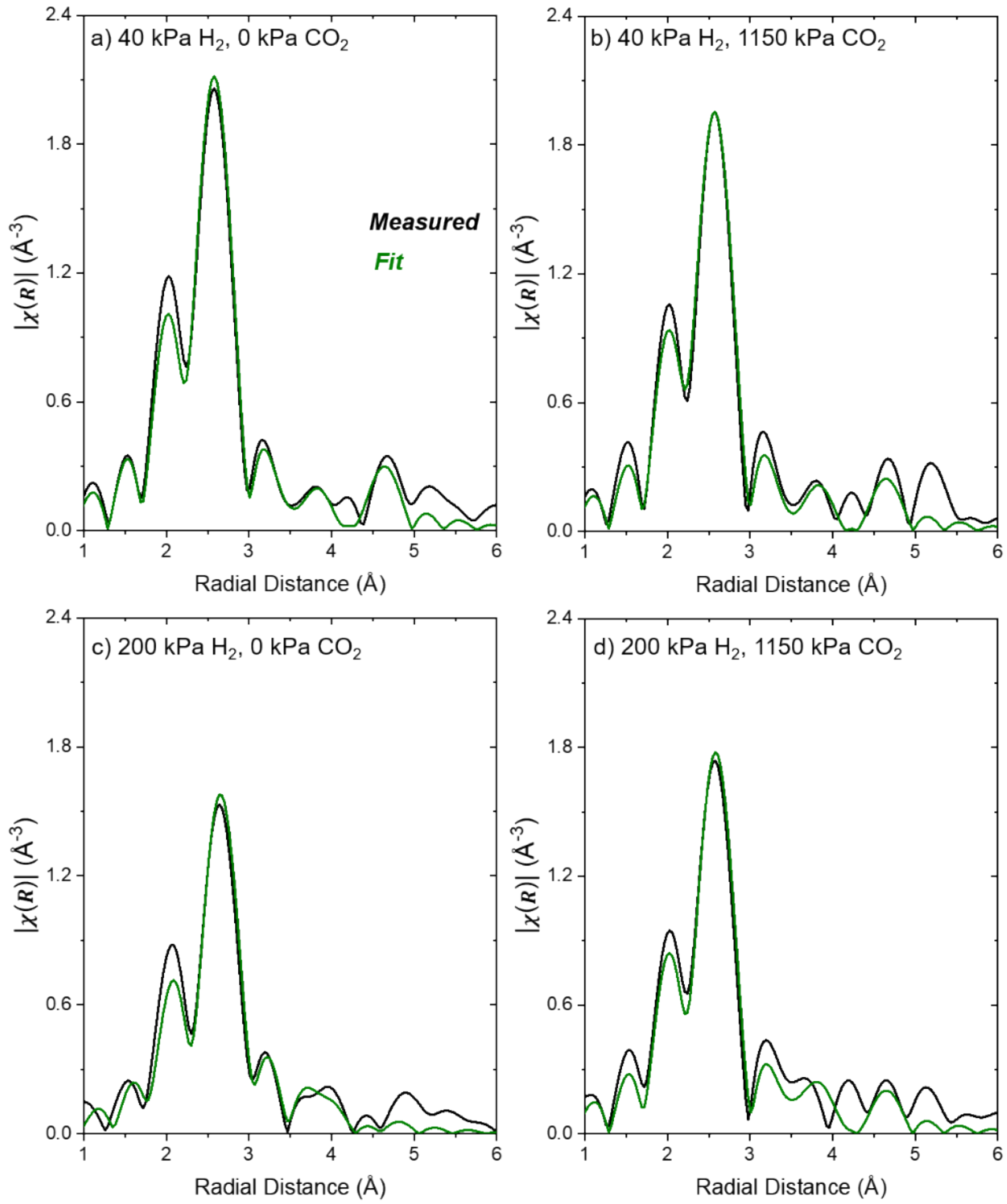

Figure S28. *Operando* k^2-weighted Pd K edge FT EXAFS (black) and fitted (green) spectra under O_2-rich conditions in the (a) absence and (b) presence of CO_2 (40 kPa H_2, 60 kPa O_2, 0 or 1150 kPa CO_2, 298 K). *Operando* k^2-weighted Pd K edge FT EXAFS spectra under H_2-rich conditions in the (c) absence and (d) presence of CO_2 (200 kPa H_2, 60 kPa O_2, 0 or 1150 kPa CO_2, 298 K). The fit includes the first three shell single scattering and multiple scattering of Pd-Pd.

Figure S28 shows the measured and fitted curve of Pd K edge FT EXAFS spectra for Pd-SiO_2 under O_2- and H_2-rich reaction conditions. The first three shells and multiple scattering of Pd-Pd are considered to investigate the effect of CO_2 on the shape of the nanoparticle. The quality of fit below a radial distance of 3.0 Å is similar to that of the results from Figure S27, indicating that the information obtained by fitting only the first shell single scattering of Pd-Pd accurately captures changes in the

nanoparticles. The contribution of the shells beyond the first shell is mainly observed between 3.5-4.8 Å.

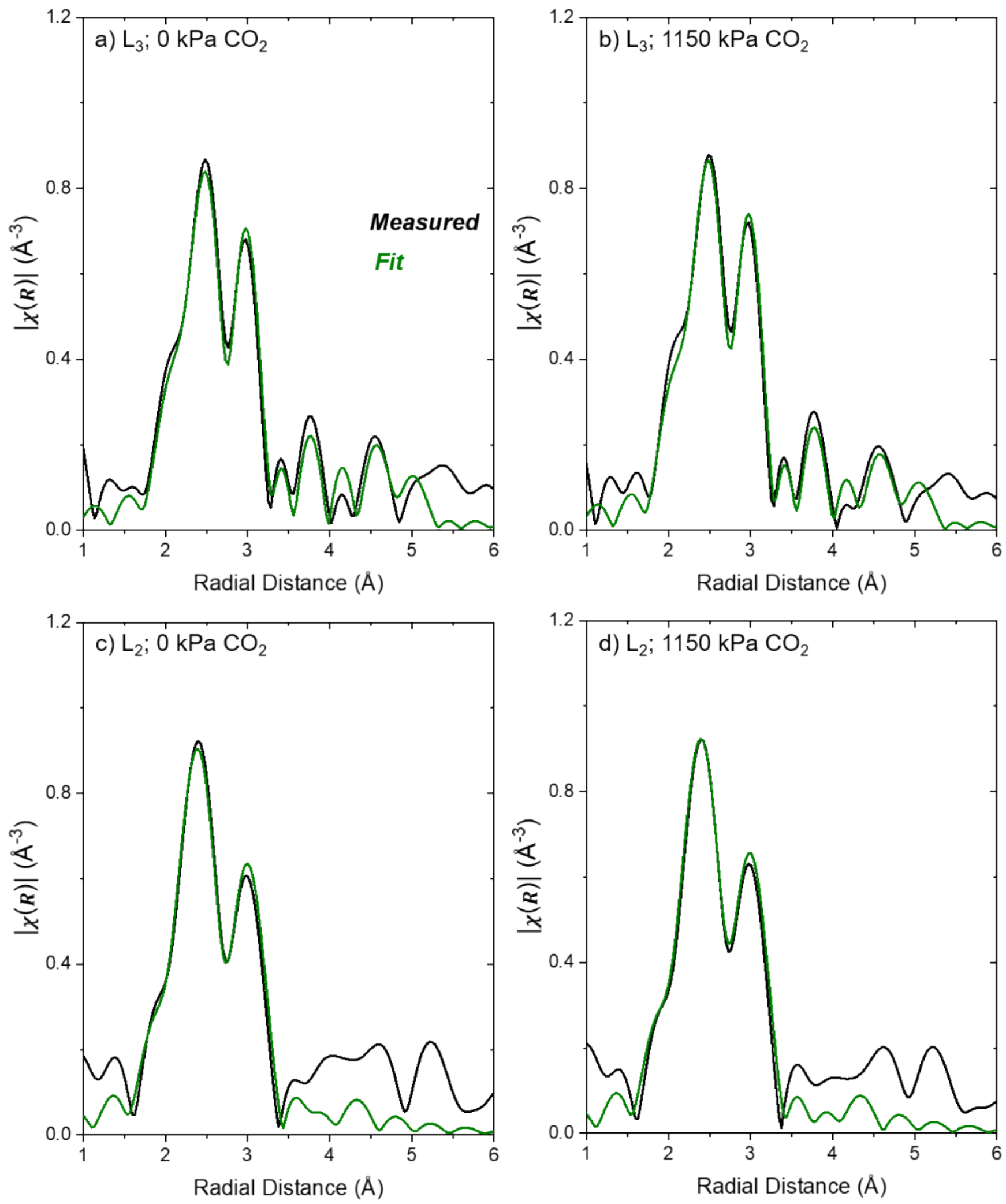

Figure S29. *Operando* k^2-weighted Au L_3 and L_2 edge FT EXAFS (black) and fitted (green) spectra under O_2-rich conditions in the (a, c) absence and (b, d) presence of CO_2 (40 kPa H_2, 60 kPa O_2, 0 or 1150 kPa CO_2, 298 K). The fit includes the first three shell single scattering and multiple scattering of Au-Au.

Figure S29 shows the measured and fitted curve of Au L edge FT EXAFS spectra for Au-SiO_2 under O_2-rich conditions in the absence and presence of CO_2. The contribution of 1NN Au-Au is between radial distance values 1.7 and 3.8 Å, while the contribution from higher shells is between 3.8 and 5.3 Å. The fitted spectra (green) shows good agreement with the experimental spectra (black) under all

conditions with and without CO_2 for Au nanoparticles, indicating that the Au-Au 1NN scattering is sufficient to describe the data and that contributions from light elements are negligible on Au.

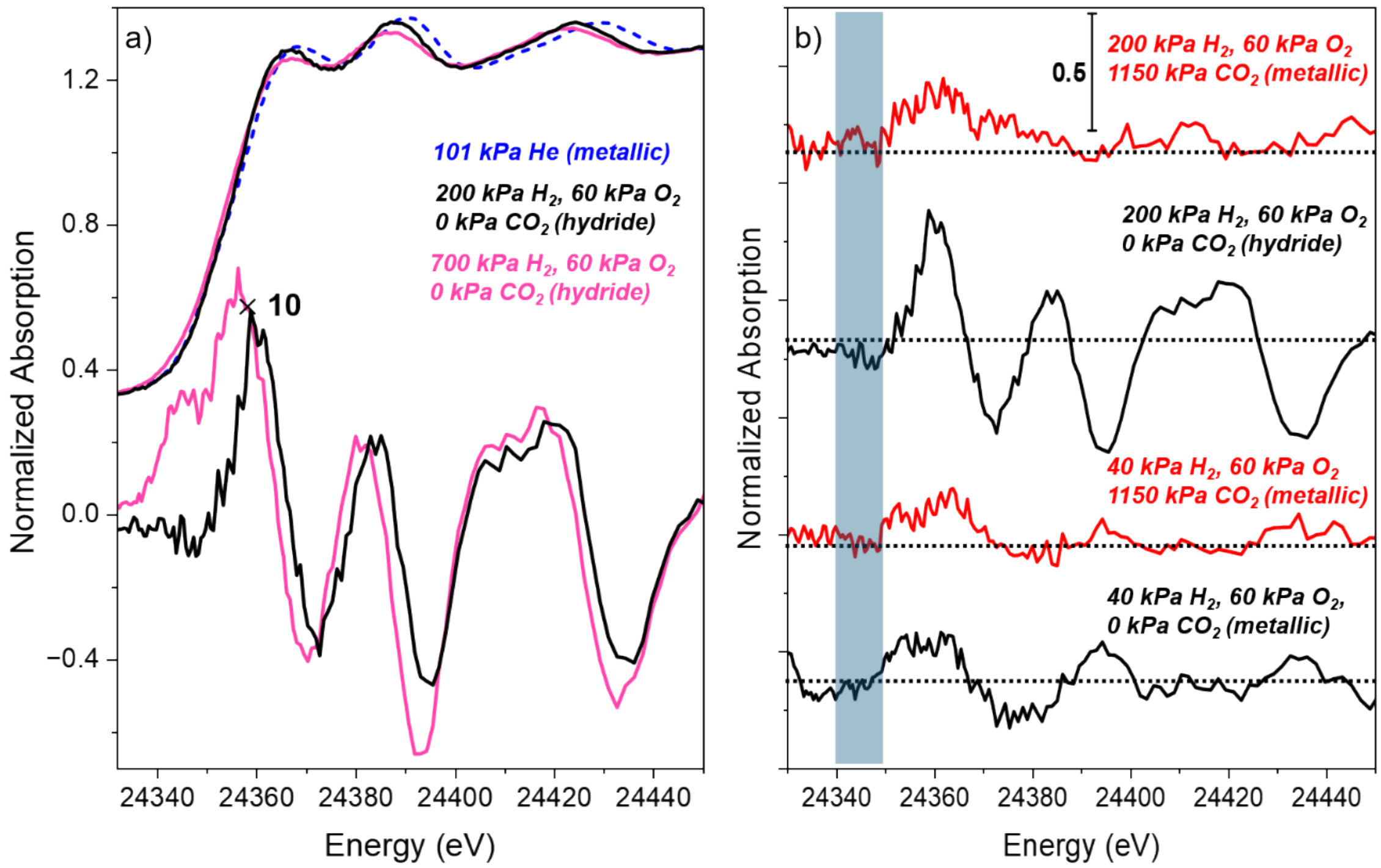

Figure S30. (a) *In situ* Pd K edge XANES spectra of Pd-SiO_2 under 101 kPa He (blue dashed) and *Operando* Pd K edge XANES and ∆XANES of Pd-SiO_2 under increasingly H_2-rich conditions in the absence of CO_2 in H_2O (200-700 kPa H_2, 60 kPa O_2, 0 kPa CO_2, 298 K). (b) *Operando* Pd K edge XANES and ∆XANES of Pd-SiO_2 under H_2- and O_2-rich conditions in the absence and presence of CO_2 (40-200 kPa H_2, 60 kPa O_2, 0 or 1150 kPa CO_2, 298 K). The dashed black line indicates zero. The curves are stacked to aid visual inspection. Pink spectra in (a) is replotted with permission from Adams et al.[1]

Figure S30 shows Pd K edge XANES and ∆XANES of Pd-SiO_2 collected under different conditions to highlight differences in XANES when Pd nanoparticles form a hydride phase (β-PdH_x). Spectra collected under the most H_2-rich conditions by Adams et al. in Figure S30a (pink; 700 kPa H_2, 60 kPa O_2, 0 kPa CO_2, 298 K)[1] shows the fingerprint of ∆XANES for the β-PdH_x phase [replotted with permission]. Spectra collected of Pd-SiO_2 nanoparticles used in this study under H_2-rich conditions in the absence of CO_2 shows similar shifts in XANES to that of a β-PdH_x sample, confirming the formation of a hydride phase for Pd in the absence of CO_2 (Figure S30a, black; 200 kPa H_2, 60 kPa O_2, 0 kPa CO_2, 298 K). The ∆XANES presented in Figure S30b for Pd nanoparticles exposed to O_2-rich conditions and all conditions in the presence of CO_2 do not show the shifts associated with β-PdH_x formation, confirming Pd stays metallic under those conditions. Additionally, the highlighted area in Figure S30b represents the fingerprints corresponding to CO adsorption on Pd nanoparticles, as shown by Usoltsev

et al.[13] The lack of peaks in this area in Figure S30b on Pd nanoparticles under all conditions examined in this study suggest that CO does not form in the presence of CO_2 and adsorb onto Pd.

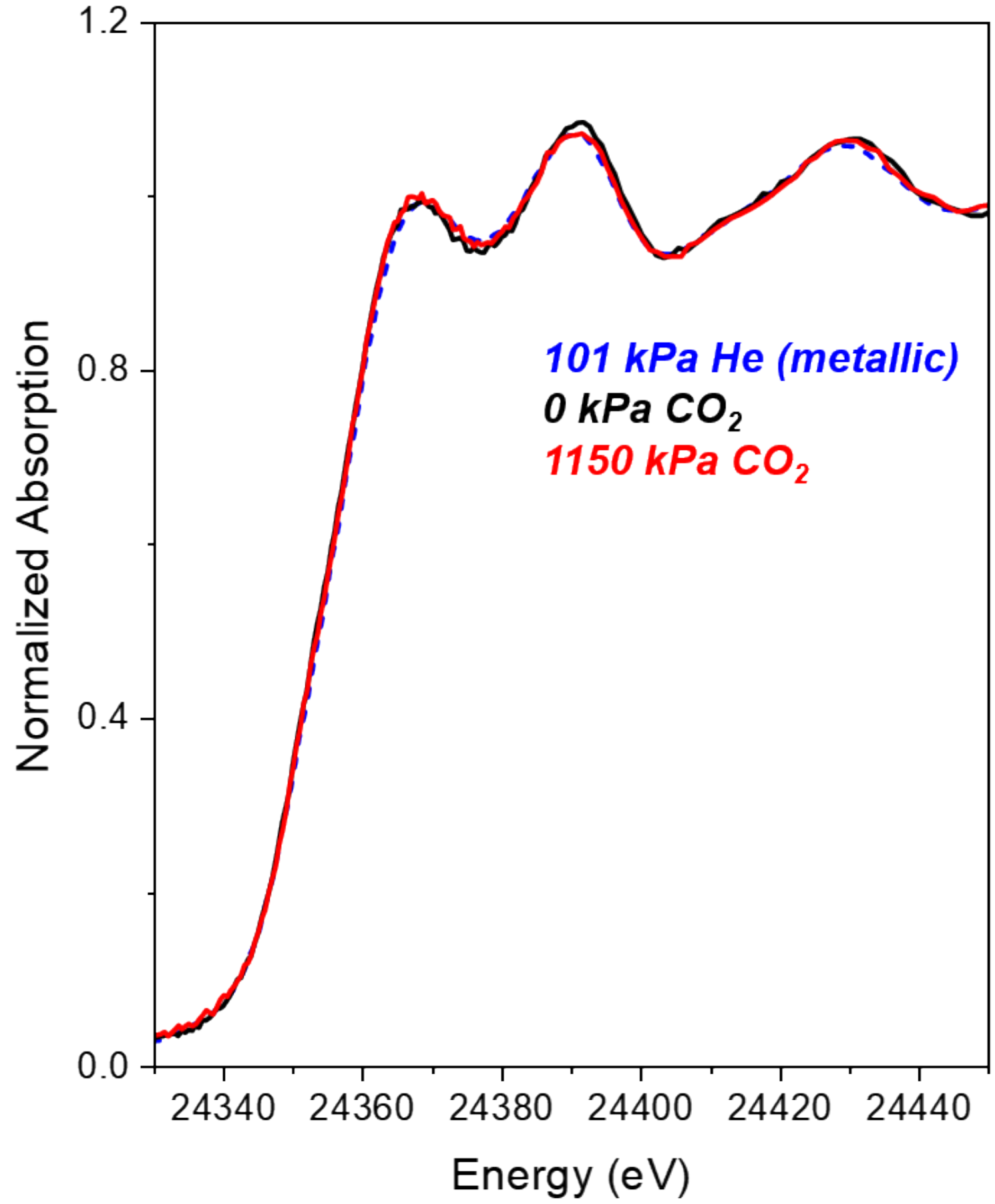

Figure S31. *In situ* Pd K edge XANES spectra of Pd-SiO_2 under 101 kPa He (blue dashed), and *operando* XANES spectra collected under O_2-rich conditions with 0 kPa CO_2 (black), and 1150 kPa CO_2 (red; 40 kPa H_2, 60 kPa O_2, 298 K).

Figure S31 demonstrates the Pd K edge spectra of Pd-SiO_2 under O_2-rich conditions in the absence (black) and presence (red) of CO_2 (40 kPa H_2, 60 kPa O_2, 0 or 1150 kPa CO_2, 298 K) superimposed with the spectrum of Pd-SiO_2 collected in gas-phase under He post *in situ* reduction under 20 kPa H_2 at 423 K (blue dashed; 101 kPa He, 298 K). The lack of spectral changes across different conditions, compared with the spectrum obtained under He, suggests minimal changes in the electronic structure of Pd nanoparticles under O_2-rich conditions when exposed to CO_2.

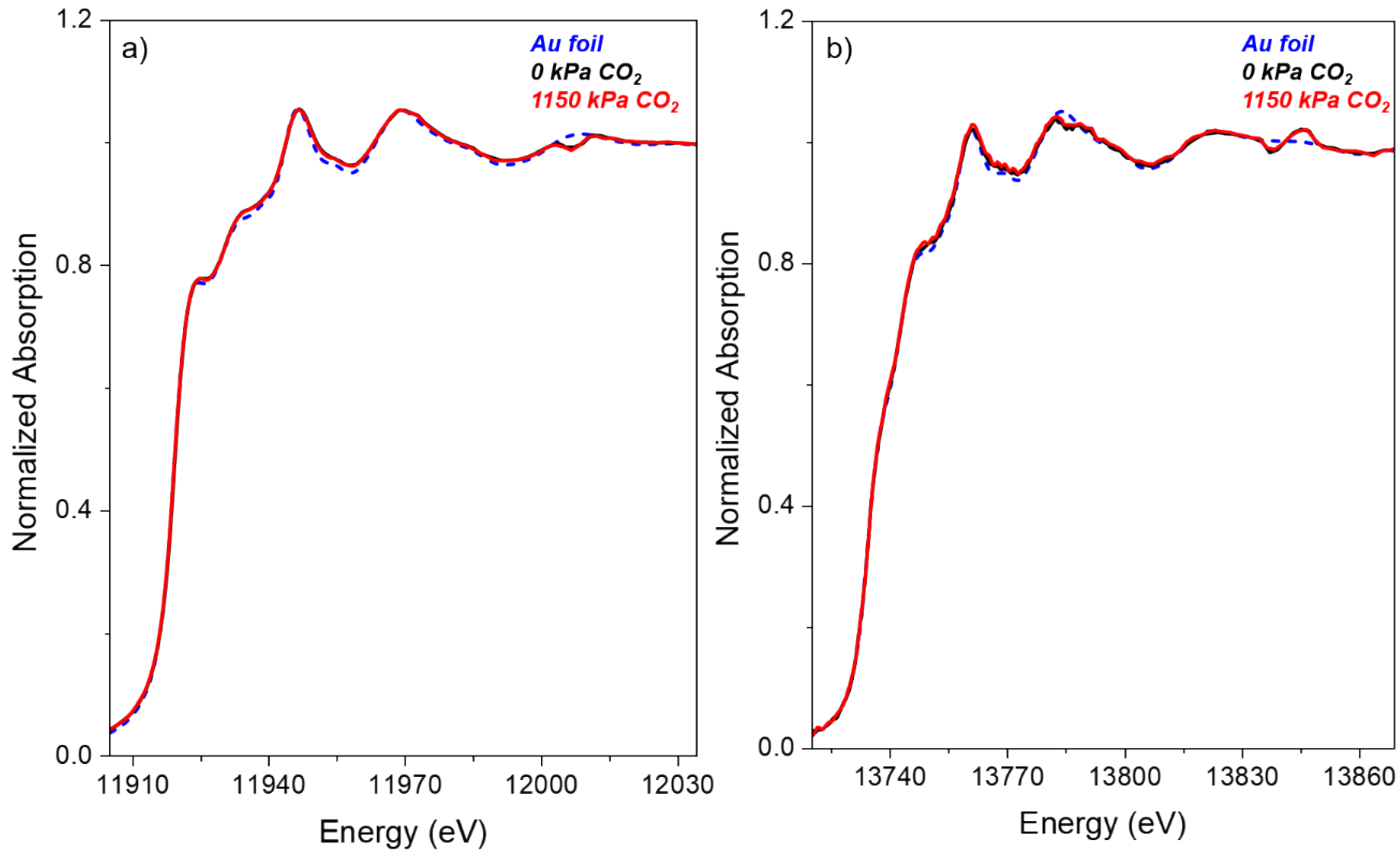

Figure S32. *Operando* Au (a) L_3 (b) L_2 edge XANES spectra of Au-SiO_2 catalysts under O_2-rich thermochemical oxygen reduction conditions in H_2O in the absence (black) and presence (red) of CO_2 (40 kPa H_2, 60 kPa O_2, 0 or 1150 kPa CO_2, 298 K). The blue dotted curve corresponds to the Au foil spectra.

Figure S32 shows the XANES region of the Au L_3 and L_2 edges under O_2-rich conditions in the absence (black) and presence (red) of CO_2 resembles the spectrum of Au foil (40 kPa H_2, 60 kPa O_2, 0 or 1150 kPa CO_2, 298 K). This indicates that there are minimal surface-adsorbed species and that the electronic structure of Au nanoparticles remains unchanged upon CO_2 addition during thermochemical oxygen reduction.

S7. Supplementary Catalyst Potential Data from Open Circuit Potentiometry Measurements

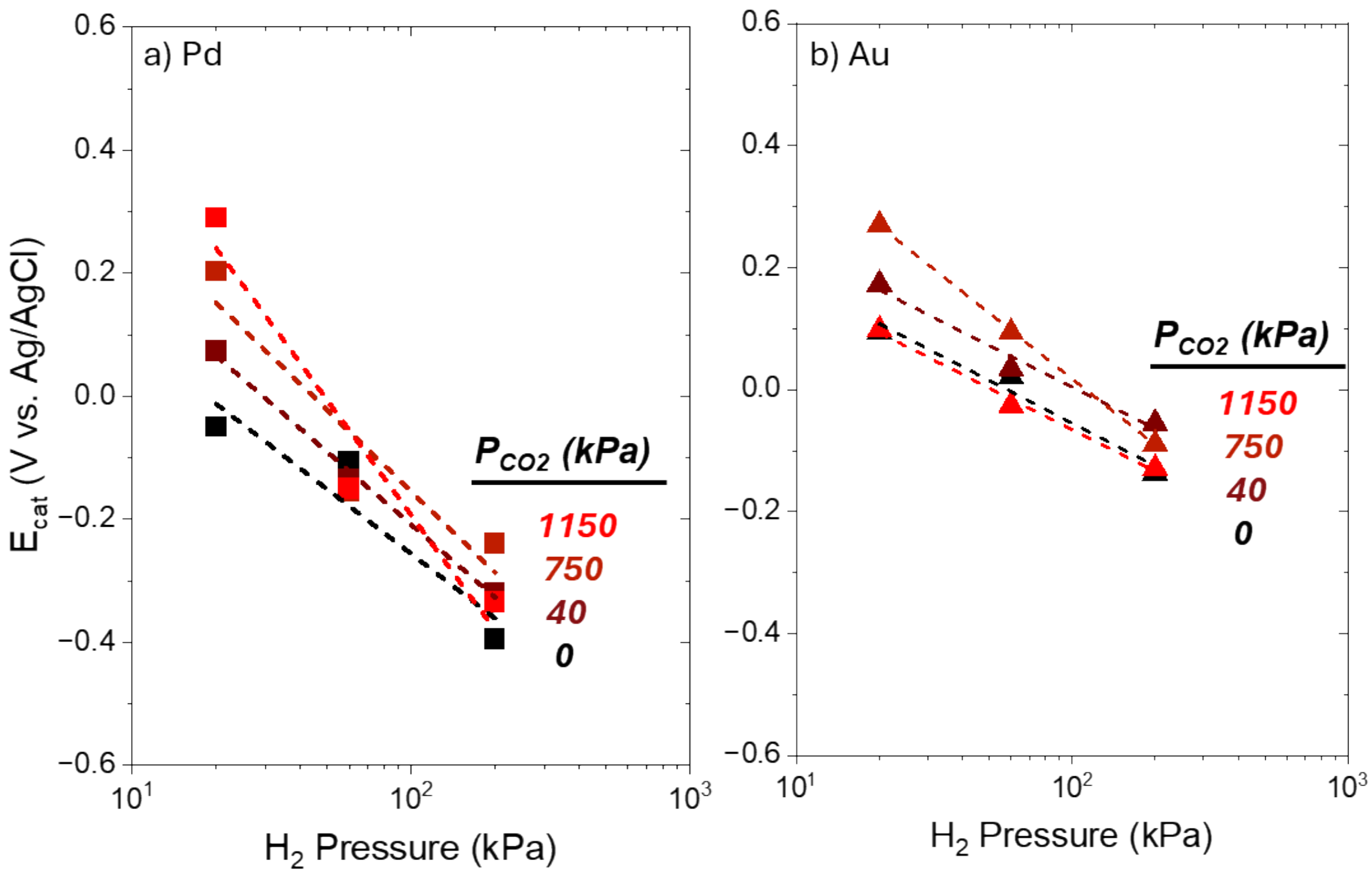

Figure S33. Open-circuit potentiometry measurements of catalyst potentials against the Ag/AgCl reference potential for (a) Pd (□) and (b) Au (▲) foils as functions of H_2 pressure (20–200 kPa of H_2, 60 kPa of O_2, 298 K) at different CO_2 pressures. Nernstian expressions were calculated using the unit activities of H_3O^+ and H_2O species (Section 2.6).

Figure S33 shows E_{cat} values for Pd and Au foils versus the Ag/AgCl reference electrode changes at different conditions of H_2 and CO_2 pressures when pH is not taken into consideration. Pd and Au E_{cat} values across all CO_2 pressures decrease with H_2 pressures and deviate from the slope for Nernstian HOR and ORR behavior (Figure 5). This suggests that combinations of half reactions contribute to the measured E_{cat}. The slopes of E_{cat} values as a function of H_2 pressure do not vary significantly with different CO_2 pressures when pH changes are not accounted for. Conversely, E_{cat} values plotted against the RHE scale when pH is considered show significant change in slope with increasing CO_2 pressure (Figure 5). When calculated against the Ag/AgCl scale, E_{cat} values increase and become more positive with increasing CO_2 pressures, which is opposite to the trend observed in Figure 5 when pH changes are taken into calculations, and E_{cat} is calculated against the RHE scale. This emphasizes the need to measure *in situ* pH and account for pH changes across reaction conditions when comparing E_{cat} to draw meaningful conclusions.

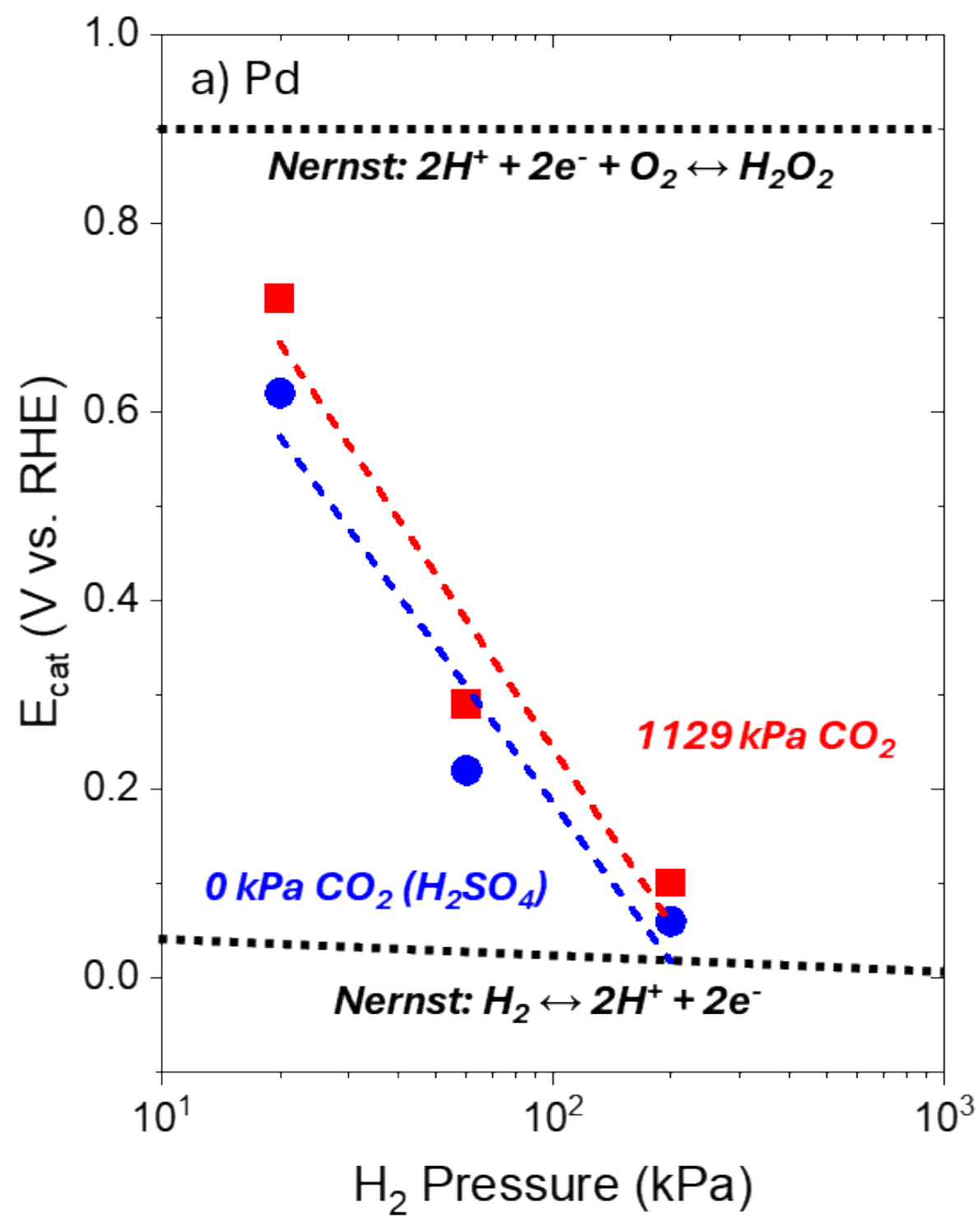

Figure S34. Open-circuit potentiometry measurements of catalyst potentials for (a) Pd (□) and (b) Au (▲) foils as functions of H_2 pressure (20–200 kPa of H_2, 60 kPa of O_2, 298 K) at 1129 kPa CO_2 (red) corresponding to a pH of 3 and with sulfuric acid (blue ●) corresponding to a pH of 3. Nernstian expressions were calculated using the unit activities of H_3O^+ and H_2O species (Section 2.6).

Figures S34 and S35 show E_{cat} values for Pd and Au foils versus the RHE scale and the Ag/AgCl reference electrodes, respectively, and the dependence of these potentials on H_2 pressure, with pH fixed at 3 by the addition of either CO_2 or H_2SO_4. On both Pd and Au, E_{cat} values vary similarly with increasing H_2 pressure with both CO_2 and H_2SO_4 acidification. On Pd, E_{cat} values with H_2SO_4 are marginally lower or more negative across different H_2 pressures than with CO_2. This suggests adding H_2SO_4 favors the HER reaction more than CO_2, implying that H_2SO_4 increases [H^+] and in turn, hydrogen coverage on Pd surfaces to a larger extent than CO_2. However, on Au, the opposite trend is observed, where E_{cat} values are higher with H_2SO_4 than CO_2. This indicates that CO_2 addition increases [H^+] and hydrogen coverage to a greater extent than H_2SO_4 addition on Au surfaces. More broadly, the E_{cat} values for both Pd and Au in the presence of CO_2 are different from those when H_2SO_4 is added at similar pH values, which implies that CO_2 modifies the favorability of half-reactions and the O_2 reduction reaction mechanism differently than H_2SO_4, aligning with semi-batch kinetic results shown in Figures S12, S13 and Tables S4, S5.

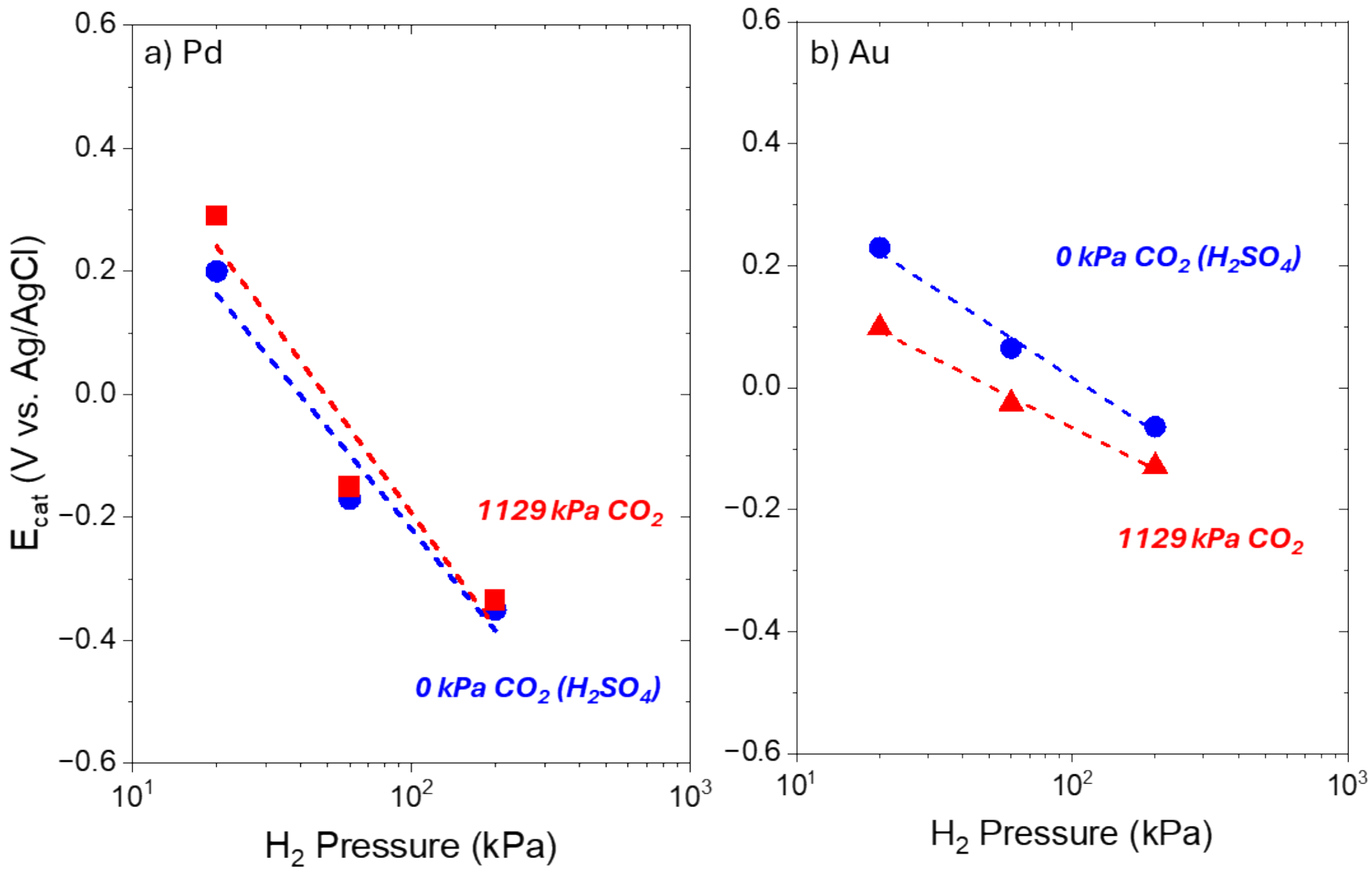

Figure S35. Open-circuit potentiometry measurements of catalyst potentials against the Ag/AgCl reference potential for (a) Pd (□) and (b) Au (▲) foils as functions of H_2 pressure (20–200 kPa of H_2, 60 kPa of O_2, 298 K) at 1129 kPa CO_2 (red) corresponding to a pH of 3 and with sulfuric acid (blue ●) corresponding to a pH of 3.

S8. Supplementary Kinetic Measurements on Pd Nanoparticles

Table S12. Steady state H_2O_2 formation rates and selectivities on Pd-SiO_2 catalysts in the presence and absence of CO_2 and following spent catalyst regeneration after CO_2 exposure (200 kPa H_2, 60 kPa O_2, 278 K).

Conditions	H_2O_2 Formation Rate (mol H_2O_2)(mol Pd^{-1} s^{-1})	H_2O_2 Selectivity (%)
0 kPa CO_2<br>Fresh	0.114 ± 0.022	55 ± 4
1150 kPa CO_2<br>Fresh	0.170 ± 0.003	90 ± 8
0 kPa CO_2<br>Spent; Post oxidation & reduction	0.035 ± 0.003	22 ± 1

Table S12 shows steady-state H_2O_2 formation rates and selectivities on Pd-SiO_2 under H_2 rich conditions for fresh Pd-SiO_2 catalysts in the presence and absence of CO_2 from Figure 1 and Table 1 in the main text (200 kPa H_2, 60 kPa O_2, 0 or 1150 kPa CO_2, 278 K). Table S12 also shows H_2O_2 rates and selectivities of Pd-SiO_2 following oxidation at 1073 K after exposure to CO_2 to remove adsorbed organics (Figure S26), and subsequent reduction at 573 K to reduce Pd nanoparticles (regenerated Pd). The steady-state rates and selectivities of the regenerated Pd catalysts are lower than those exhibited by fresh Pd catalysts before CO_2 exposure. Oxidation at 1073 K leads to nanoparticle agglomeration, resulting in an increase in average nanoparticle diameter from 2 to 12 nm. The lower rates and selectivities observed on the regenerated Pd catalysts resemble those observed on fresh Pd nanoparticles of 12 nm following similar thermal treatments from prior work.[4] These results confirm that the removal of CO_2-derived organics through high temperature oxidation eliminate the irreversible increases in H_2O_2 rates and selectivities observed on Pd nanoparticles after CO_2 exposure (Figure 1, Table 1).

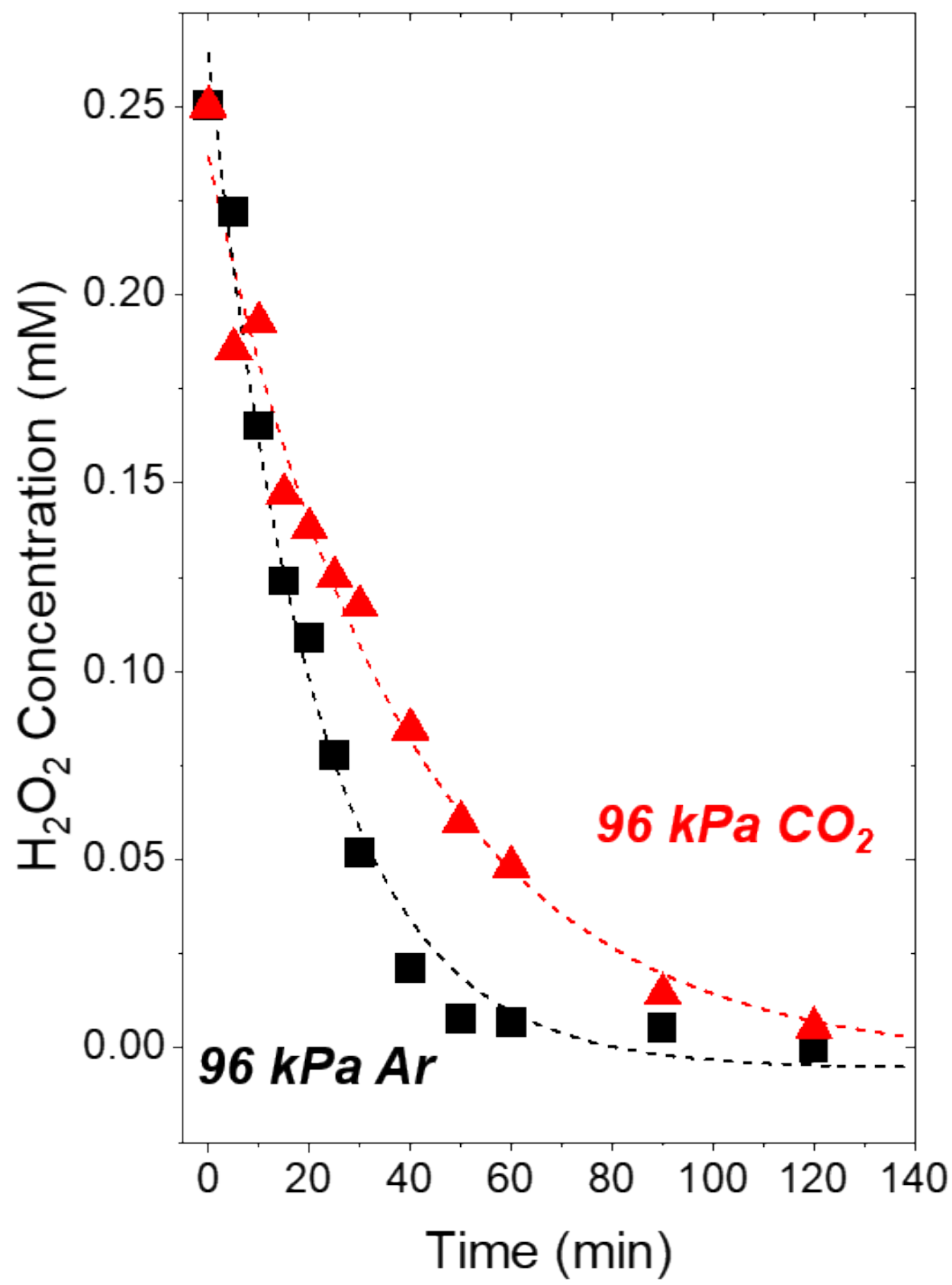

Figure S36. H_2O_2 concentration over 2 hours when reacting H_2 with 0.25 mM H_2O_2 solution under Ar (black) or CO_2 (red) over 50 mg of 2 nm Pd-SiO_2 (5 kPa H_2, 96 kPa Ar or CO_2, 80 cm^3 of 2.5 mM H_2O_2, 298 K). Dashed lines fitted to guide the eyes.

Figure S36 shows the H_2O_2 concentration profile over time on Pd-SiO_2 catalysts in 0.25 mM H_2O_2 solution upon exposure to 5 kPa H_2 to probe H_2O_2 hydrogenation rates in the absence and presence of CO_2. H_2O_2 concentration in the presence of H_2 and Ar rapidly decreases due to hydrogenation to H_2O to reach ~0 mM in ~50 mins. However, H_2O_2 hydrogenation is slower in the presence of CO_2 and takes ~120 mins to reach ~0 mM. This confirms that CO_2 and acidic environments decrease H_2O_2 decomposition and hydrogenation on Pd nanoparticles.

References

(1) Adams, J. S.; Chemburkar, A.; Priyadarshini, P.; Ricciardulli, T.; Lu, Y.; Maliekkal, V.; Sampath, A.; Winikoff, S.; Karim, A. M.; Neurock, M. Solvent molecules form surface redox mediators in situ and cocatalyze O2 reduction on Pd. *Science* **2021**, *371* (6529), 626-632.
(2) Adams, J. S.; Chen, H.; Ricciardulli, T.; Vijayaraghavan, S.; Sampath, A.; Flaherty, D. W. Distinct site motifs activate O_2 and H_2 on supported Au nanoparticles in liquid water. *ACS catalysis* **2024**, *14* (5), 3248-3265. DOI: 10.1021/acscatal.3c05072.
(3) Sander, R. Compilation of Henry's law constants for inorganic and organic species of potential importance in environmental chemistry. Max-Planck Institute of Chemistry, Air Chemistry Department Mainz, Germany: 1999.
(4) Vijayaraghavan, S.; Waldt, C. T.; Kumar, R.; Rathi, N.; Hibbitts, D. D.; Flaherty, D. W. Nanoparticle size and composition of PdAu catalysts govern the structure and kinetics of H_2O_2 formation through O_2 reduction. *Journal of Catalysis* **2025**, 116649. DOI: 10.1016/j.jcat.2025.116649.
(5) Wilson, N. M.; Flaherty, D. W. Mechanism for the Direct Synthesis of H2O2 on Pd Clusters: Heterolytic Reaction Pathways at the Liquid–Solid Interface. *Journal of the American Chemical Society* **2016**, *138* (2), 574-586. DOI: 10.1021/jacs.5b10669.
(6) Ricciardulli, T.; Gorthy, S.; Adams, J. S.; Thompson, C.; Karim, A. M.; Neurock, M.; Flaherty, D. W. Effect of Pd Coordination and Isolation on the Catalytic Reduction of O_2 to H_2O_2 over PdAu Bimetallic Nanoparticles. *Journal of the American Chemical Society* **2021**, *143* (14), 5445-5464. DOI: 10.1021/jacs.1c00539.
(7) Iwasita, T.; Rodes, A.; Pastor, E. Vibrational spectroscopy of carbonate adsorbed on Pt (111) and Pt (110) single-crystal electrodes. *Journal of Electroanalytical Chemistry* **1995**, *383* (1-2), 181-189. DOI: 10.1016/0022-0728(94)03708-B.
(8) Arihara, K.; Kitamura, F.; Ohsaka, T.; Tokuda, K. Characterization of the adsorption state of carbonate ions at the Au(111) electrode surface using in situ IRAS. *Journal of Electroanalytical Chemistry* **2001**, *510* (1-2), 128-135. DOI: 10.1016/S0022-0728(01)00498-3.
(9) Moradzaman, M.; Mul, G. Infrared analysis of interfacial phenomena during electrochemical reduction of CO_2 over polycrystalline copper electrodes. *ACS catalysis* **2020**, *10* (15), 8049-8057. DOI: 10.1021/acscatal.0c02130.
(10) Kloprogge, J. T.; Wharton, D.; Hickey, L.; Frost, R. L. Infrared and Raman study of interlayer anions CO_3^{2-}, NO_3^-, SO_4^{2-} and ClO_4^- in Mg/Al-hydrotalcite. *American Mineralogist* **2002**, *87* (5-6), 623-629.
(11) Miyake, H.; Okada, T.; Samjeské, G.; Osawa, M. Formic acid electrooxidation on Pd in acidic solutions studied by surface-enhanced infrared absorption spectroscopy. *Physical Chemistry Chemical Physics* **2008**, *10* (25), 3662-3669.

(12) Jeroro, E.; Vohs, J. M. Zn Modification of the Reactivity of Pd(111) Toward Methanol and Formaldehyde. *Journal of the American Chemical Society* **2008**, *130* (31), 10199-10207. DOI: 10.1021/ja8001265.
(13) Usoltsev, O. A.; Skorynina, A. A.; Protsenko, B. O.; Martin-Diaconescu, V.; Pellegrini, R.; Soldatov, A. V.; van Bokhoven, J.; Bugaev, A. L. Evolution of surface and bulk structure of supported palladium nanoparticles by in situ X-ray absorption and infrared spectroscopies: Effect of temperature, CO and CH_4 gas. *Applied Surface Science* **2023**, *614*, 156171. DOI: https://doi.org/10.1016/j.apsusc.2022.156171.
